# Analysis of the role of Purα in the pathogenesis of Alzheimer's disease based on RNA-seq and ChIP-seq

**DOI:** 10.1038/s41598-021-90982-1

**Published:** 2021-06-09

**Authors:** Xiaoguang Shi, Shuanglai Ren, Bingying Zhang, Shanshan Guo, Wenxin He, Chengmin Yuan, Xiaofan Yang, Kevin Ig-lzevbekhai, Tao Sun, Qinwen Wang, Jianqi Cui

**Affiliations:** 1 Ningxia Key Laboratory of Cerebrocranial Diseases, Incubation Base of the National Key Laboratory, Ningxia Medical University, Yinchuan, China; 2 Zhejiang Provincial Key Laboratory of Pathophysiology, School of Medicine, Ningbo University, Ningbo, Zhejiang China; 3 Department of Neurology, Hongqi Hospital Affiliated to Mudanjiang Medical University, Mudanjiang, China; 4 Perelman School of Medicine, University of Pennsylvania, Philadelphia, PA USA

**Keywords:** Molecular biology, Neuroscience

## Abstract

Purine rich element binding protein A (Purα), encoded by the Purα gene, is an important transcriptional regulator that binds to DNA and RNA and is involved in processes such as DNA replication and RNA translation. Purα also plays an important role in the nervous system. To identify the function of Pura, we performed RNA sequence (RNA-seq) analysis of Purɑ-KO mouse hippocampal neuron cell line (HT22) to analyze the effect of Purα deletion on neuronal expression profiles. And combined with ChIP-seq analysis to explore the mechanism of Purα on gene regulation. In the end, totaly 656 differentially expressed genes between HT22 and Purα-KO HT22 cells have been found, which include 7 Alzheimer’s disease (AD)-related genes and 5 Aβ clearance related genes. 47 genes were regulated by Purα directly, the evidence based on CHIP-seq, which include Insr, Mapt, Vldlr, Jag1, etc. Our study provides the important informations of Purα in neuro-development. The possible regulative effects of Purα on AD-related genes consist inthe direct and indirect pathways of Purα in the pathogenesis of AD.

## Introduction

Purα is a 322-amino acid protein encoded by Purα^1^. It can bind to purine rich DNA or RNA sequences, and promoter regions of some genes in order to form multimeric complexes, and can also interact with other transcription factors^2^. Purα can promote transcription of some genes, such as TNF-α^3^, myelin basic protein^4^, placental lactogen^5^, and PDGF- Protein A^6^, etc. Furthermore, it can also inhibits some genes’ expression, such as fas^7^, α-actin^8^, amyloid-β precursor protein^9^ and CD43^10^. Although Purα is widely expressed throughout the whole body in human^11^, its main role is to maintain the stability of the nervous system^12^. Mice lacking the Purα gene could develop severe tremors and spontaneous seizures 2 weeks after birth and died at about 4 weeks of age^13^.

In the past few years, we have focused on the roles of Purα in the nervous system, including repairing DNA damage in neurons^14^ and influencing Alzheimer's disease pathogenesis^15^. Recent technological developments in the life sciences research have allowed new insights into the functions of Purα. In current study, we performed RNA-seq and ChIP-seq analysis on Purα knockout cell lines based on CRISPR/Cas9 gene editing technology. We hope to improve the understanding of Purα through these investigations.

## Methods

### Cell culture

The HT22 cell line (Mouse Hippocampal Neuronal Cell Line) was maintained in our laboratory. The Purα-KO cell line was independently constructed and validated by the laboratory, based on CRISPR/Cas9, and screened for single cell cultured monoclonal cell lines^14^. The cells were cultured in an incubator at 37 °C. Nutrient composition: 89% DMEM (Bioind, USA), 10% FBS (Bioind, USA) and 1% penicillin–streptomycin (Solarbio, China).

### RNA-seq

Whole genome RNA was extracted using the TRIZOL method, RNA concentration was determined using NanoDrop 2000 (Thermo), and RNA integrity was detected using an Agilent Bioanalyzer 2100 system (Agilent Technologies, CA, USA). The sequencing library was built using the NEBNext Ultra RNA Library Preparation Kit for Illumina (NEB, USA) and library quality was assessed on an Agilent Bioanalyzer 2100 system. Clustering was performed using the TruSeq PE Cluster Kit v4-cBot-HS (Illumia), after which the library preparations were sequenced on an Illumina Hiseq Xten platform and the readings at the paired ends were generated.

### ChIP-seq

ChIP immunoprecipitation was performed using a Pierce Agarose ChIP Kit (Catalog number: 26156, Thermo USA). The antibody used to capture Purα was purchased from SANTA CRUZ (sc-130397). HT22 cells were seeded in a 10 cm diameter petridish and subjected to ChIP immunoprecipitation with cell growth to 95% confluence. The ChIP immunoprecipitation operation was carried out according to the instructions. The main processes included formaldehyde cross-linking, sonication, enrichment of the target protein with the magnetic beads coated with the antibody, de-crosslinking, and recovery of the DNA fragment. The library was constructed using the ChIP-seq Library Prep Master Mix Set for Illumina (NEB, USA), the main processes including end repair, add A (aenosine) to the 3’end, ligate adaptation, gel purification and size selection, PCR amplification. Sequencing was performed using the Illumina HiSeq 2500 sequencing platform.

### Sequencing data quality control

Raw data in the fastq format (original reads) removed the purchase of ploy-N and low-quality reads to get clean data (clean reads). At the same time, Q20, Q30, GC content and sequence repeat levels of clean data were calculated (Table S5). All downstream analyses are based on high quality, clean data.

### Differential gene acquisition

The sequencing fastq data were compared to the mouse reference gene (GRCm38/mm10) using hisat2. The SAMtools tool was used to convert the obtained SAM file to a BAM file. The readings were counted using the htseq-count tool. The read matrices were analyzed using the edgeR tool to obtain differential genes with a fold multiple of ≥ 2 and FDR < 0.05 as a screening criterion. Since no biological duplication was set in this study, CORNAS (overlying RNA-Seq) was used for differential gene screening. Alpha and FDR use default values (Alpha = 99%, FDR = 1.5). 1165 differential genes were obtained by edgeR analysis, and 676 differential genes were obtained by CORNAS analysis. The intersection of the two results was taken as the final differential gene, and a total of 656 genes were obtained (Table S1).

### ChIP-seq data analysis

Clean reads were compared with reference genomic sequences to obtain alignment information of ChIP-seq DNA (bowtie2); alignment peak position and alignment intensity information (MACS) were found by comparing position information of reads on the genome. We use MEME-ChIP software to identify and annotate motif, and use MEME and Dreme to detect the significant motif sequences in the peak sequence, and then use Tomtom software to compare the obtained motif sequences with known motif databases.

### Other tools

Wayne maps and gene maps on chromosomes were constructed using TBtools (https://github.com/CJ-Chen/TBtools). GO, KEGG analysis was performed using the Omic Share tools, a free online platform for data analysis (http://www.omicshare.com/tools).

## Results

### RNA-seq analysis of Purα-KO cell line

**Figure 1:**
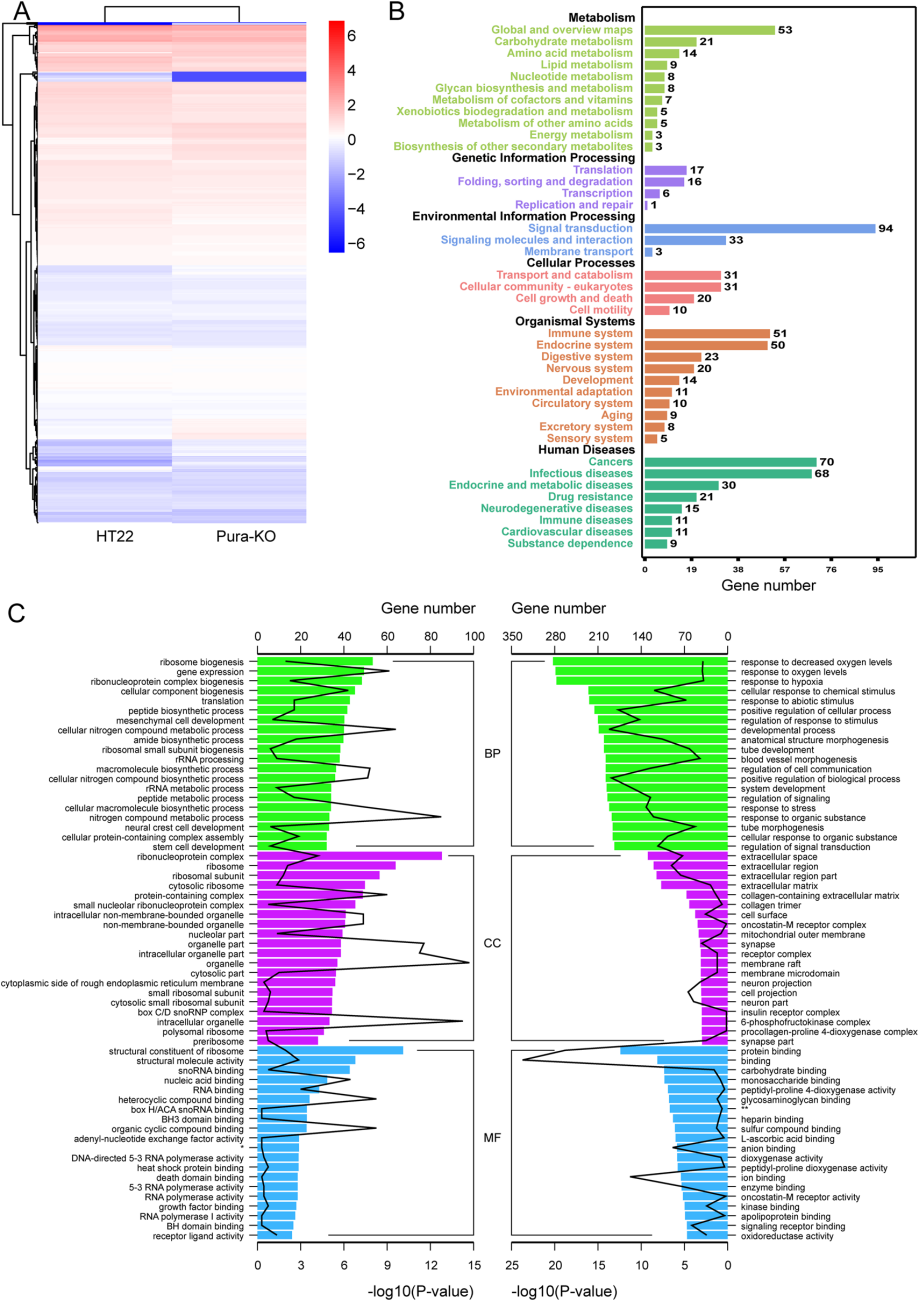
Differential gene enrichment analysis. (**A**) The difference between the differential genes in the HT22 group and the Purɑ-KO group is shown in the form of a heat map. (**B**) Histogram results of KEGG enrichment analysis of differential genes. The abscissa is the number of genes and the ordinate is the enrichment result. (**C**) GO enrichment of differential genes. The left aspect is the result of up-regulated gene enrichment and the right represents down-regulation. *RNA polymerase II sequence-specific DNA-binding transcription factor binding. **oxidoreductase activity, acting on paired donors, with incorporation or reduction of molecular oxygen, 2-oxoglutrate as one donor, and incorporation of one atom each of oxygen into both donors.

In our experiments, RNA-seq analysis was first performed on HT22 cells knocked out of Purα using CRISPR/Cas9. Comparing the Purα-KO and HT22 expression profiles, we found a total of 656 differential genes (Fig. 1A). The down-regulated genes are predominant 488/656 (Table S1), suggesting Purα plays a major role in promoting gene expression. The top 5 down-regulated and up-regulated genes are listed in Table 1. Purα is an important transcriptional activator, which means that Purα knockout has a considerable impact on many metabolic pathways involved in growth and development, such as pathways in cancer, PI3K-Akt signaling, and cytokine-cytokine receptor interaction (Fig. 1B). The down-regulated genes are 488/656 (Table S1), indicating that Purα plays a major role in promoting gene expression. In order to explore the biological functions of differential genes, we performed GO annotations on the up-regulated and down-regulated genes. The results showed that the down-regulated genes were involved in neuronal structure, neuronal projection, response to oxygen, and positive regulation of cellular processes (Fig. 1C). These findings highlight the vital role of Purα in the growth and development of neurons. Meanwhile, pathways related to neurodevelopment, such as neurotrophin signal transduction and axon guidance pathways, are also down-regulated. In addition, Knockout of Purα resulted in the up-regulation of 168 genes, which are involved in the biogenesis of ribosomes, ribonucleoprotein complexes, and structural components of ribosomes, based on GO analysis. (Fig. 1C).

**Table 1:** The list of top5 up-regulated and down-regulated genes.

Gene ID	Gene name	HT22 (count)	Purα-ko (count)	FDR	log2FC	Regulated
ENSMUSG00000017400	Stac2	573	1	4.79E–64	− 9.02	Down
ENSMUSG00000040026	Saa3	4757	329	8.13E–56	− 3.89	Down
ENSMUSG00000020826	Nos2	1910	138	5.23E–49	− 3.83	Down
ENSMUSG00000064246	Chil1	279	1	6.98E–42	− 7.99	Down
ENSMUSG00000021876	Rnase4	221	0	1.88E–37	− 10.81	Down
ENSMUSG00000109724	–	22	244	5.70E–21	3.43	Up
ENSMUSG00000019997	Ctgf	314	1120	7.59E-13	1.80	Up
ENSMUSG00000063632	Sox11	59	281	1.54E–12	2.21	Up
ENSMUSG00000039316	Rftn1	5	71	3.54E–10	3.76	Up
ENSMUSG00000068113	Gm4907	16	105	1.63E–09	2.67	Up

### Purα regulates the protein expression of neurons in early development

**Figure 2:**
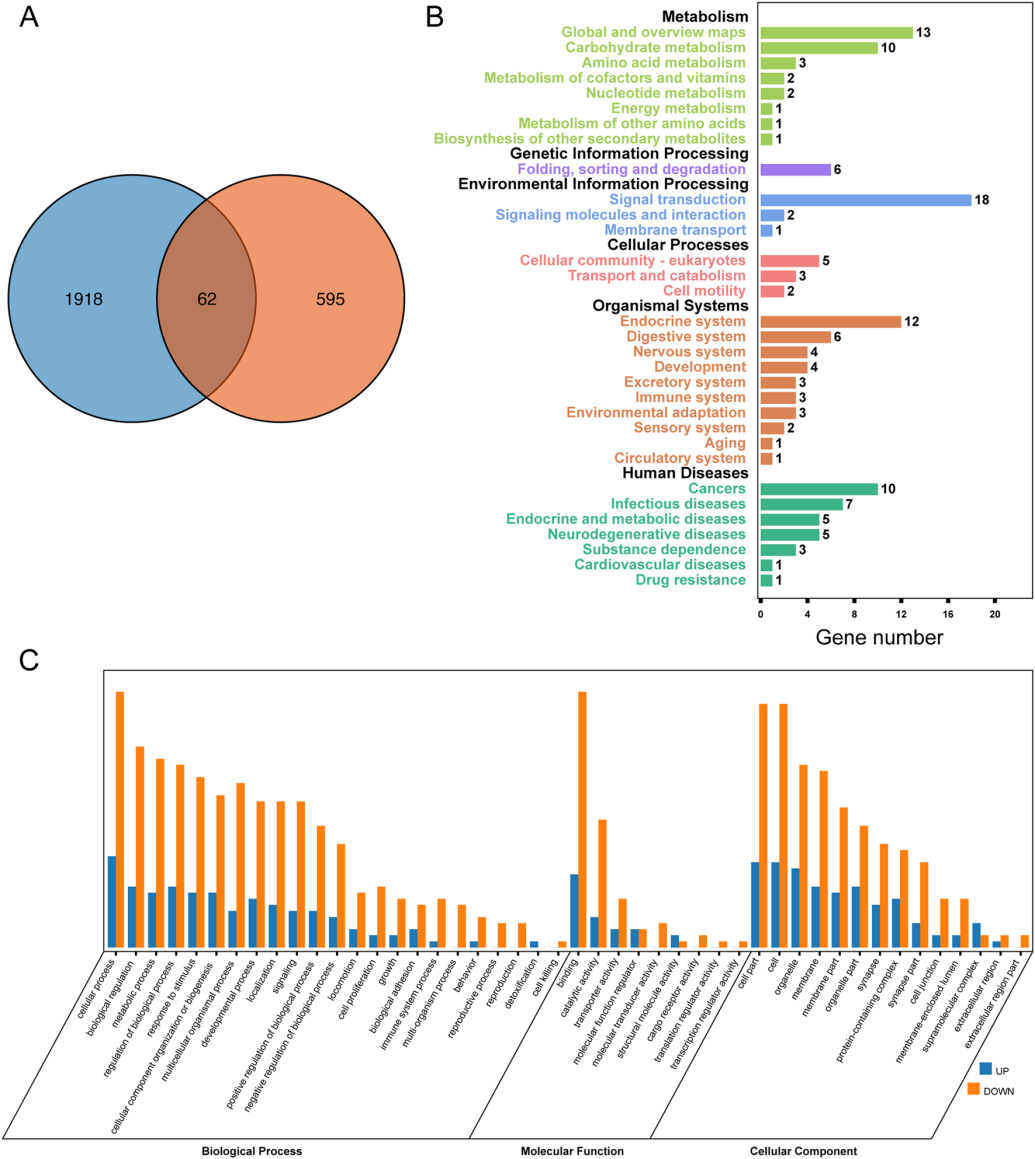
Comparison of early brain protein expression profiles with mice. (**A**) Compared with the brain protein expression profiles of mice from the 9-day postnatal experiment from the Gonzalez-Lozano study (blue), it was found that expression of a total of 62 genes appeared in the differential gene results of Purɑ-KO (Orange). These 62 genes may be key regulators of Purɑ in early brain development. (**B**) Histogram results of KEGG enrichment analysis of differential genes. The abscissa is the number of genes and the ordinate is the enrichment item. (**C**) GO enrichment of differential genes. Orange indicates genes that are up-regulated and blue indicates down-regulation.

To clarify the trend of Purα in postnatal mice, we found that Purα expression was reduced in 280-day-old mice (adults) compared to 9- and 15-day mice from Gonzalez-Lozano's study^16^. This result would suggest that the main function of Purα occurs in infancy and does not persist in adulthood. To explore the role of Purα in the early development of the brain, we compared the Purα-KO gene expression profile with the protein profile from Gonzalez-Lozano’s study^16^. We noticed a duplication *of* 62 proteins (Table S2), suggesting that the cause of early death in Purα-KO mice may be included in these 62 proteins. We further functionalized 62 genes and found that these genes were involved in many functional and metabolic pathways (based on GO and KEGG analysis), including neuronal structure (GO: 0097458), neuronal projections (GO: 0043005), and nervous system. They were also involved in development (GO: 0007399), neurotransmitter levels (GO: 0001505), glycolysis/gluconeogenesis (mmu04066), HIF signaling pathway (mmu04066), carbon metabolism (mmu01200) (Fig. 2).

### The effect of Purα on AD-related gene expression

**Figure 3:**
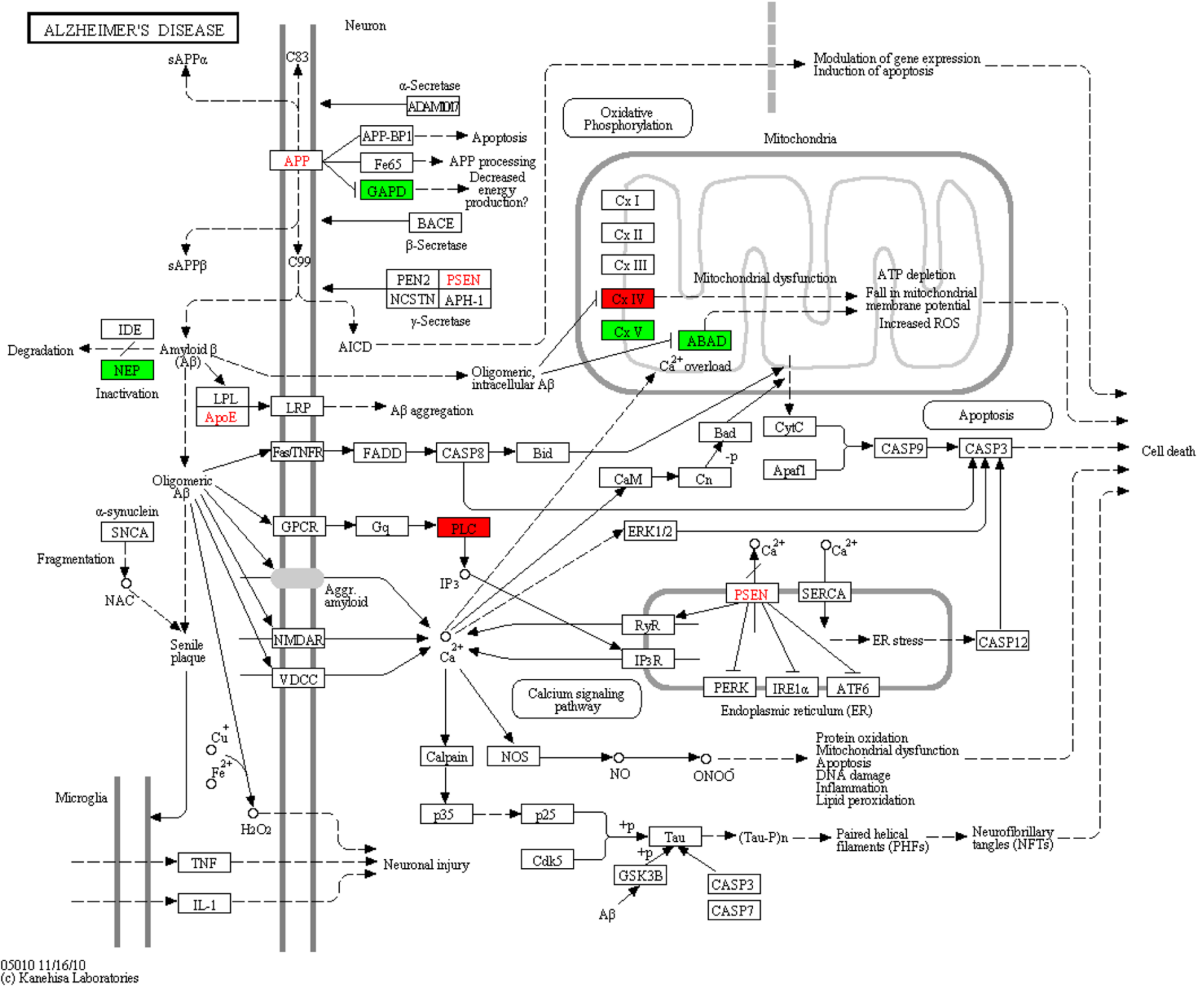
Distribution of differential genes in Alzheimer disease pathway. Through KEGG annotation analysis, we found 7 genes related to the Alzheimer disease pathway. There are 4 core factors in Alzheimer disease, which are APP, PSEN, ApoE, and Tau, shown in red font. The seven genes enriched were Lpl (LPL), Mapt (Tau), Mme (NEP), Ndufa3 (Cx I), Lrp1 (LRP), Gapdh (GAPD), and mt-Co3 (Cx IV). Green boxes indicate down-regulation of related genes, and red indicates up-regulation.

In our study we deliberately focused on the expression of APP after Purα knockout, but unfortunately the knockout of Purα seems to have no effect on APP or APP mRNA expression. This result is inconsistent with the previous study reported by Nune Darbinian et al.^9^ and the result also highlights additional complexities of Purɑ in AD pathogenesis. Based on RNA-seq analysis, we found that 7 genes are enriched in Alzheimer disease (Table 2, Fig. 3), and 5 genes are enriched in A β (amyloid-beta) clearance (Table 3).

**Table 2:** Differential gene enriched in Alzheimer's disease.

Gene ID	Gene name	HT22 (count)	Purα-ko (count)	FDR	log2FC	Regulated
ENSMUSG00000015568	Lpl	1138	554	4.20E–05	− 1.08	Down
ENSMUSG00000018411	Mapt	25	87	8.02E–05	1.76	Up
ENSMUSG00000027820	Mme	361	86	7.48E–13	− 2.11	Down
ENSMUSG00000035674	Ndufa3	621	1178	0.001	0.89	Up
ENSMUSG00000040249	Lrp1	7916	4904	0.006	− 0.73	Down
ENSMUSG00000057666	Gapdh	6112	3641	0.003	− 0.78	Down
ENSMUSG00000064358	mt-Co3	1381	778	0.001	− 0.87	Down

**Table 3:** Differential gene enriched in amyloid-beta clearance.

Gene ID	Gene name	HT22 (count)	Purα-ko (count)	FDR	log2FC	Regulated
ENSMUSG00000027820	Mme	361	86	7.48E–13	− 2.11	Down
ENSMUSG00000024164	C3	6635	1260	9.59E–26	− 2.43	Down
ENSMUSG00000040249	Lrp1	7916	4904	0.006	− 0.73	Down
ENSMUSG00000005534	Insr	657	359	0.001	− 0.91	Down
ENSMUSG00000023992	Trem2	83	6	8.95E–12	− 3.80	Down

### Combining Chip-Seq to analyze the possible mechanism of Purα in regulating gene expression

**Figure 4:**
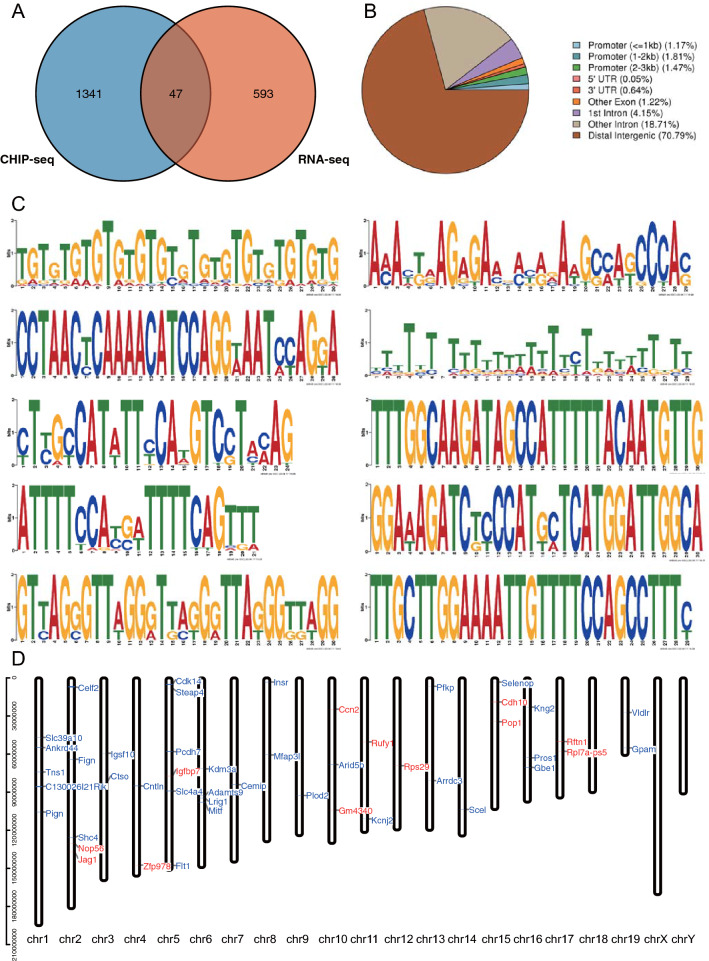
ChIP-seq analysis. (**A**) ChIP-seq detected a total of 1388 genes likely to bind to Purα. Compared with 656 differential genes obtained by RNA-seq, a total of 47 genes were obtained. (**B**) Peak region annotation classification of Purα-bound DNA. (**C**) ChIP-seq analysis yields a total of 10 motifs with which Purα may bind, and the logos are sorted in order. (**D**) The position and expression of the 47 genes obtained from A on the chromosome. The blue represents gene down-regulation and the red represents up-regulation.

We have enriched 656 differential genes that may be regulated by Purα based on RNA-seq. Not all genes are directly regulated by Purα. In order to clarify the regulatory mechanism of Purα on genes, we analyzed the DNA fragments that may be directly bound to Purα by ChIP-seq, and found that Purα can bind to 1389 genes (Table S3). To further analyze the regulation of Purα on genes, we combined ChIP-seq results with RNA-seq results, and we found that Purα can bind to 47 of them and cause a large number of changes (Fig. 4, Table S4). Therefore, it is believed that Purα can directly regulate these 47 genes, and the emergence of other differential genes may be affected by these 47 genes. Among the genes mentioned earlier in relation to AD pathogenesis and Aβ cleavage, only Insr is directly regulated by Purα. This means that Purα may rely on a deeper mechanism for the regulation of these genes.

## Discussion

Purα has long been considered as an indispensable factor in neurodevelopment. In experiments performed by Khalili^13^, Purα knockout mice developed severe tremors, spontaneous epilepsy and other neurological problems at 2 weeks of age and died 4 weeks after birth. However, the specific mechanism of Purα in the early development of neurons is still unclear. In a comparative study of cerebral cortex-suspended mice at different stages after birth by Gonzalez-Lozano^16^, total expression of brain proteins decreased after birth. By comparing with mouse large protein expression profile, we found that 67 genes may be regulated by Purα. These genes are involved in the regulation of neuronal metabolism, the formation of synapses, and the establishment of projections between neurons. This indicates that the loss of Purα has a significant impact on the formation of neuronal synapses and the establishment of the connection network between neurons, so this may be the cause of premature death of Purα-KO mice.

In the past few years, we have been exploring the relationship between Purα and neurodegenerative diseases, especially Alzheimer's disease (AD)^15^. In previous studies, we noticed that Purα regulates the rejuvenation of APP proteins. In this study, although we did not find direct evidence that Purα regulates APP, we found that Purα regulates other genes related to AD. These findings further confirm the role of Purα in the progression of AD.

At first, we notice the changes in LPL polymorphisms in the LPL gene are thought to be associated with the risk of AD^17^. LPL is a key enzyme that regulates the hydrolysis of triglycerides. LPL deficiency or dysfunction can cause dyslipidemia, which may increase the risk of AD^18^. LPL binds to amyloid beta protein (Aβ) and promotes cell surface association and Aβ uptake in mouse primary astrocytes^19^ and BV2 microglia^20^. Studies after human brain death have shown that LPL is widely distributed throughout brain tissue. Compared with control groups, LPL in the dentate gyrus granule cells and CSF samples of the AD group are significantly reduced^21^. In our study, we found similar LPL changes in AD after knocking out Purα, suggesting that Purα can regulate LPL, which may be a potential mechanism for AD development.

The large accumulation of Tau protein is one of the characteristics of AD, and Mapt is the coding gene of tau^22^. A large number of studies have shown that there is a large accumulation of Tau protein in the brain tissue of AD patients. Therefore, hyperphosphorylation and deposition of Tau protein may be a cause of AD^23^. In our study we found that Tau expression was up-regulated after Purα knockout, implying a potential inhibitory effect of Purα on Tau. GAPDH is a key gene in sugar metabolism, but a large number of independent studies have shown that GAPDH has non-glycolytic activity and is involved in pathogenesis and death in neurodegenerative diseases, such as Alzheimer's disease and Parkinson's disease^24^. GAPDH is often present in the AD temporal cortex along with phosphorylated Tau and Aβ peptide^25^. Since GAPDH has a region that binds to Aβ, some scholars believe that the aggregate of GAPDH provides seeds for the specificity of Aβ^26^. Studies show that nitrosated GAPDH can enhance the degree of acetylation of Tau; in the presence of Aβ, it can promote the aggregation of Tau into neurofibrillary tangles^27^. GAPDH and Tau appear to play highly intricate roles in the regulation of AD, so mRNA expression may not provide an adequate explanation for this phenomenon. At the same time, in our study, Tau was up-regulated and GAPDH was down-regulated. In combination with Chip-seq, we issued direct evidence that Purα binds to Mapt, which shows that Purα can directly bind to and regulate the expression of Tau. This is evidence that Purα may be directly involved in the occurrence of AD.

Decreased Aβ clearance is one of the main features of AD. In this study, we identified 5 genes involved in clearance of Aβ, as Mme, C3, Lrp1, Insr and Trem2, which may be affected by Purα. Coincidentally, the 5 genes were all down-regulated. The NEP protein encoded by the membrane metallo-endopeptidase (Mme) gene is one of the major contributors to brain Aβ clearance and is directly involved in the degradation of Aβ^28^. Studies have shown that NEP inhibitors can cause biochemical and pathological deposition of Aβ_1–42_^29^, and in vitro experiments show that NEP can rapidly degrade Aβ_1–40_ and Aβ_1–42_^30^, while exogenous supplementation of NEP can reduce the deposition of Aβ in AD transgenic mice^31^. In our study, knocking out Purɑ resulted in a decrease in Mme expression, suggesting that the expression of NEP was dependent on Purα. Although the mechanism of this regulation is unclear, and ChIP-seq studies did not show evidence of Purα regulation of Mme, current research can still provide some insight. According to related studies, HIV-1 transactivator (Tat) can reduce the expression and activity of NEP, thereby increasing the deposition of Aβ, which is considered to be an important cause of HIV-related cognitive impairment^32^. At the same time, other studies have demonstrated the close relationship between Purα and Tat. Purα promotes translation of HIV in vivo by binding to HIV-1 Tat and TAR RNA^33^. From the above studies we noticed that Tat can bind to Purα, and this combination may have a similar repressive effect on Purα, causing Purα to fail to exert its normal physiological effects. The Purα knockout will result in a decrease in Mme, so the reduction in HIV-related NEP may be due to this relationship.

LRP1 is a member of the low-density lipoprotein receptor family^34^, which has four extracellular ligand binding domains that bind to different ligands, including APP^35^, Apolipoprotein E(ApoE)^36^, and α2 macroglobulin (α2M)^34^. LRP1 can be combined with APP before it is cut by furin^37^, which slows APP movement^38^, and promotes further processing of the protein^39^. LRP1 binds to APP to facilitate processing of APP, but this effect appears to increase the production of Aβ^40^. Although LRP1 caused the production of Aβ, we cannot ignore the fact that it promotes Aβ transport. LRP1 can directly bind to Aβ through the LRP1 N-terminal domain or by binding ApoE or α2M^41^. LRP1 can transport Aβ to the blood–brain barrier by binding to Aβ and releasing Aβ into the blood, which is the main evidence that LRP1 is involved in Aβ clearance^42–44^. In our study, Lrp1 decreased after Purα knockout, thus indicating that the expression of LRP1 requires the participation of Purα, indicating that Purα plays an important role in the processing of APP and the clearance of Aβ. Ndufa3 and mt-Co3 are mitochondria-associated proteins, which are reported relatively less in AD and appear to be associated with mitochondrial dysfunction in AD^45^.

Insulin receptor substrates have multiple functions, including enzyme binding activity, insulin binding activity, and binding activity of insulin receptor substrates^46^. There are few studies on the involvement of Insr in Aβ regulation. In a 2009 study^47^, it was shown that cells with normal Insr have the ability to reduce the reduction of Aβ oligomers to Aβ monomers, while the Insr mutation causes a loss of this ability, leading to the aggregation of Aβ oligomers. This increase suggests that Insr has the ability to participate in Aβ clearance.

Microglia are the main physical immune cells of the brain. When the brain is injured, microglia can migrate towards the injured site, which is considered to be the brain's first line of defense against physical damage^48,49^. Microglia can move under the influence of neurons. The damage caused chemotaxis of microglia and the interaction with neurons are mainly related to the P2Y12 receptor^48,49^. In this study, we noticed from the Chip-Seq analysis that Purα can bind to purinergic receptor P2Y12 DNA, but we did not find changes in mRNA, because we did not capture purinergic receptor P2Y12 mRNA from the two sets of data, which means that purinergic receptor P2Y12 is not expressed or rarely expressed in neurons. This suggests that Purα may play an important role in microglia, but we noticed the other two genes Complement receptor 3 (C3) and TREM2. Usually these two genes are thought to mediate the Aβ clearance activity of microglia ^50,51^. And we also found changes in C3 and TREM2 in neurons, which means that they may be involved in Aβ clearance in neurons.

C3 plays a central role in the activation of the complement system and participates in the human immune response^50^. In the brains of Alzheimer's patients, complement components were detected in the amyloid core of senile plaques^52^, and an increase in CR3 was found in microglia^53^. C3 can be cleaved by C3 convertase to form C3b. On the one hand, C3b can bind to Aβ to form an Aβ-C3b complex, and can bind to CR3 and activate microglia to phagocytose Aβ, thereby promoting Aβ clearance^54^. In another study^55^, the ability of C3-deficient N9 microglia to phagocytose fibrillar Aβ was significantly reduced, further confirming that activation of the complement C3 system is an important factor in the phagocytosis of Aβ by microglia. In our study, the decrease in Purα caused a decrease in C3, indicating that the synthesis of C3 was dependent on the presence of Purα. Therefore, while our study is based on neuronal cells, the reduction of Purα may also affect the ability of microglia.

The protein encoded by TREM2 is part of the immunoglobulin and lectin-like superfamily and is part of the innate immune system. TREM2 is a surface receptor required for microglia to respond to neurodegeneration, including proliferation, survival, aggregation and phagocytosis. TREM2 mutations cause autophagy in microglia. Increasing cyclocreatine in the diet to supplement energy can reduce autophagy of microglia and reduce Aβ deposition in TREM2-deficient mice, suggesting that TREM2 affects Aβ clearance in microglia by affecting cell energy metabolism^56^. In addition, Aβ_42_ deposition in age-related macular degeneration also appears to be associated with a deficiency in TREM2^51^; the TREM2 R47H variant also shows reduced TREM2 mRNA expression and increases the risk of AD development^57^. Studies have shown that TREM2 is involved in the formation of AD, and in our study, there was a significant decrease in Trem2 after Purα knockout, indicating that Purα may be involved in the important process of AD.

We found some genes that may interact with APP and PSEN from the above 47 genes, based on string protein interaction analysis, such as Vldlr, Igfbp7, Kng2, Pros1, and Jag1. Among these results, APP, Kng2, and Igfbp7 were simultaneously regulated by phosphorylation of Fam20C enzyme^58^, and there was a weak co-expression relationship between APP and Igfbp7 (co-expression score = 0.057).

Vldlr belongs to the low-density lipoprotein receptor family and binds to ApoE, which is essential for Reelin pathway activation^59^. Activation of the Reelin pathway increases NMDA receptor activity by promoting tyrosine phosphorylation of the NR2 subunit, which is important in enhancing glutamatergic neurotransmission^60–63^. In addition, Reelin is involved in the transport and processing of APP, and is able to interact with Aβ oligomers to antagonize its negative effects on synaptic function^64–66^. In this study, we found that Purα binds directly to Vldlr DNA and positively regulates it, which means that Purα enhances Reelin activity by promoting Vldlr expression. Pros1 is a ligand for Mer tyrosine kinase (MerTK) and activation of MerTK is considered to be an important factor in amyloid-stimulated phagocytosis^67^. A decrease in Pros1 means that the likelihood of activation of MerTK is diminished, which in turn may affect the phagocytosis of Aβ. Jag1 is a substrate for BACE1 (β-secretase 1) and can be cleaved by BACE1^68^. At the same time, Jag1 is a ligand of Notch that promotes the activation of Notch. Loss of BACE1 cleavage causes an increase in Jag1, which enhances the transmission of Notch signaling. This is thought to be a possible mechanism by which BACE1 is involved in the balance of neurogenesis and astrogenesis^68^. In this study, Purα was able to directly regulate Jag1, and the lack of Purα caused up-regulation of Jag1, indicating that Purα can participate in the regulation of BACE1 on neurons and astrocytes.

## Conclusions

In this study, we found a potential mechanism for Purα in neuronal development and maintenance of normal function, raising awareness of Purα. Purα’s regulatory role in AD was unforeseen. Our research confirms that Purα can participate in the pathogenesis of AD by directly regulating the Tau and indirectly regulating Aβ clearance and the regulation of AD-related genes. Of course, the occurrence of AD is a complicated process. Purα plays a vital role in the occurrence of AD, participating in important aspects of pathogenesis.

## Supplementary Information

Supplementary Information.

## Data Availability

RNA-seq and ChIP-seq data have been uploaded to the Sequence Read Archive (SRA): https://www.ncbi.nlm.nih.gov/sra. RNA-seq data: SRA:SUB6809189; ChIP. data: SRA:SUB6906483.

## Abbreviations

AD: Alzheimer’s disease
Purα: Purine rich element binding protein A
HT22: Hippocampal neuronal cell line, HT22
GO: Gene ontology
KEGG: Kyoto encyclopedia of genes and genomes
α2M: α2 Macroglobulin
ApoE: Apolipoprotein E
MerTK: Mer tyrosine kinase
BACE1: β-Secretase 1
