# Supplementary material for: Analysis of the role of Purα in the pathogenesis of Alzheimer's disease based on RNA-seq and ChIP-seq
Source: Sci Rep. 2021 Jun 9;11:12178. doi: 10.1038/s41598-021-90982-1 (PMC8190037; doi:10.1038/s41598-021-90982-1)
Supplement: Supplementary file 1 — Supplementary Information. [file 41598_2021_90982_MOESM1_ESM.pdf]

S1 Differentially expressed genes					
#ID	preferredName	FDR_edgeR	log2FC_edg	Fold_differe	regulated
ENSMUSG00000118365	ENSMUSG00000118365	5.797E-05	-1.2327916	1.67394889	down
ENSMUSG00000118097	ENSMUSG00000118097	3.23E-16	-2.7616822	3.91502463	down
ENSMUSG00000118032	ENSMUSG00000118032	2.736E-06	7.79527218	2.92222222	up
ENSMUSG00000117278	ENSMUSG00000117278	2.876E-06	2.40082335	1.9513382	up
ENSMUSG00000117192	ENSMUSG00000117192	2.085E-05	-7.611195	2.69411765	down
ENSMUSG00000117050	ENSMUSG00000117050	2.611E-07	-2.7874242	2.48307692	down
ENSMUSG00000116508	ENSMUSG00000116508	2.207E-05	-1.7700952	1.74651811	down
ENSMUSG00000114934	ENSMUSG00000114934	0.0002263	-1.2001818	1.57107723	down
ENSMUSG00000114835	ENSMUSG00000114835	2.466E-06	-4.9152023	2.96521739	down
ENSMUSG00000114515	ENSMUSG00000114515	7.087E-06	-1.9730978	1.91826923	down
ENSMUSG00000114436	ENSMUSG00000114436	0.0002037	-1.0684386	1.62777101	down
ENSMUSG00000114433	ENSMUSG00000114433	1.284E-14	-1.9008312	3.1545271	down
ENSMUSG00000113425	ENSMUSG00000113425	0.0001269	3.69007558	1.74172185	up
ENSMUSG00000113061	ENSMUSG00000113061	0.001235	0.83945327	1.58134276	up
ENSMUSG00000111681	ENSMUSG00000111681	0.0003904	-2.0268621	1.51804124	down
ENSMUSG00000111394	ENSMUSG00000111394	2.189E-05	-1.1324917	1.83314575	down
ENSMUSG00000110790	ENSMUSG00000110790	4.253E-06	2.43166612	1.92802057	up
ENSMUSG00000110537	ENSMUSG00000110537	8.6E-05	2.85820417	1.67982456	up
ENSMUSG00000110185	ENSMUSG00000110185	8.539E-07	-1.5732145	1.97881356	down
ENSMUSG00000109925	ENSMUSG00000109925	8.548E-06	2.69875945	1.94333333	up
ENSMUSG00000109881	ENSMUSG00000109881	7.37E-19	-2.0677215	3.8574756	down
ENSMUSG00000109724	ENSMUSG00000109724	5.697E-21	3.4265589	5.29	up
ENSMUSG00000108137	ENSMUSG00000108137	0.000444	-3.5998225	1.6971831	down
ENSMUSG00000107997	ENSMUSG00000107997	0.0009939	-4.1962251	1.57391304	down
ENSMUSG00000107969	ENSMUSG00000107969	0.0002763	-1.4972143	1.51001178	down
ENSMUSG00000107741	ENSMUSG00000107741	0.0001798	-2.8542797	1.7627907	down
ENSMUSG00000107191	ENSMUSG00000107191	0.0016709	0.83145722	1.52430635	up
ENSMUSG00000106933	ENSMUSG00000106933	4.756E-06	-1.3246504	1.8698946	down
ENSMUSG00000106383	ENSMUSG00000106383	0.0001798	-2.8542797	1.7627907	down
ENSMUSG00000105366	ENSMUSG00000105366	6.734E-05	1.95525282	1.57640232	up
ENSMUSG00000105302	ENSMUSG00000105302	3.484E-07	-8.0243866	3.85882353	down
ENSMUSG00000104671	ENSMUSG00000104671	0.000444	3.52735601	1.50993377	up
ENSMUSG00000103309	ENSMUSG00000103309	9.138E-07	-1.3008623	2.02195333	down
ENSMUSG00000103270	ENSMUSG00000103270	1.353E-05	-1.6913094	1.78555305	down
ENSMUSG00000103082	ENSMUSG00000103082	6.151E-05	1.70026195	1.54520918	up
ENSMUSG00000103034	ENSMUSG00000103034	0.0001752	-1.4203839	1.54868914	down
ENSMUSG00000102929	ENSMUSG00000102929	0.0003945	-4.3329242	1.7826087	down
ENSMUSG00000102918	ENSMUSG00000102918	0.0003244	-0.9531882	1.68730131	down
ENSMUSG00000102311	ENSMUSG00000102311	0.0003003	3.58365989	1.58940397	up
ENSMUSG00000102224	ENSMUSG00000102224	2.704E-11	-1.5887839	2.736814	down
ENSMUSG00000101859	ENSMUSG00000101859	0.0002599	-1.1636521	1.5576225	down
ENSMUSG00000101355	Hist1h3h	0.000444	3.52735601	1.50993377	up
ENSMUSG00000101162	ENSMUSG00000101162	0.0007677	-0.9829489	1.53248621	down
ENSMUSG00000101111	ENSMUSG00000101111	0.0030636	-0.788544	1.59502584	down
ENSMUSG00000100131	ENSMUSG00000100131	5.217E-07	-1.3247723	2.05383132	down
ENSMUSG00000099632	ENSMUSG00000099632	0.0002263	2.60097179	1.51383399	up

ENSMUSG00000099583	Hist1h3d	3.965E-05	3.4122911	1.87640449	up
ENSMUSG00000097993	ENSMUSG00000097993	5.113E-09	2.09994029	2.31130064	up
ENSMUSG00000097960	ENSMUSG00000097960	3.965E-05	-2.494758	1.85197368	down
ENSMUSG00000097903	ENSMUSG00000097903	2.293E-06	-2.2345713	2.07786885	down
ENSMUSG00000097893	ENSMUSG00000097893	0.0004135	-2.9523928	1.64583333	down
ENSMUSG00000097418	ENSMUSG00000097418	0.0003945	-4.3329242	1.7826087	down
ENSMUSG00000097216	ENSMUSG00000097216	1.417E-08	-1.5868331	2.2482663	down
ENSMUSG00000097204	ENSMUSG00000097204	0.0009593	-0.9467492	1.53182092	down
ENSMUSG00000097081	ENSMUSG00000097081	2.26E-05	1.2022905	1.67083855	up
ENSMUSG00000097078	Gm26566	0.0002002	-1.3303296	1.55239521	down
ENSMUSG00000096001	Gpr15l	1.165E-15	-2.5352091	3.62891344	down
ENSMUSG00000095653	Gm21818	4.026E-07	4.23821188	2.78145695	up
ENSMUSG00000095427	Rps2-ps6	0.0001289	1.14280393	1.53884869	up
ENSMUSG00000095180	Rhox5	2.718E-07	1.47433038	1.92824641	up
ENSMUSG00000092564	ENSMUSG00000092564	5.318E-05	1.57866631	1.58174524	up
ENSMUSG00000092365	ENSMUSG00000092365	4.262E-05	1.36227751	1.57577566	up
ENSMUSG00000092300	ENSMUSG00000092300	5.892E-05	2.89555601	1.73245614	up
ENSMUSG00000092220	ENSMUSG00000092220	4.261E-10	-1.729372	2.47508185	down
ENSMUSG00000092203	ENSMUSG00000092203	9.005E-05	1.02068247	1.67434263	up
ENSMUSG00000091479	ENSMUSG00000091479	5.697E-21	-2.2292997	4.18459447	down
ENSMUSG00000091421	ENSMUSG00000091421	0.000157	-1.2109369	1.58731707	down
ENSMUSG00000091412	ENSMUSG00000091412	7.006E-19	-2.8363322	4.37539103	down
ENSMUSG00000091337	Eid1	0.005845	-0.7364614	1.56782092	down
ENSMUSG00000091119	Ccdc152	9.24E-07	-3.8232209	2.79761905	down
ENSMUSG00000090863	ENSMUSG00000090863	0.0001009	-1.1460937	1.66261028	down
ENSMUSG00000090854	Gm4340	6.075E-05	3.09373638	1.75980392	up
ENSMUSG00000090799	Klhl33	5.318E-05	-3.8616756	2.13380282	down
ENSMUSG00000090733	Rps27	0.0030111	0.78292065	1.52892543	up
ENSMUSG00000090235	ENSMUSG00000090235	0.0004135	-2.9523928	1.64583333	down
ENSMUSG00000090208	ENSMUSG00000090208	8.125E-07	-1.8119273	2.00531915	down
ENSMUSG00000090145	Ugt1a6b	1.151E-08	-2.7679914	2.75773196	down
ENSMUSG00000089961	ENSMUSG00000089961	7.38E-17	-2.3836733	3.66628702	down
ENSMUSG00000089698	ENSMUSG00000089698	8.967E-05	-3.1268258	1.90625	down
ENSMUSG00000087700	ENSMUSG00000087700	2.706E-08	-1.7349192	2.22888617	down
ENSMUSG00000087400	ENSMUSG00000087400	3.012E-09	1.83190325	2.22940818	up
ENSMUSG00000087370	Tmem170b	0.0002793	-1.2694733	1.53637566	down
ENSMUSG00000087141	Plcxd2	0.0002036	-1.3900988	1.53832442	down
ENSMUSG00000087095	ENSMUSG00000087095	0.0002222	-1.5463481	1.52711223	down
ENSMUSG00000086930	ENSMUSG00000086930	0.0003686	-2.3108176	1.58881579	down
ENSMUSG00000086815	ENSMUSG00000086815	3.965E-05	-2.494758	1.85197368	down
ENSMUSG00000086725	ENSMUSG00000086725	0.0003167	-1.6181486	1.52857143	down
ENSMUSG00000086717	ENSMUSG00000086717	0.0003167	-1.6181486	1.52857143	down
ENSMUSG00000086514	ENSMUSG00000086514	4.534E-05	-1.4389243	1.67384615	down
ENSMUSG00000085791	ENSMUSG00000085791	3.07E-09	4.60757429	3.77483444	up
ENSMUSG00000085754	ENSMUSG00000085754	0.0002242	-2.0811834	1.58762887	down
ENSMUSG00000085711	ENSMUSG00000085711	1.075E-06	-2.4482726	2.21323529	down
ENSMUSG00000085505	ENSMUSG00000085505	1.511E-11	-1.7825792	2.68673012	down
ENSMUSG00000085360	ENSMUSG00000085360	2.736E-06	-7.8325329	3.27058824	down

ENSMUSG00000085241	ENSMUSG00000085241	7.39E-05	1.11194647	1.61050463	up
ENSMUSG00000085178	ENSMUSG00000085178	4.571E-05	-1.4872002	1.66666667	down
ENSMUSG00000084817	ENSMUSG00000084817	0.0002113	-1.808832	1.57586837	down
ENSMUSG00000083773	ENSMUSG00000083773	3.303E-05	-1.0289869	1.99356875	down
ENSMUSG00000082998	ENSMUSG00000082998	1.779E-05	2.17575465	1.73788546	up
ENSMUSG00000082450	ENSMUSG00000082450	1.966E-08	2.23592472	2.28815977	up
ENSMUSG00000082424	ENSMUSG00000082424	4.951E-14	-2.0777425	3.09492848	down
ENSMUSG00000082314	ENSMUSG00000082314	4.206E-13	-2.6092699	3.33704735	down
ENSMUSG00000082016	ENSMUSG00000082016	6.831E-08	-1.4419946	2.17045204	down
ENSMUSG00000081214	ENSMUSG00000081214	6.933E-05	1.16058113	1.59131653	up
ENSMUSG00000079685	Ulbp1	5.996E-08	-1.3346376	2.2683152	down
ENSMUSG00000079652	Fam71f2	2.827E-05	-3.2450701	2.109375	down
ENSMUSG00000079560	Hoxa3	1.915E-05	-2.4819385	1.93846154	down
ENSMUSG00000079387	Luzp4	4.095E-11	-4.8825163	5.00704225	down
ENSMUSG00000079343	C1s2	2.554E-05	-4.6791224	2.4173913	down
ENSMUSG00000079018	Ly6c1	0.0001484	-1.4655304	1.57551826	down
ENSMUSG00000078942	Naip6	0.0001951	-3.042244	1.77604167	down
ENSMUSG00000078812	Eif5a	0.0024654	0.78572773	1.62814985	up
ENSMUSG00000078771	Evi2a	3.872E-05	-2.8527337	1.97478992	down
ENSMUSG00000078768	Zfp566	5.449E-05	1.7239764	1.59264126	up
ENSMUSG00000078651	Aoc2	0.0001183	1.54453906	1.50746269	up
ENSMUSG00000078636	ENSMUSG00000078636	1.007E-05	-1.0895144	2.05312891	down
ENSMUSG00000078566	Bnip3	1.304E-19	-2.09368	4.04147671	down
ENSMUSG00000078497	Gm13145	2.951E-06	1.95749847	1.81938326	up
ENSMUSG00000078300	ENSMUSG00000078300	1.214E-05	-1.8126877	1.80501393	down
ENSMUSG00000077222	ENSMUSG00000077222	1.295E-05	7.63255261	2.53333333	up
ENSMUSG00000076454	ENSMUSG00000076454	4.948E-05	1.10017863	1.66077146	up
ENSMUSG00000075324	Fign	5.922E-05	-1.4892527	1.6427256	down
ENSMUSG00000075254	Heg1	0.0034262	-0.7982603	1.53327444	down
ENSMUSG00000074934	Grem1	1.34E-08	-1.3848943	2.35323933	down
ENSMUSG00000074813	ENSMUSG00000074813	2.445E-05	1.33545161	1.62181996	up
ENSMUSG00000074794	Arrdc3	2.71E-05	-1.1994867	1.75617198	down
ENSMUSG00000074749	Kiz	0.0001965	-1.0508695	1.6370673	down
ENSMUSG00000074604	Mgst2	4.43E-05	-1.1605634	1.73198483	down
ENSMUSG00000074398	ENSMUSG00000074398	2.732E-05	-1.2784638	1.72934828	down
ENSMUSG00000074305	Peak1	0.0054353	-0.7502327	1.53734887	down
ENSMUSG00000073652	ENSMUSG00000073652	1.193E-05	-1.366511	1.78390244	down
ENSMUSG00000073198	Bnip3l-ps	0.0001872	-1.1958652	1.57581574	down
ENSMUSG00000073177	Gm773	1.463E-16	-2.2008284	3.49011713	down
ENSMUSG00000072941	Sod3	9.128E-11	-1.6297144	2.60085444	down
ENSMUSG00000072768	ENSMUSG00000072768	1.297E-10	-8.6075009	6.30588235	down
ENSMUSG00000072623	Zfp9	0.0002847	-1.0660866	1.59013653	down
ENSMUSG00000072620	Slfn2	2.119E-05	-1.7306375	1.74580645	down
ENSMUSG00000071793	ENSMUSG00000071793	0.0003244	-1.2309806	1.53265428	down
ENSMUSG00000071658	Gng3	8.967E-05	3.05323886	1.69607843	up
ENSMUSG00000071552	Tigit	3.927E-07	2.09463377	1.99697428	up
ENSMUSG00000071547	Nt5dc2	0.0046884	-0.7497357	1.59839555	down
ENSMUSG00000071052	Rpl7a-ps5	7.923E-05	1.28651458	1.53994778	up

ENSMUSG00000071042	Rasgrp3	4.081E-05	1.49927017	1.58989229	up
ENSMUSG00000070867	Trabd2b	9.976E-08	-3.6683773	3	down
ENSMUSG00000070348	Ccnd1	0.0023921	0.79689776	1.55221029	up
ENSMUSG00000070319	Eif3g	0.0004037	0.90266249	1.66405791	up
ENSMUSG00000070034	Sp110	0.0003053	-3.2569284	1.72619048	down
ENSMUSG00000070031	Sp140	8.092E-05	-1.2041862	1.6546169	down
ENSMUSG00000068113	Gm4907	1.632E-09	2.66747497	2.63578947	up
ENSMUSG00000067629	Syngap1	3.066E-05	-1.2477578	1.73481266	down
ENSMUSG00000067288	Rps28	0.0004738	0.88743089	1.68323801	up
ENSMUSG00000067085	Mxi1	1.979E-07	-1.6643721	2.08403361	down
ENSMUSG00000066026	Dhrs3	4.103E-18	-2.4155568	3.8284085	down
ENSMUSG00000064373	Sepp1	9.183E-11	-2.7657621	3.15368852	down
ENSMUSG00000064358	mt-Co3	0.0012664	-0.8651924	1.60329755	down
ENSMUSG00000064330	Pde6h	3.447E-08	-1.446479	2.21503293	down
ENSMUSG00000064264	Zfp428	5.31E-05	1.1001816	1.65533981	up
ENSMUSG00000064246	Chil1	6.977E-42	-7.9901836	33.7478261	down
ENSMUSG00000063888	Rpl7l1	2.097E-06	1.16065311	2.00703766	up
ENSMUSG00000063802	Hspbp1	0.001207	0.85253384	1.54242395	up
ENSMUSG00000063632	Sox11	1.539E-12	2.21199237	2.79788039	up
ENSMUSG00000063382	Bcl9l	0.0078922	-0.7167393	1.53097207	down
ENSMUSG00000063229	Ldha	0.0013382	-0.8217251	1.78134237	down
ENSMUSG00000063193	Cd300lb	9.1E-05	-1.4803467	1.6042677	down
ENSMUSG00000063011	Msln	0.0001278	0.97132097	1.72470393	up
ENSMUSG00000062901	Klhl24	5.988E-10	-1.7015127	2.4557777	down
ENSMUSG00000062554	ENSMUSG00000062554	1.491E-22	-2.7909802	4.81661342	down
ENSMUSG00000062515	Fabp4	9.957E-05	-4.516383	2.09565217	down
ENSMUSG00000062380	Tubb3	3.996E-05	1.08936003	1.69031709	up
ENSMUSG00000062300	Pvrl2	0.000444	-0.9103457	1.71106427	down
ENSMUSG00000062070	Pgk1	7.27E-05	-0.9845711	1.9835388	down
ENSMUSG00000061878	Sphk1	0.0016112	-0.8344257	1.62154777	down
ENSMUSG00000061815	Rufy4	0.0001122	-2.7487066	1.81092437	down
ENSMUSG00000061458	Nol10	1.731E-05	1.16488906	1.71749108	up
ENSMUSG00000061360	Phf5a	0.0022767	0.80975038	1.51436691	up
ENSMUSG00000061353	Cxcl12	5.438E-08	-1.2995349	2.39441366	down
ENSMUSG00000061024	Rrs1	0.0014923	0.8424687	1.52026234	up
ENSMUSG00000060961	Slc4a4	1.247E-07	-1.6314621	2.11719606	down
ENSMUSG00000060771	Tsga10	0.0001648	-1.7041226	1.59516616	down
ENSMUSG00000060639	Hist1h4i	0.0005464	0.95479456	1.50764873	up
ENSMUSG00000060459	Kng2	8.182E-06	-2.4174239	2.01089918	down
ENSMUSG00000060224	Pyroxd2	1.968E-06	-1.4341107	1.90864086	down
ENSMUSG00000060131	Atp8b4	2.628E-06	-1.4815913	1.9016129	down
ENSMUSG00000059456	Ptk2b	2.736E-06	-7.8325329	3.27058824	down
ENSMUSG00000059159	ENSMUSG00000059159	1.972E-05	1.41227376	1.64167651	up
ENSMUSG00000058656	Samd12	0.0002263	-2.6749395	1.70168067	down
ENSMUSG00000058420	Syt17	1.74E-06	-1.3806604	1.93522961	down
ENSMUSG00000057863	Rpl36	0.0040376	0.75443936	1.55499973	up
ENSMUSG00000057666	Gapdh	0.0028009	-0.7847158	1.64966893	down
ENSMUSG00000057329	Bcl2	8.357E-06	1.11881408	1.86250968	up

ENSMUSG00000057322	Rpl38	0.0009132	0.85280451	1.62504704	up
ENSMUSG00000057193	Slc44a2	0.0077813	-0.7189596	1.52826427	down
ENSMUSG00000057000	Nxf3	2.085E-05	-7.611195	2.69411765	down
ENSMUSG00000056749	Nfil3	0.0007945	-0.904632	1.61586133	down
ENSMUSG00000056536	Pign	0.0046513	-0.7784414	1.5044089	down
ENSMUSG00000056270	Prr9	9.954E-07	-7.9316466	3.56470588	down
ENSMUSG00000056209	Npm3	0.0001441	0.97250767	1.69313821	up
ENSMUSG00000056043	Rgs9bp	0.0002932	-1.8963228	1.54700855	down
ENSMUSG00000055782	Abcd2	1.547E-09	-2.473152	2.75384615	down
ENSMUSG00000055675	Kbtbd11	0.0001694	-2.4782428	1.71276596	down
ENSMUSG00000055493	Epm2a	1.843E-07	-2.8944348	2.5625	down
ENSMUSG00000055322	Tns1	1.986E-05	-1.1192283	1.85657325	down
ENSMUSG00000055172	C1ra	2.294E-07	-1.3226757	2.13282499	down
ENSMUSG00000055116	Arntl	0.0001213	-1.3035664	1.61077111	down
ENSMUSG00000055030	Sprr2e	8.798E-06	-3.3543529	2.3125	down
ENSMUSG00000054793	Cadm4	6.731E-05	1.34258415	1.54301403	up
ENSMUSG00000054626	Xlr	2.171E-05	-1.12392	1.84017972	down
ENSMUSG00000054568	Usp17la	0.0001033	1.95482418	1.53830645	up
ENSMUSG00000054293	P2ry10b	8.555E-09	-2.1486235	2.41419355	down
ENSMUSG00000054263	Lifr	3.419E-07	-1.3805414	2.05901563	down
ENSMUSG00000053801	Grwd1	0.0002778	0.93812084	1.64377722	up
ENSMUSG00000053746	Ptrh1	4.43E-05	1.07159731	1.70490196	up
ENSMUSG00000053702	Nebl	7.429E-06	1.85874626	1.77003942	up
ENSMUSG00000053613	ENSMUSG00000053613	0.000455	-1.2140463	1.50030921	down
ENSMUSG00000053470	Kdm3a	0.0001466	-1.0104911	1.72604807	down
ENSMUSG00000053332	ENSMUSG00000053332	0.0016825	0.82390448	1.54708274	up
ENSMUSG00000052957	Gas1	9.921E-11	-1.5310529	2.70619613	down
ENSMUSG00000052477	ENSMUSG00000052477	0.0001545	-1.3244298	1.58044383	down
ENSMUSG00000052353	Cemip	4.261E-10	-1.6780897	2.46930342	down
ENSMUSG00000052331	Ankrd44	1.74E-05	-1.3471489	1.75454993	down
ENSMUSG00000052143	Gm9869	2.092E-05	-2.5512049	1.9375	down
ENSMUSG00000052085	Dock8	9.9E-05	2.56051802	1.60869565	up
ENSMUSG00000051627	Hist1h1e	0.0001269	-3.762562	1.95774648	down
ENSMUSG00000051457	Spn	0.0001176	1.39731007	1.50614439	up
ENSMUSG00000051048	P4ha3	0.0005609	-0.9347446	1.62233055	down
ENSMUSG00000051037	Zfp455	0.0007531	-2.4184649	1.50191571	down
ENSMUSG00000050965	Prkca	0.0001836	-0.9866227	1.72426938	down
ENSMUSG00000050953	Gja1	7.14E-05	-1.0087201	1.84833544	down
ENSMUSG00000050914	Ankrd37	1.293E-14	-2.0961322	3.17924909	down
ENSMUSG00000050737	Ptges	3.738E-05	-1.5204759	1.68232044	down
ENSMUSG00000050671	Ism2	3.477E-05	3.83628831	1.97350993	up
ENSMUSG00000050359	Sprr1a	6.008E-07	-1.3519095	2.02762803	down
ENSMUSG00000050332	Amer1	2.064E-05	-1.2618023	1.76872399	down
ENSMUSG00000049694	BC048671	1.295E-05	7.63255261	2.53333333	up
ENSMUSG00000049555	Tmie	0.0002735	-1.3470348	1.51969824	down
ENSMUSG00000049097	Ankrd34a	0.0001954	-1.1785673	1.57972309	down
ENSMUSG00000049092	Gpr137c	0.0003686	-2.3108176	1.58881579	down
ENSMUSG00000048826	Dact2	0.0005478	-1.0911295	1.51231328	down

ENSMUSG00000048756	Foxo3	0.0001574	-0.978341	1.76278592	down
ENSMUSG00000048706	Lurap1l	3.363E-06	1.22044583	1.83215816	up
ENSMUSG00000048562	Sp8	5.318E-05	3.78917821	1.90066225	up
ENSMUSG00000048347	Pcdhb18	2.931E-06	-2.7993638	2.23404255	down
ENSMUSG00000048126	Col6a3	1.673E-10	-1.793377	2.54268293	down
ENSMUSG00000047996	Prrg1	1.558E-06	-1.3438343	1.94824191	down
ENSMUSG00000047822	Gm6484	4.765E-08	-8.1936958	4.45882353	down
ENSMUSG00000047749	Zc3hav1l	3.306E-05	-1.3291096	1.71741344	down
ENSMUSG00000047747	Rnf150	1.452E-13	-1.9595666	3.00468165	down
ENSMUSG00000047632	Fgfbp3	4.202E-05	1.25384538	1.60389105	up
ENSMUSG00000047562	Mmp10	0.0002125	-1.5870993	1.52917232	down
ENSMUSG00000047250	Ptgs1	0.0002793	-0.930161	1.77389607	down
ENSMUSG00000047153	Khnyn	0.000333	-0.9670761	1.65794745	down
ENSMUSG00000047139	Cd24a	0.0078922	-0.7186891	1.52228031	down
ENSMUSG00000046711	Hmga1	0.0032068	0.77858967	1.5221736	up
ENSMUSG00000045790	Ccdc149	1.007E-07	-3.2833821	2.81512605	down
ENSMUSG00000045667	Smtnl2	6.336E-11	-2.0958985	2.7078926	down
ENSMUSG00000045629	Sh3tc2	1.915E-05	-1.5103281	1.74498797	down
ENSMUSG00000045518	Onecut3	2.337E-07	3.06374015	2.43478261	up
ENSMUSG00000045092	S1pr1	1.055E-05	-1.3887497	1.80654762	down
ENSMUSG00000044674	Fzd1	0.0010235	-0.8657387	1.64182393	down
ENSMUSG00000044548	Dact1	7.23E-08	-1.9677111	2.20993228	down
ENSMUSG00000044337	Ackr3	8.769E-06	-1.1511758	1.91861063	down
ENSMUSG00000043991	Pura	2.806E-22	-2.8361448	4.82932166	down
ENSMUSG00000043587	Pxylp1	1.817E-06	-1.2101342	2.03810797	down
ENSMUSG00000043333	Rhbdl2	2.554E-05	-4.6791224	2.4173913	down
ENSMUSG00000043067	Dpy19l1	1.242E-05	-1.0883368	1.99795334	down
ENSMUSG00000042988	Notum	0.0007425	-0.9451905	1.56721421	down
ENSMUSG00000042804	Gpr153	7.567E-05	-1.1241014	1.70195052	down
ENSMUSG00000042734	Ttc9	1.594E-05	-1.5012166	1.76251897	down
ENSMUSG00000042650	Alkbh5	0.0026414	-0.7943263	1.623789	down
ENSMUSG00000042599	Kdm7a	2.581E-05	-2.1371566	1.83035714	down
ENSMUSG00000042549	ENSMUSG00000042549	3.241E-05	1.61180103	1.62286861	up
ENSMUSG00000042501	Cpa6	0.0005319	-2.7291967	1.58604651	down
ENSMUSG00000042473	Tbc1d8b	0.0007243	-0.9864111	1.53761643	down
ENSMUSG00000042460	C1galt1	0.0012943	-0.9262942	1.51178918	down
ENSMUSG00000042444	Fam63b	0.0003679	-0.9897132	1.61884009	down
ENSMUSG00000042340	Ctf1	2.732E-05	1.28836202	1.63346774	up
ENSMUSG00000042198	Chchd7	0.0001901	1.0138244	1.58617049	up
ENSMUSG00000042109	Csdc2	0.0002688	1.04778038	1.51346417	up
ENSMUSG00000041974	Spidr	0.0049103	-0.7746639	1.50334957	down
ENSMUSG00000041841	Rpl37	0.0008543	0.85342721	1.65080196	up
ENSMUSG00000041797	Abca9	6.456E-05	-1.2968267	1.66226913	down
ENSMUSG00000041757	Plekha6	0.0001292	-1.435664	1.57642726	down
ENSMUSG00000041695	Kcnj2	2.126E-18	-3.105578	4.64385692	down
ENSMUSG00000041642	Kif21b	0.0063882	-0.7365338	1.53261598	down
ENSMUSG00000041577	Prelp	1.047E-08	-1.5164944	2.28332202	down
ENSMUSG00000041556	Fbxo2	0.0001466	-0.9667183	1.81610894	down

ENSMUSG00000041506	Rrp9	0.00058	0.8976031	1.5852757	up
ENSMUSG00000041417	Pik3r1	0.0037449	-0.7946745	1.52124068	down
ENSMUSG00000041040	Fam117b	0.0005161	-0.9505993	1.614669	down
ENSMUSG00000040943	Tet2	9.325E-10	-2.0084611	2.50382979	down
ENSMUSG00000040888	Gfer	0.0003973	0.94149452	1.57335596	up
ENSMUSG00000040690	Col16a1	0.0033694	-0.7713724	1.63903548	down
ENSMUSG00000040675	Mthfd1l	0.0062586	-0.7333612	1.55426198	down
ENSMUSG00000040653	Ppp1r14c	6.21E-10	-1.7658734	2.44902482	down
ENSMUSG00000040624	Plekhg1	0.0003962	-1.8050206	1.50590551	down
ENSMUSG00000040570	Rundc3b	0.0001849	-1.9044775	1.59631148	down
ENSMUSG00000040473	Cfap69	1.308E-05	-1.1606177	1.86745782	down
ENSMUSG00000040447	Spns2	0.0001545	2.14753027	1.52956298	up
ENSMUSG00000040433	Zbtb38	0.0029436	-0.8209187	1.52789342	down
ENSMUSG00000040249	Lrp1	0.0060121	-0.728225	1.60180571	down
ENSMUSG00000040152	Thbs1	0.0041878	-0.7583545	1.60398129	down
ENSMUSG00000040128	Pnrc1	3.444E-05	-1.0691751	1.85243205	down
ENSMUSG00000040093	Bmf	3.404E-13	-2.7858214	3.54876033	down
ENSMUSG00000040026	Saa3	8.133E-56	-3.8908019	12.8489196	down
ENSMUSG00000039840	Epg5	1.581E-05	-1.319449	1.76679671	down
ENSMUSG00000039831	Arhgap29	0.0005932	-0.9164449	1.64356261	down
ENSMUSG00000039787	Cercam	0.0005149	-0.9045359	1.69466503	down
ENSMUSG00000039765	Cc2d2a	0.0005139	-1.1064402	1.50903614	down
ENSMUSG00000039704	Lmbrd2	0.0001292	-1.0472857	1.6966672	down
ENSMUSG00000039485	Tspyl4	8.616E-05	-1.3223949	1.6374092	down
ENSMUSG00000039481	Nrtn	0.0001346	1.19262957	1.518684	up
ENSMUSG00000039474	Wfs1	8.616E-05	-1.0172602	1.79725555	down
ENSMUSG00000039405	Prss23	0.0002599	0.96348176	1.6059608	up
ENSMUSG00000039316	Rftn1	3.543E-10	3.75779511	3.51754386	up
ENSMUSG00000039286	Fndc3b	0.0002322	-0.9728073	1.70720639	down
ENSMUSG00000039191	Rbpj	0.000363	-0.917747	1.74565009	down
ENSMUSG00000039126	Prune2	0.001855	-0.853365	1.55252374	down
ENSMUSG00000038963	Slco4a1	0.0001772	-1.3437932	1.5629742	down
ENSMUSG00000038843	Gcnt1	9.114E-05	-7.4196036	2.27058824	down
ENSMUSG00000038648	Creb3l2	0.0024377	-0.8415995	1.52818192	down
ENSMUSG00000038587	Akap12	1.181E-06	1.31106713	1.87031125	up
ENSMUSG00000038539	Atf5	0.0018116	0.8215586	1.53508835	up
ENSMUSG00000038521	C1s1	7.507E-21	-2.3063198	4.1486511	down
ENSMUSG00000038489	Polr2l	0.0007148	0.89585558	1.55111202	up
ENSMUSG00000038415	Foxq1	2.258E-06	-1.2927975	1.94360232	down
ENSMUSG00000038402	Foxf2	9.705E-05	-1.0436022	1.73604322	down
ENSMUSG00000038400	Pmepa1	0.0001502	1.04677767	1.57606663	up
ENSMUSG00000038393	Txnip	4.767E-06	-1.1707332	1.96714	down
ENSMUSG00000038371	Sbf2	0.003982	-0.798451	1.50337682	down
ENSMUSG00000038351	Sgsm2	0.0003733	-0.9353985	1.69303613	down
ENSMUSG00000038319	Kcnh2	2.739E-05	-1.0983273	1.84221658	down
ENSMUSG00000038146	Notch3	0.0003244	-0.993479	1.62999026	down
ENSMUSG00000038122	Tbc1d32	8.908E-05	-1.4047674	1.61660079	down
ENSMUSG00000038072	Galnt11	0.000858	-0.9349985	1.56175753	down

ENSMUSG00000038070	Cntln	0.0002642	-1.0643216	1.60394218	down
ENSMUSG00000038067	Csf3	3.43E-05	-7.5501152	2.55294118	down
ENSMUSG00000038065	Mturn	0.0001153	-1.1630829	1.64213464	down
ENSMUSG00000038039	Gcc2	0.0008835	-0.9284878	1.5647349	down
ENSMUSG00000037999	Arap2	3.1E-06	-1.1960769	1.98387424	down
ENSMUSG00000037818	Abhd18	1.713E-05	-1.5122649	1.75494071	down
ENSMUSG00000037624	Kcnk2	1.237E-07	-1.9087087	2.1606383	down
ENSMUSG00000037434	Slc30a1	7.27E-05	1.26604446	1.54615385	up
ENSMUSG00000037379	Spon2	8.776E-13	-1.7266298	2.91801887	down
ENSMUSG00000037275	Gemin5	0.0014425	0.83926769	1.5369716	up
ENSMUSG00000037239	Spred3	0.0003828	-1.0880712	1.55157895	down
ENSMUSG00000037206	Islr	3.43E-05	-7.5501152	2.55294118	down
ENSMUSG00000037012	Hk1	0.0006685	-0.8735653	1.73315415	down
ENSMUSG00000037010	Apln	2.168E-13	-1.7695929	3.00957836	down
ENSMUSG00000036334	Igsf10	6.848E-05	-1.3367414	1.65556901	down
ENSMUSG00000036285	Noa1	0.0003686	0.95449606	1.56251895	up
ENSMUSG00000036256	Igfbp7	5.153E-07	1.27628542	1.96684744	up
ENSMUSG00000036103	Colec12	4.402E-08	-1.3500847	2.2833018	down
ENSMUSG00000035984	Nme5	2.466E-06	4.84464774	2.63934426	up
ENSMUSG00000035783	Acta2	2.038E-05	1.05734619	1.8779185	up
ENSMUSG00000035759	Bbs10	2.893E-05	-2.5232575	1.89473684	down
ENSMUSG00000035674	Ndufa3	0.0010235	0.88611574	1.50439935	up
ENSMUSG00000035284	Vps13c	7.398E-06	-1.3019786	1.84445885	down
ENSMUSG00000035273	Hpse	0.0006583	-0.9007709	1.65397007	down
ENSMUSG00000035215	Lsm7	0.001564	0.84032642	1.51617798	up
ENSMUSG00000035158	Mitf	1.536E-09	-2.227091	2.56387097	down
ENSMUSG00000035109	Shc4	3.66E-05	-1.0660192	1.85015858	down
ENSMUSG00000035105	Egln3	8.91E-17	-1.983046	3.51533963	down
ENSMUSG00000035093	Secisbp2l	0.004208	-0.7858779	1.51329973	down
ENSMUSG00000035049	Rrp12	4.325E-05	1.08053339	1.69572914	up
ENSMUSG00000035027	Map2k2	0.0016832	0.81327351	1.59944745	up
ENSMUSG00000034932	Mrpl54	0.0011495	0.85707967	1.5401992	up
ENSMUSG00000034892	Rps29	1.353E-05	1.08549617	1.86694781	up
ENSMUSG00000034858	Fam214a	3.051E-07	-1.667908	2.05062413	down
ENSMUSG00000034793	G6pc3	7.319E-36	-10.72013	33.2235294	down
ENSMUSG00000034729	Mrps10	2.038E-05	1.0989882	1.76811241	up
ENSMUSG00000034614	Pik3ip1	0.0012301	-0.9483028	1.50457666	down
ENSMUSG00000034522	Zfp395	1.878E-05	-1.0772507	1.93989119	down
ENSMUSG00000034303	Ccdc15	9.976E-08	-3.6683773	3	down
ENSMUSG00000034255	Arhgap27	7.73E-07	-1.9615007	2.04884668	down
ENSMUSG00000033487	Fndc3a	1.047E-08	-1.4431684	2.31982323	down
ENSMUSG00000033420	Antxr1	0.0001965	-0.975506	1.73460591	down
ENSMUSG00000033327	Tnxb	0.0001691	-1.4878038	1.5606383	down
ENSMUSG00000033316	Galnt9	0.000153	-7.3496356	2.12941176	down
ENSMUSG00000033066	Gas7	2.611E-13	-2.1923687	3.07407407	down
ENSMUSG00000033032	Afap1l1	3.872E-06	-4.8709762	2.85217391	down
ENSMUSG00000032892	Rangrf	0.0001345	1.02430224	1.61452447	up
ENSMUSG00000032652	Crebl2	9.43E-05	-1.3349439	1.6250808	down

ENSMUSG00000032625	Thsd7a	0.0003872	-1.0304938	1.57821437	down
ENSMUSG00000032558	Nphp3	8.616E-05	-1.3192808	1.63870581	down
ENSMUSG00000032374	Plod2	4.235E-08	-1.332077	2.31146831	down
ENSMUSG00000032363	Adamts7	4.871E-14	-1.9050463	3.05834549	down
ENSMUSG00000032332	Col12a1	0.0013235	-0.8293425	1.7079934	down
ENSMUSG00000032114	Slc37a4	0.0006412	-0.9372138	1.60064154	down
ENSMUSG00000032064	Dixdc1	3.378E-06	-1.2271301	1.94342813	down
ENSMUSG00000032009	Sesn3	0.0001315	-0.9984812	1.76296067	down
ENSMUSG00000032000	Birc3	0.0003244	-1.3992903	1.50630456	down
ENSMUSG00000031987	Egln1	1.516E-08	-1.3538781	2.42735509	down
ENSMUSG00000031896	Ctrl	9.114E-05	7.38239798	2.03333333	up
ENSMUSG00000031722	Hp	2.027E-15	-9.1710869	9.97647059	down
ENSMUSG00000031647	Mfap3l	0.0002232	-2.2557172	1.60923077	down
ENSMUSG00000031644	Nek1	0.0002642	-1.069207	1.59769167	down
ENSMUSG00000031637	Lrp2bp	1.401E-06	-1.5181979	1.94523549	down
ENSMUSG00000031596	Slc7a2	6.248E-09	-1.4501945	2.36119734	down
ENSMUSG00000031506	Ptpn7	1.949E-05	1.81445132	1.68960864	up
ENSMUSG00000031486	Gpr124	0.0048607	-0.7622768	1.53474118	down
ENSMUSG00000031380	Figf	0.0029146	-0.8216473	1.52905368	down
ENSMUSG00000031284	Pak3	0.0009939	-0.8963428	1.59092369	down
ENSMUSG00000031283	Chrdl1	3.162E-08	-1.3931731	2.2597759	down
ENSMUSG00000031278	Acsl4	0.0010311	-0.8745411	1.61854982	down
ENSMUSG00000031274	Col4a5	0.0043488	-0.7642039	1.56236845	down
ENSMUSG00000031099	Smarca1	4.546E-12	-8.7770149	7.24705882	down
ENSMUSG00000031098	Syt8	5.563E-05	7.44911909	2.15555556	up
ENSMUSG00000031093	Dock11	3.013E-05	-1.099233	1.82595527	down
ENSMUSG00000031072	Oraov1	0.0002118	0.95190058	1.66345941	up
ENSMUSG00000030623	ENSMUSG00000030623	0.0003328	0.99845656	1.53274478	up
ENSMUSG00000030616	Sytl2	1.951E-09	-1.5053886	2.41869232	down
ENSMUSG00000030603	Psmc4	0.0034389	0.77290637	1.5203202	up
ENSMUSG00000030510	Cers3	0.000153	7.31244092	1.9	up
ENSMUSG00000030431	Tmem238	4.068E-08	1.4323477	2.07724645	up
ENSMUSG00000030401	Rtn2	3.697E-05	1.67529085	1.6127907	up
ENSMUSG00000030315	Vgll4	0.0028495	-0.8179154	1.54071393	down
ENSMUSG00000030257	Srgap3	4.989E-07	-1.264403	2.11495431	down
ENSMUSG00000030256	Bhlhe41	7.27E-05	-1.0795593	1.73515093	down
ENSMUSG00000030220	Arhgdib	1.34E-12	-1.7157372	2.88643039	down
ENSMUSG00000030103	Bhlhe40	0.0045158	-0.7699637	1.53238394	down
ENSMUSG00000030029	Lrig1	0.0004756	-0.8970245	1.73821741	down
ENSMUSG00000030022	Adamts9	3.77E-06	-1.3507855	1.87639238	down
ENSMUSG00000029992	Gfpt1	0.0001498	-0.9954768	1.74552549	down
ENSMUSG00000029660	Tex26	0.000643	-2.5566951	1.53781513	down
ENSMUSG00000029657	Hsph1	0.0014143	0.83509012	1.55567921	up
ENSMUSG00000029648	Flt1	0.0010573	-0.9714989	1.50308642	down
ENSMUSG00000029641	Rasl11a	5.611E-09	-1.4721341	2.34903464	down
ENSMUSG00000029640	Usp12	0.0049103	-0.7628524	1.52942779	down
ENSMUSG00000029512	Ulk1	0.000758	-0.8900515	1.64775977	down
ENSMUSG00000029470	P2rx4	0.0051256	-0.7677344	1.50947512	down

ENSMUSG00000029394	Cdk2ap1	0.0019222	0.82439401	1.5105383	up
ENSMUSG00000029385	Ccng2	1.212E-10	-1.6207561	2.57992565	down
ENSMUSG00000029380	Cxcl1	1.632E-05	-1.0815323	1.95712529	down
ENSMUSG00000029372	Ppbp	5.449E-05	-1.8938672	1.71908127	down
ENSMUSG00000029371	Cxcl5	1.713E-15	-1.8660086	3.33252856	down
ENSMUSG00000029322	Plac8	0.0004358	-1.1573034	1.51835317	down
ENSMUSG00000029309	Sparcl1	1.358E-14	-3.6943832	4.82565789	down
ENSMUSG00000029287	Tgfbr3	0.000444	-0.9271029	1.67549261	down
ENSMUSG00000029231	Pdgfra	2.582E-07	-1.2992883	2.14412395	down
ENSMUSG00000029186	Pi4k2b	9.303E-06	-1.156843	1.90623207	down
ENSMUSG00000029167	Ppargc1a	1.082E-12	-8.8548443	7.72941176	down
ENSMUSG00000029163	Emilin1	3.533E-08	-1.3437314	2.30913453	down
ENSMUSG00000029135	Fosl2	0.0079187	-0.7146543	1.5375115	down
ENSMUSG00000029126	Nsg1	0.0005667	-0.8891036	1.71962291	down
ENSMUSG00000029108	Pcdh7	3.458E-07	-1.255134	2.16777734	down
ENSMUSG00000029005	Draxin	1.346E-07	-2.6290355	2.47164948	down
ENSMUSG00000029004	Kmt2e	7.923E-05	-1.0317502	1.78115088	down
ENSMUSG00000028982	Slc25a33	0.0003244	0.9922375	1.53756789	up
ENSMUSG00000028967	Errfi1	0.0009848	-0.8744747	1.63263137	down
ENSMUSG00000028965	Tnfrsf9	2.771E-10	-1.5086727	2.60963608	down
ENSMUSG00000028926	Cdk14	0.0002125	-0.9758975	1.72034115	down
ENSMUSG00000028780	Sema3c	1.061E-16	-1.9181138	3.60513162	down
ENSMUSG00000028645	Slc2a1	1.335E-10	-1.5181568	2.6979569	down
ENSMUSG00000028636	Ppcs	4.253E-06	2.43166612	1.92802057	up
ENSMUSG00000028600	Podn	0.0001028	-1.7583114	1.64230171	down
ENSMUSG00000028527	Ak4	1.87E-06	-1.1968467	2.05415162	down
ENSMUSG00000028463	Car9	1.999E-08	-1.7363456	2.24718435	down
ENSMUSG00000028456	Unc13b	0.0001951	-3.042244	1.77604167	down
ENSMUSG00000028412	Slc44a1	0.000175	-0.9864913	1.73577782	down
ENSMUSG00000028318	Polr1e	0.0001484	1.04595687	1.58667737	up
ENSMUSG00000028211	Trp53inp1	0.0001957	-1.4939163	1.54424779	down
ENSMUSG00000028100	Nudt17	1.347E-06	1.50384815	1.81592554	up
ENSMUSG00000028069	Gpatch4	0.0006484	0.90778133	1.54797276	up
ENSMUSG00000028015	Ctso	4.767E-06	-1.7211684	1.85683297	down
ENSMUSG00000027848	Olfml3	3.543E-10	-1.6902686	2.47976879	down
ENSMUSG00000027820	Mme	7.479E-13	-2.1053878	2.970964	down
ENSMUSG00000027805	Pfn2	0.0010889	-0.9133837	1.5524895	down
ENSMUSG00000027797	Dclk1	0.0007937	-0.8719249	1.68826148	down
ENSMUSG00000027778	Ift80	0.0031575	-0.8229988	1.50863086	down
ENSMUSG00000027674	Pex5l	4.451E-13	-8.9044917	8.04705882	down
ENSMUSG00000027405	Nop56	0.0020209	0.80339584	1.58163025	up
ENSMUSG00000027368	Dusp2	9.957E-05	4.44587336	1.86885246	up
ENSMUSG00000027276	Jag1	3.481E-05	1.30801312	1.61123906	up
ENSMUSG00000027239	Mdk	5.932E-09	-1.4397729	2.38099657	down
ENSMUSG00000027207	Galk2	8.908E-05	-0.9969541	1.83394595	down
ENSMUSG00000027175	Tcp11l1	9.954E-07	-7.9316466	3.56470588	down
ENSMUSG00000027171	Prrg4	2.654E-07	-3.3832774	2.73953488	down
ENSMUSG00000026822	Lcn2	8.689E-18	-2.6910329	4.05617978	down

ENSMUSG00000026819	Slc25a25	2.01E-05	1.16668408	1.70189652	up
ENSMUSG00000026810	Dpm2	1.362E-06	1.22639939	1.92251151	up
ENSMUSG00000026785	Pkn3	0.0005803	-0.931843	1.6234338	down
ENSMUSG00000026586	Prrx1	1.615E-07	-1.265818	2.284579	down
ENSMUSG00000026471	Mr1	1.597E-06	-1.3167121	1.96309356	down
ENSMUSG00000026414	Tnnt2	2.697E-06	1.53944509	1.77965044	up
ENSMUSG00000026413	Pkp1	0.0002364	0.97930304	1.59180805	up
ENSMUSG00000026365	Cfh	0.0003003	-1.057825	1.59280959	down
ENSMUSG00000026271	Gpr35	1.07E-16	-9.3065173	11.1176471	down
ENSMUSG00000026193	Fn1	0.0057101	-0.7275111	1.65819977	down
ENSMUSG00000026069	Il1rl1	2.062E-07	-1.2774863	2.19839899	down
ENSMUSG00000026018	Ica1l	0.0001808	-1.7750408	1.59145299	down
ENSMUSG00000025986	Slc39a10	0.0008354	-0.9311575	1.57181613	down
ENSMUSG00000025969	Nrp2	0.0010771	-0.8518568	1.67187275	down
ENSMUSG00000025911	Adhfe1	7.836E-06	-2.1895261	1.95940171	down
ENSMUSG00000025888	Casp1	0.0010634	-3.4801442	1.52816901	down
ENSMUSG00000025791	Pgm2	5.555E-05	-1.008476	1.91707571	down
ENSMUSG00000025732	Fam195a	2.317E-05	1.12001027	1.7262124	up
ENSMUSG00000025532	Crcp	0.0007289	0.90890578	1.52539405	up
ENSMUSG00000025473	Adam8	3.454E-07	1.47473409	1.91454545	up
ENSMUSG00000025362	Rps26	0.0087555	0.69732084	1.50897338	up
ENSMUSG00000025347	Mettl7b	1.586E-06	-2.4752553	2.18556701	down
ENSMUSG00000025283	Sat1	9.353E-05	-0.9888267	1.84755159	down
ENSMUSG00000025227	Tmem180	1.237E-07	-2.0934942	2.21857143	down
ENSMUSG00000025161	Slc16a3	6.859E-12	-2.089564	2.83073798	down
ENSMUSG00000025025	Mxi1	0.000175	-0.9827863	1.74019414	down
ENSMUSG00000025006	Sorbs1	4.992E-10	-3.5947305	3.62184874	down
ENSMUSG00000024978	Gpam	0.003075	-0.8178913	1.52576563	down
ENSMUSG00000024924	Vldlr	8.989E-08	-1.3754508	2.18086932	down
ENSMUSG00000024913	Lrp5	1.223E-11	-1.6691469	2.74014229	down
ENSMUSG00000024810	Il33	7.756E-25	-2.9801861	5.38300936	down
ENSMUSG00000024803	Ankrd1	0.0001664	0.97349389	1.65897466	up
ENSMUSG00000024529	Lox	1.669E-18	-2.0230554	3.90732532	down
ENSMUSG00000024395	Lims2	1.347E-06	-2.3875056	2.17523364	down
ENSMUSG00000024164	C3	9.589E-26	-2.4339817	5.03301796	down
ENSMUSG00000024066	Xdh	3.732E-05	-1.0288089	1.94558802	down
ENSMUSG00000023992	Trem2	8.946E-12	-3.7995178	4.2605042	down
ENSMUSG00000023988	Bysl	7.315E-05	1.00571103	1.74038575	up
ENSMUSG00000023951	Vegfa	7.805E-06	-1.1262243	1.97666403	down
ENSMUSG00000023915	Tnfrsf21	2.038E-05	1.45703812	1.63426241	up
ENSMUSG00000023911	Flywch2	0.0009939	-4.1962251	1.57391304	down
ENSMUSG00000023905	Tnfrsf12a	2.26E-05	1.05384553	1.85713086	up
ENSMUSG00000023885	Thbs2	2.98E-11	-1.7456136	2.64539559	down
ENSMUSG00000023456	Tpi1	0.0006365	-0.866047	1.82324549	down
ENSMUSG00000023067	Cdkn1a	9.658E-16	-2.0547186	3.32572718	down
ENSMUSG00000022995	Enah	0.0056404	-0.7455974	1.54239229	down
ENSMUSG00000022912	Pros1	5.399E-28	-10.22949	22.8588235	down
ENSMUSG00000022887	Masp1	1.79E-05	-1.0830396	1.9320437	down

ENSMUSG00000022849	Hspbap1	3.138E-05	1.20700354	1.64384724	up
ENSMUSG00000022802	Lmln	0.0001545	-1.3824719	1.56895128	down
ENSMUSG00000022788	Fgd4	2.495E-06	-4.16312	2.76056338	down
ENSMUSG00000022754	Tmem45a	2.567E-10	-1.5110659	2.61544323	down
ENSMUSG00000022707	Gbe1	6.941E-11	-1.6926418	2.60212436	down
ENSMUSG00000022639	ENSMUSG00000022639	9.482E-06	-1.2449468	1.83987761	down
ENSMUSG00000022554	Fam203a	0.0006413	0.90816465	1.54462151	up
ENSMUSG00000022528	Hes1	2.316E-09	1.6985614	2.22609718	up
ENSMUSG00000022489	Pde1b	2.62E-07	-2.0705998	2.15418502	down
ENSMUSG00000022367	Has2	4.461E-10	-1.6593685	2.46787747	down
ENSMUSG00000022325	Pop1	2.097E-06	1.24981931	1.85674074	up
ENSMUSG00000022321	Cdh10	2.085E-05	7.57396181	2.41111111	up
ENSMUSG00000022280	Rnf19a	0.0014649	-0.8719751	1.56234535	down
ENSMUSG00000022150	Dab2	0.0066953	-0.737459	1.51499304	down
ENSMUSG00000022146	Osmr	0.0013436	-0.8660521	1.58662242	down
ENSMUSG00000022139	Mbnl2	0.006345	-0.7433891	1.51180812	down
ENSMUSG00000022123	Scel	0.0003053	-3.2569284	1.72619048	down
ENSMUSG00000022051	Bnip3l	8.094E-06	-1.1075456	2.02632873	down
ENSMUSG00000021994	Wnt5a	1.754E-05	-1.1548182	1.83327938	down
ENSMUSG00000021876	Rnase4	1.879E-37	-10.807543	35.4823529	down
ENSMUSG00000021831	Ero1l	4.368E-23	-2.2966059	4.57081997	down
ENSMUSG00000021798	Ldb3	6.207E-05	2.52925625	1.69	up
ENSMUSG00000021756	Il6st	0.0050606	-0.7487701	1.57018221	down
ENSMUSG00000021754	Map3k1	0.0005628	-1.0721222	1.51313814	down
ENSMUSG00000021728	Emb	0.0005316	-0.9275102	1.64553232	down
ENSMUSG00000021703	Serinc5	7.909E-06	-1.427772	1.8220293	down
ENSMUSG00000021591	Glrx	0.000175	-1.0023522	1.70961969	down
ENSMUSG00000021466	Ptch1	2.933E-06	-1.2940339	1.91604755	down
ENSMUSG00000021367	Edn1	1.203E-05	3.53384754	2.08426966	up
ENSMUSG00000021238	Aldh6a1	0.0002589	-1.1162915	1.58096591	down
ENSMUSG00000021196	Pfkp	5.001E-05	-1.0197498	1.89918898	down
ENSMUSG00000021127	Zfp36l1	0.0038875	-0.7918186	1.52473182	down
ENSMUSG00000021109	Hif1a	1.581E-05	-1.0802996	1.97054978	down
ENSMUSG00000021108	Prkch	7.836E-06	-7.7261045	2.97647059	down
ENSMUSG00000021091	Serpina3n	3.751E-16	-9.250025	10.6235294	down
ENSMUSG00000021070	Bdkrb2	3.188E-05	-1.2545634	1.72675086	down
ENSMUSG00000020902	Ntn1	1.071E-06	-1.208232	2.10575966	down
ENSMUSG00000020901	Pik3r5	2.627E-07	-1.4803627	2.05374351	down
ENSMUSG00000020876	Snx11	0.0004358	0.97223438	1.51764706	up
ENSMUSG00000020828	Pld2	7.756E-25	-3.2186615	5.78085106	down
ENSMUSG00000020826	Nos2	5.23E-49	-3.8270296	11.1802526	down
ENSMUSG00000020695	Mrc2	0.0031989	-0.7736251	1.65044888	down
ENSMUSG00000020684	Rasl10b	0.0006244	-4.2661931	1.67826087	down
ENSMUSG00000020601	Trib2	3.543E-10	-1.6721269	2.48266297	down
ENSMUSG00000020572	Nampt	0.0021295	-0.8210653	1.59139484	down
ENSMUSG00000020514	Mrpl22	6.413E-05	1.02532679	1.71555664	up
ENSMUSG00000020375	Rufy1	6.075E-05	1.06353212	1.66759227	up
ENSMUSG00000020363	Gfpt2	0.0004413	-2.3766583	1.57446809	down

ENSMUSG00000020312	Shc2	0.0008819	-0.9737561	1.52402474	down
ENSMUSG00000020277	Pfkl	5.177E-05	-1.0067056	1.95876795	down
ENSMUSG00000020123	Avpr1a	4.727E-08	-3.3321896	2.92857143	down
ENSMUSG00000020108	Ddit4	0.0003394	-0.9338572	1.71177056	down
ENSMUSG00000019997	Ctgf	7.591E-13	1.79683152	2.71053529	up
ENSMUSG00000019947	Arid5b	0.0003394	-0.9504461	1.68143024	down
ENSMUSG00000019944	Rhobtb1	0.0002741	-1.1384139	1.5668147	down
ENSMUSG00000019916	P4ha1	0.0001545	-0.9549904	1.85057876	down
ENSMUSG00000019880	Rspo3	3.996E-05	-1.1655004	1.74047141	down
ENSMUSG00000019359	Gdpd2	8.776E-13	-3.1492781	3.84313725	down
ENSMUSG00000019189	Rnf145	0.0076018	-0.7286214	1.50115265	down
ENSMUSG00000019102	Aldh3a1	4.444E-07	-1.459294	2.01726727	down
ENSMUSG00000018906	P4ha2	0.0078965	-0.7121471	1.55280714	down
ENSMUSG00000018740	Slc25a35	5.759E-05	1.0617446	1.67947217	up
ENSMUSG00000018585	Atox1	0.0045158	0.75217212	1.50748455	up
ENSMUSG00000018509	Cenpv	4.591E-09	1.48913094	2.21600799	up
ENSMUSG00000018427	Ypel2	0.0002146	-1.1751881	1.5707804	down
ENSMUSG00000018411	Mapt	8.022E-05	1.75665602	1.53101362	up
ENSMUSG00000017737	Mmp9	1.697E-07	-4.3792622	3.30985915	down
ENSMUSG00000017607	Tns4	0.0009037	-2.8565719	1.51041667	down
ENSMUSG00000017446	C1qtnf1	3.378E-06	-1.3048056	1.8957927	down
ENSMUSG00000017400	Stac2	4.79E-64	-9.0281264	72.6173913	down
ENSMUSG00000017314	Mpp2	1.621E-08	-1.4864435	2.25355099	down
ENSMUSG00000017167	Cntnap1	1.024E-07	-1.4493348	2.12872841	down
ENSMUSG00000017057	Il13ra1	9.175E-05	-1.0132151	1.79391964	down
ENSMUSG00000017002	Slpi	7.922E-15	-1.8747401	3.2081055	down
ENSMUSG00000016552	Foxred2	1.645E-08	-1.479225	2.25391658	down
ENSMUSG00000016458	Wt1	8.061E-08	-1.4167083	2.16128393	down
ENSMUSG00000016024	Lbp	8.6E-05	-1.1663462	1.66699251	down
ENSMUSG00000015766	Eps8	0.0006413	-0.8858099	1.69696585	down
ENSMUSG00000015653	Steap2	0.0069827	-0.7239961	1.5541429	down
ENSMUSG00000015605	Srf	0.0003797	0.91893252	1.62467227	up
ENSMUSG00000015568	Lpl	4.199E-05	-1.0757965	1.81228814	down
ENSMUSG00000015501	Hivep2	2.837E-05	-1.180178	1.77034184	down
ENSMUSG00000015354	Pcolce2	0.0010806	0.87788465	1.50793261	up
ENSMUSG00000015243	Abca1	4.272E-18	-2.3630854	3.78577858	down
ENSMUSG00000015176	Nolc1	0.00431	0.75576344	1.50908661	up
ENSMUSG00000014905	Dnajb9	0.0004264	-1.1164804	1.52544529	down
ENSMUSG00000013846	St3gal1	1.581E-05	-1.1311337	1.87252221	down
ENSMUSG00000013663	Pten	6.055E-05	-1.0192646	1.85282268	down
ENSMUSG00000012848	Rps5	0.0056118	0.72983985	1.54417607	up
ENSMUSG00000012777	Acpt	5.105E-05	1.3294784	1.57072368	up
ENSMUSG00000012428	Steap4	2.333E-33	-7.5173614	23.6521739	down
ENSMUSG00000011752	Pgam1	0.0019372	-0.8019793	1.71759607	down
ENSMUSG00000009563	Tor2a	3.837E-05	1.06366548	1.73402967	up
ENSMUSG00000009185	Ccl8	1.283E-07	-2.0012215	2.17654477	down
ENSMUSG00000008540	Mgst1	1.712E-07	-1.3246347	2.15965279	down
ENSMUSG00000007888	Crlf1	3.699E-06	1.17505031	1.87721298	up

ENSMUSG00000007817	Zmiz1	0.0018188	-0.822718	1.62731192	down
ENSMUSG00000007033	Hspa1l	3.945E-06	1.77197872	1.79744136	up
ENSMUSG00000006494	Pdk1	6.576E-18	-2.0432096	3.69386412	down
ENSMUSG00000006403	Adamts4	1.547E-09	-1.9258793	2.44863267	down
ENSMUSG00000006335	Tfpt	1.365E-05	1.16044763	1.74672991	up
ENSMUSG00000005800	Mmp8	9.954E-07	-2.7001246	2.31384615	down
ENSMUSG00000005686	Ampd3	3.265E-10	-1.5657523	2.53377016	down
ENSMUSG00000005534	Insr	0.0012664	-0.9090988	1.53648821	down
ENSMUSG00000005374	Tbl2	0.0001573	-0.9765045	1.76784847	down
ENSMUSG00000005125	Ndrg1	1.545E-20	-2.1400389	4.20896621	down
ENSMUSG00000004933	Matk	0.000112	1.9146832	1.52611219	up
ENSMUSG00000004655	Aqp1	2.806E-22	-2.7283009	4.69802555	down
ENSMUSG00000004558	Ndrg2	3.206E-09	-2.019456	2.43619048	down
ENSMUSG00000004319	Clcn3	5.35E-05	-1.0567025	1.80717784	down
ENSMUSG00000004267	Eno2	6.248E-09	-1.4270172	2.38880484	down
ENSMUSG00000003955	Fam162a	1.193E-05	-1.084674	2.02850949	down
ENSMUSG00000003873	Bax	1.146E-05	1.10310826	1.84929142	up
ENSMUSG00000003865	Gys1	0.0027035	-0.7908152	1.63087837	down
ENSMUSG00000003721	Insig2	0.0004573	-0.9147269	1.69090465	down
ENSMUSG00000003617	Cp	1.706E-15	-1.8422317	3.42543998	down
ENSMUSG00000003541	Ier3	1.567E-05	-1.0793897	1.97676425	down
ENSMUSG00000003355	Fkbp11	2.219E-15	-3.3680826	4.54901961	down
ENSMUSG00000003031	Cdkn1b	0.0033233	-0.8025456	1.53610945	down
ENSMUSG00000002984	Tomm40	0.0020828	0.80669857	1.55160938	up
ENSMUSG00000002409	Dyrk1b	3.872E-05	-1.2299747	1.72017045	down
ENSMUSG00000002107	Celf2	0.0003949	-0.9602394	1.64515738	down
ENSMUSG00000001768	Rin2	3.123E-06	-1.2317506	1.94662088	down
ENSMUSG00000001751	Naglu	0.0007375	-0.9017382	1.63341165	down
ENSMUSG00000001542	Ell2	0.0003121	-1.086334	1.58173831	down
ENSMUSG00000001156	Mxd1	0.0003355	-1.1330238	1.54452926	down
ENSMUSG00000001056	Nhp2	0.0016832	0.81899811	1.56645739	up
ENSMUSG00000000690	Hoxb6	2.181E-07	1.64140528	1.94391408	up
ENSMUSG00000000628	Hk2	0.0001004	-1.0161046	1.77555739	down
ENSMUSG00000000489	Pdgfb	1.294E-05	1.2077314	1.72708548	up

S2_Compared with mouse brain proteome at 9 days after birth				
#ID	preferredName	FDR	log2FC	regulated
ENSMUSG00000078812	Eif5a	0.002465404	0.785727733	up
ENSMUSG00000071658	Gng3	8.97E-05	3.053238863	up
ENSMUSG00000067629	Syngap1	3.07E-05	-1.247757824	down
ENSMUSG00000063229	Ldha	0.001338216	-0.821725056	down
ENSMUSG00000062380	Tubb3	4.00E-05	1.089360032	up
ENSMUSG00000062070	Pgk1	7.27E-05	-0.984571101	down
ENSMUSG00000060961	Slc4a4	1.25E-07	-1.631462097	down
ENSMUSG00000059456	Ptk2b	2.74E-06	-7.83253293	down
ENSMUSG00000058420	Syt17	1.74E-06	-1.380660381	down
ENSMUSG00000057666	Gapdh	0.002800884	-0.784715839	down
ENSMUSG00000057193	Slc44a2	0.007781326	-0.71895963	down
ENSMUSG00000055782	Abcd2	1.55E-09	-2.473152044	down
ENSMUSG00000054793	Cadm4	6.73E-05	1.342584155	up
ENSMUSG00000053702	Nebl	7.43E-06	1.858746262	up
ENSMUSG00000050965	Prkca	0.000183553	-0.986622721	down
ENSMUSG00000050953	Gja1	7.14E-05	-1.008720065	down
ENSMUSG00000045092	S1pr1	1.06E-05	-1.388749723	down
ENSMUSG00000043991	Pura	2.81E-22	-2.836144751	down
ENSMUSG00000041757	Plekha6	0.000129248	-1.435663953	down
ENSMUSG00000041642	Kif21b	0.006388234	-0.736533789	down
ENSMUSG00000040675	Mthfd1l	0.006258581	-0.733361159	down
ENSMUSG00000040249	Lrp1	0.006012088	-0.728224954	down
ENSMUSG00000039704	Lmbrd2	0.000129248	-1.047285741	down
ENSMUSG00000039474	Wfs1	8.62E-05	-1.017260216	down
ENSMUSG00000038587	Akap12	1.18E-06	1.311067129	up
ENSMUSG00000037434	Slc30a1	7.27E-05	1.266044465	up
ENSMUSG00000037012	Hk1	0.000668538	-0.873565288	down
ENSMUSG00000035674	Ndufa3	0.00102346	0.886115743	up
ENSMUSG00000033066	Gas7	2.61E-13	-2.192368736	down
ENSMUSG00000032625	Thsd7a	0.000387198	-1.030493802	down
ENSMUSG00000030603	Psmc4	0.003438938	0.772906375	up
ENSMUSG00000030257	Srgap3	4.99E-07	-1.264403032	down
ENSMUSG00000029657	Hsph1	0.0014143	0.835090121	up
ENSMUSG00000029309	Sparcl1	1.36E-14	-3.694383242	down
ENSMUSG00000029126	Nsg1	0.000566745	-0.889103578	down
ENSMUSG00000028645	Slc2a1	1.33E-10	-1.518156759	down
ENSMUSG00000028527	Ak4	1.87E-06	-1.19684666	down
ENSMUSG00000027805	Pfn2	0.001088938	-0.913383676	down
ENSMUSG00000027797	Dclk1	0.000793653	-0.871924886	down
ENSMUSG00000027674	Pex5l	4.45E-13	-8.90449172	down
ENSMUSG00000026819	Slc25a25	2.01E-05	1.166684084	up
ENSMUSG00000025986	Slc39a10	0.000835358	-0.93115745	down
ENSMUSG00000025006	Sorbs1	4.99E-10	-3.594730482	down
ENSMUSG00000023456	Tpi1	0.000636521	-0.866046976	down
ENSMUSG00000022995	Enah	0.005640374	-0.745597391	down
ENSMUSG00000022489	Pde1b	2.62E-07	-2.070599758	down

ENSMUSG00000022321	Cdh10	2.08E-05	7.573961813	up
ENSMUSG00000021238	Aldh6a1	0.000258937	-1.116291497	down
ENSMUSG00000021196	Pfkp	5.00E-05	-1.019749826	down
ENSMUSG00000020277	Pfkl	5.18E-05	-1.006705583	down
ENSMUSG00000018411	Mapt	8.02E-05	1.756656016	up
ENSMUSG00000017314	Mpp2	1.62E-08	-1.486443482	down
ENSMUSG00000017167	Cntnap1	1.02E-07	-1.449334804	down
ENSMUSG00000011752	Pgam1	0.001937196	-0.801979285	down
ENSMUSG00000006494	Pdk1	6.58E-18	-2.043209637	down
ENSMUSG00000005534	Insr	0.001266429	-0.909098754	down
ENSMUSG00000004933	Matk	0.000111965	1.914683198	up
ENSMUSG00000004558	Ndrg2	3.21E-09	-2.019455981	down
ENSMUSG00000004319	Clcn3	5.35E-05	-1.056702486	down
ENSMUSG00000004267	Eno2	6.25E-09	-1.427017178	down
ENSMUSG00000002984	Tomm40	0.002082753	0.806698573	up
ENSMUSG00000002107	Celf2	0.000394922	-0.960239442	down

S3_peaks						
chr	start	end	length	abs_summit	pileup	-LOG10(pvalue)
1	5562098	5562403	306	5562151	7	4.65491
1	6789646	6789977	332	6789787	7	5.30623
1	7278301	7278639	339	7278431	8	5.56774
1	7623340	7623677	338	7623450	8	6.50668
1	8806804	8807137	334	8806919	5	3.5422
1	9220143	9220558	416	9220267	7	5.47978
1	9286977	9287302	326	9287179	8	5.39858
1	11354061	11354378	318	11354237	5	3.4561
1	14811116	14811560	445	14811377	7	5.13754
1	15331177	15331491	315	15331341	7	5.47978
1	15549407	15549764	358	15549750	6	4.50291
1	15574513	15574826	314	15574763	5	3.58241
1	17250808	17251153	346	17251116	5	3.58241
1	19846210	19846573	364	19846386	6	3.8181
1	23809992	23810331	340	23810136	7	5.47978
1	28601824	28602281	458	28601929	6	3.66421
1	28641549	28642143	595	28641832	7	3.45771
1	28940196	28940496	301	28940365	6	4.40359
1	30363117	30363496	380	30363331	6	4.50291
1	30489967	30490459	493	30490197	5	3.58241
1	32682379	32682727	349	32682481	8	6.50668
1	40676211	40676547	337	40676215	5	3.58241
1	46953494	46953898	405	46953499	6	3.79171
1	47110085	47110436	352	47110411	6	3.79171
1	47647089	47647429	341	47647316	6	4.50291
1	47931683	47932260	578	47931916	7	5.47978
1	49023762	49024088	327	49023901	6	4.50291
1	49312374	49312727	354	49312529	7	5.47978
1	50807658	50808139	482	50808048	6	3.66421
1	51996794	51997112	319	51996900	7	4.87731
1	54301815	54302123	309	54301999	6	3.98315
1	54729097	54729429	333	54729427	8	3.3645
1	55238636	55238940	305	55238839	8	4.94122
1	56060737	56061055	319	56060906	6	4.50291
1	57864455	57864788	334	57864567	7	5.47978
1	68310542	68310929	388	68310546	6	4.50291
1	68649985	68650325	341	68650162	5	3.07446
1	70236462	70236796	335	70236640	8	6.50668
1	71100622	71100925	304	71100705	10	5.80609
1	71252000	71252344	345	71252320	9	6.52524
1	73553394	73553738	345	73553638	7	3.45771
1	73760124	73760470	347	73760285	7	5.47978
1	77641738	77642082	345	77641751	6	4.50291
1	77911065	77911523	459	77911134	7	3.45771
1	78987666	78988007	342	78987837	8	6.50668
1	79041098	79041521	424	79041241	7	5.47978

1	81168862	81169234	373	81169067	6	4.50291
1	83284943	83285282	340	83285121	8	5.56774
1	83820711	83821339	629	83821205	7	4.97847
1	85095391	85095768	378	85095583	8	5.02727
1	85317363	85318242	880	85317871	11	3.12903
1	88213568	88213916	349	88213587	6	3.79171
1	92098519	92098818	300	92098712	7	4.65491
1	93278757	93279079	323	93278940	6	4.50291
1	93885875	93886175	301	93885885	7	3.45771
1	94303728	94304097	370	94303884	8	4.19779
1	94569676	94569982	307	94569719	7	5.47978
1	94946891	94947217	327	94947073	8	5.56774
1	95830730	95831045	316	95830901	9	7.57852
1	95887271	95887615	345	95887494	5	3.58241
1	96842092	96842463	372	96842198	8	5.56774
1	99187245	99187547	303	99187500	8	5.3496
1	99211338	99211670	333	99211540	6	4.50291
1	100461890	100462380	491	100462098	7	4.87731
1	100598187	100598560	374	100598299	7	4.65491
1	103297781	103298134	354	103298089	6	4.50291
1	103819828	103820154	327	103819998	6	4.50291
1	105642636	105642940	305	105642837	8	5.93643
1	106259850	106260152	303	106260125	6	4.40359
1	107108328	107108668	341	107108342	7	4.50662
1	108790491	108790861	371	108790711	8	5.56774
1	109453606	109453918	313	109453701	7	3.76048
1	109491023	109491400	378	109491082	8	4.19779
1	109638114	109638431	318	109638339	6	4.50291
1	109944462	109944931	470	109944616	8	4.19779
1	110674239	110674589	351	110674501	7	5.47978
1	110765877	110766180	304	110766084	7	4.50662
1	111493476	111493848	373	111493588	6	4.40359
1	112213063	112213392	330	112213073	5	3.58241
1	112988328	112988646	319	112988399	6	4.50291
1	114696527	114696861	335	114696636	5	3.58241
1	114863947	114864263	317	114863965	6	4.50291
1	115290203	115290547	345	115290434	8	6.50668
1	115452208	115452577	370	115452370	9	7.50822
1	116115537	116115990	454	116115583	6	4.25501
1	118123578	118123942	365	118123795	6	4.50291
1	118848983	118849282	300	118849262	6	4.50291
1	121967447	121967771	325	121967519	7	4.50662
1	123261120	123261543	424	123261455	6	4.50291
1	123668020	123668424	405	123668067	10	7.52331
1	123773366	123773667	302	123773644	9	3.94891
1	123793158	123793807	650	123793767	6	3.79171
1	124006716	124007037	322	124006762	5	3.58241
1	125844566	125844875	310	125844766	5	3.58241

1	127763759	127764074	316	127763905	6	4.07032
1	128067835	128068135	301	128067862	5	3.58241
1	129947200	129947529	330	129947468	8	5.49896
1	130793866	130794235	370	130794072	8	4.19779
1	133530353	133530657	305	133530512	6	4.50291
1	134690149	134690465	317	134690297	5	3.58241
1	138903598	138903913	316	138903688	6	3.85827
1	140752664	140752969	306	140752886	6	3.05245
1	140916064	140916363	300	140916134	5	3.25592
1	141103562	141103989	428	141103613	6	3.79171
1	141469744	141470098	355	141469939	7	3.1897
1	141847979	141848511	533	141848110	7	4.97847
1	141983115	141983421	307	141983338	6	4.45522
1	142961845	142962239	395	142962158	6	4.50291
1	143075535	143075954	420	143075855	7	3.45771
1	144908522	144908929	408	144908741	7	4.65491
1	145081646	145081996	351	145081673	6	4.50291
1	145480658	145480993	336	145480737	7	3.45771
1	145905687	145906020	334	145905868	6	3.3206
1	146183194	146183521	328	146183242	6	4.50291
1	146676748	146677112	365	146677063	7	5.47978
1	147095239	147095553	315	147095255	6	4.50291
1	147129567	147129929	363	147129629	6	4.02633
1	147869595	147869942	348	147869854	7	5.42458
1	147884873	147885324	452	147884898	7	5.42458
1	148028675	148029016	342	148028871	8	6.50668
1	148108015	148108382	368	148108244	7	4.65491
1	148250211	148250576	366	148250529	6	4.50291
1	148571015	148571380	366	148571210	6	3.79171
1	148576544	148576901	358	148576568	5	3.41441
1	148849450	148849788	339	148849658	6	3.08059
1	149229546	149229968	423	149229711	8	5.93643
1	149609262	149609617	356	149609465	5	3.25592
1	152297973	152298316	344	152297984	7	3.45771
1	153552171	153552549	379	153552499	7	3.45771
1	154592318	154592638	321	154592519	7	5.47978
1	154617193	154617516	324	154617382	6	4.50291
1	154743945	154744261	317	154744101	5	3.58241
1	157614069	157614531	463	157614480	6	4.40359
1	157734798	157735097	300	157735048	6	4.50291
1	157870925	157871269	345	157871100	5	3.58241
1	158439527	158439838	312	158439552	7	3.1897
1	158482583	158482883	301	158482804	6	4.07032
1	159040055	159040519	465	159040240	8	6.30939
1	161482140	161482480	341	161482415	8	4.69722
1	164740907	164741297	391	164741140	8	6.50668
1	164981705	164982030	326	164981777	6	3.62733
1	165253738	165254089	352	165253872	8	5.56774

1	166542288	166542651	364	166542549	8	6.50668
1	166676785	166677127	343	166676954	7	4.97847
1	166915571	166915925	355	166915799	9	6.33515
1	168530718	168531065	348	168530825	7	3.45771
1	169015485	169015815	331	169015507	5	3.5422
1	169321746	169322064	319	169321808	7	5.47978
1	170237058	170237411	354	170237160	6	4.35303
1	171581243	171581626	384	171581320	7	4.06945
1	172421260	172421654	395	172421383	7	4.87731
1	173205654	173205959	306	173205903	6	3.66421
1	173444958	173445260	303	173444982	5	3.58241
1	174812243	174812551	309	174812480	8	4.94122
1	175081468	175081814	347	175081806	6	3.79171
1	176568033	176568509	477	176568155	6	4.50291
1	177912487	177912933	447	177912845	8	4.10153
1	178009317	178009704	388	178009529	5	3.49869
1	178815468	178815816	349	178815655	8	6.30939
1	180558803	180559193	391	180559121	9	5.37431
1	181266095	181266480	386	181266266	10	7.52331
1	182215595	182215952	358	182215877	9	6.52524
1	184371892	184372249	358	184372059	9	4.63301
1	186025751	186026066	316	186025886	8	6.37597
1	187113183	187113533	351	187113263	5	3.37357
1	193060771	193061074	304	193060895	7	5.47978
1	194122289	194122598	310	194122359	5	3.21823
1	194615060	194615379	320	194615230	7	4.10626
1	195059547	195059879	333	195059697	6	4.50291
10	3116337	3116704	368	3116422	14	3.16431
10	5435426	5435756	331	5435567	6	4.30352
10	5841167	5841585	419	5841485	9	4.98189
10	5846963	5847487	525	5847386	5	3.00646
10	10611702	10612023	322	10611737	5	3.5422
10	11943651	11943970	320	11943809	7	4.65491
10	13896820	13897177	358	13897028	8	6.50668
10	15074504	15075036	533	15074795	7	3.45771
10	15104496	15104828	333	15104534	6	4.20747
10	15269811	15270133	323	15269999	6	4.50291
10	15858982	15859354	373	15859052	6	3.79171
10	16299522	16299895	374	16299547	6	4.50291
10	23040204	23040528	325	23040334	5	3.33356
10	24232990	24233293	304	24233268	6	4.50291
10	24630473	24630814	342	24630627	6	4.50291
10	24717440	24717777	338	24717642	5	3.58241
10	27262605	27262904	300	27262807	6	4.50291
10	27553354	27553709	356	27553665	6	3.98315
10	27700885	27701229	345	27701215	6	4.40359
10	28158353	28158723	371	28158457	5	3.58241
10	30819664	30820078	415	30819805	9	7.57852

10	30903659	30904005	347	30903684	8	3.28682
10	31986906	31987279	374	31987136	8	5.65592
10	33707687	33708004	318	33707845	5	3.58241
10	35758757	35759078	322	35758959	7	5.47978
10	36061454	36061767	314	36061490	7	4.87731
10	37288747	37289080	334	37288997	5	3.04015
10	40120872	40121180	309	40121104	8	4.19779
10	40942705	40943028	324	40942840	5	3.58241
10	44469908	44470257	350	44470102	7	5.47978
10	45503840	45504340	501	45504115	7	5.47978
10	45529869	45530211	343	45530051	7	4.65491
10	45779665	45780077	413	45779789	7	5.42458
10	46329824	46330148	325	46330124	5	3.37357
10	46493157	46493484	328	46493338	6	4.07032
10	47176440	47176740	301	47176498	7	4.50662
10	48030149	48030534	386	48030376	7	5.47978
10	48234867	48235181	315	48234974	7	4.65491
10	49991804	49992103	300	49991813	6	3.66421
10	50558707	50559028	322	50558872	7	5.47978
10	51652881	51653233	353	51653114	6	4.50291
10	52055081	52055427	347	52055317	5	3.58241
10	52082014	52082344	331	52082219	6	4.45522
10	54144290	54144641	352	54144368	9	4.98189
10	54662480	54662880	401	54662736	5	3.58241
10	55266243	55266553	311	55266438	7	4.65491
10	55690427	55690774	348	55690435	5	3.58241
10	55764179	55764516	338	55764366	6	4.50291
10	56146183	56146519	337	56146227	5	3.58241
10	63507745	63508061	317	63507904	7	4.65491
10	64490049	64490410	362	64490174	8	6.11749
10	64783711	64784055	345	64784015	5	3.58241
10	65688742	65689108	367	65688812	6	4.02633
10	68225148	68225517	370	68225441	6	4.07032
10	71587363	71587671	309	71587511	5	3.58241
10	71631960	71632311	352	71632138	8	5.39858
10	72416549	72416851	303	72416674	7	5.47978
10	72771915	72772263	349	72771959	5	3.41441
10	74114710	74115061	352	74114977	8	6.50668
10	74283400	74283705	306	74283591	6	4.07032
10	76922462	76922789	328	76922724	5	3.58241
10	83789370	83789756	387	83789656	9	4.98189
10	89159249	89159632	384	89159334	7	4.65491
10	89281686	89281987	302	89281924	7	5.47978
10	90994526	90995252	727	90994849	5	3.58241
10	91945476	91945810	335	91945512	7	4.65491
10	92388095	92388415	321	92388187	10	7.52331
10	98001196	98001567	372	98001215	6	4.50291
10	102431936	102432293	358	102431952	5	3.58241

10	104381761	104382107	347	104381983	6	4.50291
10	104766223	104766811	589	104766699	6	4.50291
10	105207303	105207682	380	105207437	7	5.47978
10	106236206	106236537	332	106236322	8	6.50668
10	106520665	106521002	338	106520815	6	4.11516
10	109957881	109958190	310	109957901	5	3.33356
10	112003066	112003397	332	112003268	6	3.85827
10	112055410	112055840	431	112055567	7	4.65491
10	112142895	112143254	360	112143125	7	4.65491
10	112589196	112589626	431	112589449	8	6.50668
10	113714504	113714829	326	113714716	8	4.19779
10	113725281	113725616	336	113725490	6	4.07032
10	114168442	114168749	308	114168705	8	5.56774
10	114193549	114193961	413	114193893	6	4.50291
10	120349421	120349753	333	120349582	7	4.65491
10	121498505	121498827	323	121498572	6	4.50291
10	122206105	122206429	325	122206215	9	6.52524
10	123479562	123479870	309	123479616	8	6.44394
10	123489704	123490041	338	123489921	6	4.50291
10	123701000	123701300	301	123701117	8	5.93643
10	124022655	124023021	367	124022971	6	4.50291
10	124753586	124753886	301	124753753	5	3.58241
10	124953242	124953609	368	124953505	7	5.47978
10	125065551	125065903	353	125065710	7	3.45771
10	129175647	129176166	520	129175776	8	5.87829
10	129405104	129405683	580	129405420	9	4.98189
10	129466614	129466969	356	129466670	6	4.16086
10	129928744	129929079	336	129928770	7	4.65491
10	130123681	130124026	346	130123740	9	7.57852
11	3439472	3439771	300	3439502	6	4.50291
11	5382921	5383277	357	5383075	7	4.65491
11	5639992	5640348	357	5640224	6	4.50291
11	9591430	9591770	341	9591604	6	4.50291
11	9606110	9606429	320	9606159	6	3.79171
11	10413750	10414191	442	10414021	8	6.50668
11	10817134	10817439	306	10817344	5	3.58241
11	12757281	12757650	370	12757417	7	5.47978
11	13069750	13070051	302	13069851	7	5.42458
11	13159759	13160062	304	13159959	5	3.4561
11	14485377	14485712	336	14485506	5	3.58241
11	15710680	15710998	319	15710888	6	4.20747
11	16333269	16333633	365	16333556	5	3.33356
11	18265631	18265941	311	18265786	7	5.47978
11	18731573	18732073	501	18731790	7	5.47978
11	19085267	19085604	338	19085389	7	5.24887
11	21285319	21285656	338	21285618	5	3.21823
11	23922683	23922994	312	23922696	6	4.50291
11	26227333	26227674	342	26227609	6	4.50291

11	27869171	27869558	388	27869415	9	7.35743
11	27907819	27908194	376	27907960	7	4.65491
11	27996411	27996742	332	27996633	6	4.50291
11	28776843	28777160	318	28776890	9	5.82058
11	28820371	28820700	330	28820482	5	3.14499
11	31326890	31327218	329	31326970	7	5.47978
11	33932847	33933186	340	33932878	5	3.58241
11	36907255	36907623	369	36907352	7	4.65491
11	38071910	38072223	314	38072049	7	5.47978
11	40241820	40242208	389	40241998	7	4.65491
11	42543635	42543940	306	42543759	6	4.50291
11	43321697	43322321	625	43322203	9	7.57852
11	45489341	45489642	302	45489416	5	3.58241
11	46248002	46248355	354	46248180	7	5.47978
11	47634799	47635139	341	47635136	6	3.02469
11	47798219	47798608	390	47798439	6	4.50291
11	50443408	50443752	345	50443472	8	5.56774
11	51154963	51155302	340	51154985	6	4.50291
11	56637488	56637840	353	56637535	7	5.47978
11	58409891	58410221	331	58410063	7	4.97847
11	58471350	58471702	353	58471542	9	7.57852
11	65579268	65579606	339	65579341	7	4.65491
11	65719975	65720290	316	65720129	8	6.44394
11	66842973	66843303	331	66843297	6	4.50291
11	72023238	72023576	339	72023520	6	3.79171
11	73646315	73646692	378	73646318	6	3.79171
11	73690287	73690675	389	73690419	9	5.96698
11	73862535	73862891	357	73862855	6	4.50291
11	78124872	78125363	492	78125093	5	3.58241
11	81753831	81754292	462	81753870	7	4.65491
11	82701387	82701708	322	82701570	5	3.29435
11	83210102	83210402	301	83210341	6	4.02633
11	84617085	84617401	317	84617298	5	3.58241
11	87365548	87365918	371	87365833	6	4.50291
11	87422678	87423156	479	87423083	10	5.13367
11	87462299	87462904	606	87462388	17	11.18385
11	89854409	89854731	323	89854509	6	3.79171
11	92189008	92189472	465	92189089	7	4.10626
11	93281166	93281479	314	93281219	5	3.58241
11	93395580	93395943	364	93395625	6	3.79171
11	99551159	99551460	302	99551302	7	5.30623
11	100921012	100921400	389	100921338	5	3.41441
11	101658330	101658911	582	101658753	10	5.41652
11	102102047	102102462	416	102102142	6	4.50291
11	103444986	103445466	481	103445048	6	4.50291
11	104962833	104963308	476	104963117	7	4.65491
11	109011704	109012031	328	109011869	53	18.64715
11	111225428	111225795	368	111225481	7	4.73225

11	112152908	112153232	325	112153177	6	4.50291
11	114022953	114023294	342	114023160	6	3.79171
11	121258736	121259062	327	121258785	8	6.24414
11	121372282	121372657	376	121372417	5	3.4561
11	121397487	121397793	307	121397719	5	3.29435
12	6805902	6806254	353	6805929	6	3.79171
12	7034427	7034869	443	7034525	5	3.58241
12	7717324	7717626	303	7717371	5	3.58241
12	13762114	13762414	301	13762118	6	4.50291
12	14690623	14690984	362	14690793	6	4.50291
12	22147715	22148021	307	22147878	5	3.58241
12	30821784	30822137	354	30822040	12	5.12228
12	31360833	31361154	322	31361105	8	5.56774
12	32048397	32048751	355	32048644	6	4.11516
12	39069939	39070275	337	39070169	5	3.58241
12	40375029	40375420	392	40375278	7	4.65491
12	44260317	44260649	333	44260328	5	3.41441
12	49759261	49759613	353	49759413	7	5.47978
12	51386795	51387104	310	51386960	5	3.58241
12	68689170	68689521	352	68689331	7	5.47978
12	69608357	69608771	415	69608643	24	27.25352
12	77551750	77552292	543	77551863	9	7.57852
12	83678077	83678399	323	83678220	5	3.4561
12	84519119	84519482	364	84519213	6	4.50291
12	91167441	91167849	409	91167494	7	5.47978
12	94469450	94469782	333	94469524	6	4.50291
12	94622046	94622366	321	94622240	7	4.65491
12	96742311	96742618	308	96742576	7	5.24887
12	97675029	97675474	446	97675102	6	4.50291
12	99823703	99824050	348	99823802	7	5.47978
12	101355514	101355848	335	101355687	5	3.58241
12	107812103	107812447	345	107812118	6	4.50291
12	117539818	117540164	347	117540007	5	3.58241
12	117626061	117626390	330	117626241	6	3.79171
12	120028670	120029002	333	120028844	30	21.28436
13	3112205	3112572	368	3112480	7	5.47978
13	6880197	6880551	355	6880368	5	3.58241
13	8764064	8764386	323	8764159	6	3.45162
13	13860611	13861002	392	13860864	32	39.551
13	16669448	16669757	310	16669560	6	4.50291
13	21601266	21601587	322	21601446	7	4.65491
13	21630285	21630973	689	21630778	9	6.52524
13	51561516	51562039	524	51561543	7	4.50662
13	52307318	52307627	310	52307457	8	6.50668
13	55041202	55041533	332	55041366	5	3.58241
13	65444347	65444741	395	65444611	8	5.56774
13	65904378	65904809	432	65904543	9	7.57852
13	66002811	66003211	401	66002949	7	5.47978

13	66459708	66460036	329	66459747	5	3.58241
13	66633651	66633950	300	66633651	7	3.45771
13	66644174	66644544	371	66644206	7	5.47978
13	66755293	66755761	469	66755656	6	4.50291
13	68080034	68080400	367	68080064	6	4.50291
13	77682010	77682391	382	77682250	7	5.47978
13	80025687	80026059	373	80025844	7	4.65491
13	81506956	81507302	347	81506993	6	4.50291
13	86599593	86599918	326	86599656	5	3.58241
13	88661531	88661927	397	88661662	6	3.79171
13	90444717	90445022	306	90444856	5	3.58241
13	94739169	94739573	405	94739417	6	4.50291
13	95506779	95507164	386	95506969	8	6.50668
13	96848232	96848749	518	96848599	6	3.79171
13	98495629	98496012	384	98495768	25	28.73729
13	106375096	106375451	356	106375267	8	6.50668
13	108598087	108598443	357	108598165	6	4.45522
13	118178967	118179539	573	118179112	7	5.19265
13	118258055	118258366	312	118258257	6	4.50291
13	119491694	119492077	384	119491740	16	4.50822
14	3217078	3217545	468	3217155	6	4.35303
14	3361275	3361706	432	3361600	8	4.19779
14	3420477	3420800	324	3420666	8	6.50668
14	3981105	3981412	308	3981126	8	5.11589
14	4194468	4194800	333	4194757	6	3.66421
14	4270950	4271314	365	4271313	8	3.28682
14	4383893	4384278	386	4384120	9	6.52524
14	4687882	4688233	352	4688035	7	5.36479
14	4811588	4811906	319	4811780	9	4.6997
14	4816555	4816918	364	4816912	8	4.00857
14	4853212	4853565	354	4853490	8	6.50668
14	5078128	5078574	447	5078268	6	3.8181
14	5308250	5308755	506	5308569	8	4.19779
14	5973019	5973524	506	5973502	6	3.79171
14	6139592	6139931	340	6139621	6	3.3206
14	6205823	6206169	347	6206075	12	4.31384
14	6213759	6214073	315	6213988	9	4.98189
14	6260387	6260971	585	6260702	12	3.66359
14	6277313	6277644	332	6277551	11	4.44564
14	6782811	6783521	711	6783302	8	4.19779
14	6892518	6892882	365	6892672	8	6.50668
14	6920918	6921444	527	6921169	7	3.09081
14	6984533	6985169	637	6984541	9	3.19498
14	7015805	7016131	327	7015932	8	5.93643
14	8654207	8654589	383	8654354	10	5.80609
14	8909491	8909875	385	8909539	7	5.47978
14	9359612	9360107	496	9359985	5	3.58241
14	9509196	9509506	311	9509226	7	3.45771

14	9512600	9512945	346	9512714	6	4.25501
14	9699943	9700296	354	9700080	9	5.37431
14	10303441	10303766	326	10303576	8	5.71006
14	11092520	11092896	377	11092607	6	3.98315
14	11492566	11493285	720	11492878	6	4.16086
14	13203896	13204258	363	13204063	7	5.47978
14	13657065	13657413	349	13657286	7	3.92737
14	14265374	14265686	313	14265661	6	3.3206
14	14639129	14639473	345	14639283	9	4.98189
14	17614815	17615116	302	17614964	6	3.79171
14	19416895	19418146	1252	19417280	7674	398.85123
14	19418541	19418953	413	19418856	3828	83.72426
14	19662526	19662868	343	19662566	6	3.79171
14	26723214	26723586	373	26723235	8	5.56774
14	26768499	26768848	350	26768656	8	6.50668
14	26913115	26913465	351	26913339	6	4.50291
14	28910242	28910602	361	28910518	6	3.89915
14	32848204	32848507	304	32848369	7	5.47978
14	39198627	39198962	336	39198800	8	6.50668
14	39543281	39543704	424	39543556	6	4.50291
14	39567342	39567685	344	39567465	7	5.47978
14	40235785	40236102	318	40235857	6	3.79171
14	41447376	41447749	374	41447651	14	5.60303
14	41635767	41636079	313	41635913	10	3.80233
14	41657268	41657723	456	41657643	9	4.76787
14	41763727	41764035	309	41763831	11	6.66709
14	41911292	41911610	319	41911424	8	4.19779
14	41966372	41966744	373	41966668	8	5.3496
14	42212357	42212656	300	42212407	10	4.46113
14	42215977	42216426	450	42216008	12	5.60636
14	42981142	42981846	705	42981689	7	5.19265
14	43616074	43616465	392	43616341	10	5.80609
14	43696336	43696650	315	43696567	7	3.26685
14	45592747	45593107	361	45593001	7	4.65491
14	46180774	46181090	317	46180890	6	4.50291
14	49766377	49766733	357	49766493	6	4.50291
14	49826597	49826898	302	49826794	6	4.50291
14	49888021	49888532	512	49888093	6	4.50291
14	50420204	50420621	418	50420517	8	6.50668
14	52219142	52219447	306	52219317	5	3.07446
14	53013828	53014193	366	53014082	5	3.58241
14	63113000	63113323	324	63113034	5	3.1094
14	65181656	65182033	378	65181876	7	5.47978
14	74100143	74100470	328	74100156	7	3.76048
14	77755816	77756169	354	77755974	5	3.58241
14	80842530	80842832	303	80842745	7	3.45771
14	81383074	81383401	328	81383082	5	3.58241
14	83847158	83847602	445	83847283	6	4.50291

14	84111099	84111421	323	84111333	6	4.20747
14	84600063	84600410	348	84600176	5	3.58241
14	84980412	84980735	324	84980424	6	3.02469
14	85352116	85352465	350	85352148	7	5.47978
14	86726836	86727156	321	86727154	6	4.02633
14	87191890	87192492	603	87192432	7	3.45771
14	87265659	87266022	364	87265838	6	4.07032
14	87701793	87702117	325	87701940	6	3.79171
14	89088911	89089278	368	89088969	6	4.50291
14	90064457	90064764	308	90064597	7	5.36479
14	90919153	90919653	501	90919401	7	5.47978
14	91382438	91382793	356	91382661	8	5.56774
14	91563231	91563647	417	91563596	7	5.47978
14	92594525	92595230	706	92594656	6	4.50291
14	93325935	93326280	346	93326013	6	4.50291
14	93366170	93366532	363	93366459	6	4.50291
14	94659508	94659831	324	94659652	6	4.50291
14	95466767	95467067	301	95466941	6	4.50291
14	95547662	95547982	321	95547788	6	4.50291
14	96669770	96670103	334	96669946	6	4.50291
14	96935649	96935959	311	96935776	8	6.50668
14	97151434	97151750	317	97151667	8	6.50668
14	97787126	97787451	326	97787180	5	3.58241
14	97788767	97789076	310	97789038	6	3.3206
14	97954977	97955276	300	97955147	7	3.45771
14	98609627	98610019	393	98609738	7	4.87731
14	98699052	98699375	324	98699368	6	3.79171
14	100681640	100681942	303	100681640	5	3.58241
14	103464341	103464744	404	103464481	22	20.54471
14	105505784	105506096	313	105505881	6	3.89915
14	105539779	105540117	339	105539929	8	5.93643
14	105551121	105551420	300	105551123	6	3.77863
14	105763879	105764200	322	105763913	7	3.96214
14	106768692	106769006	315	106768821	6	4.50291
14	107602229	107602772	544	107602465	7	5.47978
14	110996792	110997112	321	110996928	6	3.79171
14	119420098	119420435	338	119420117	6	4.50291
14	119480766	119481122	357	119480831	7	5.47978
14	122706506	122707092	587	122707027	7	5.47978
14	123875435	123875765	331	123875478	6	3.79171
14	124801590	124802106	517	124802075	31	37.9639
15	3113099	3113478	380	3113338	5	3.58241
15	4586855	4587201	347	4586928	10	7.52331
15	6593724	6594087	364	6593823	9	6.80986
15	7290679	7290989	311	7290944	5	3.58241
15	8076478	8076876	399	8076758	9	3.94891
15	9440166	9440484	319	9440228	5	3.33356
15	9845580	9846083	504	9845738	6	4.50291

15	10366092	10366422	331	10366247	6	4.50291
15	11881182	11881486	305	11881285	7	4.50662
15	13273271	13273814	544	13273286	6	3.79171
15	14301462	14301773	312	14301496	5	3.41441
15	14425051	14425563	513	14425473	6	3.66421
15	14638776	14639163	388	14639106	8	5.39858
15	15045909	15046437	529	15046186	6	3.98315
15	15393622	15394183	562	15393972	7	5.47978
15	16157885	16158212	328	16157958	6	4.50291
15	16623221	16623584	364	16623409	7	5.30623
15	16945623	16945943	321	16945831	6	4.16086
15	17165215	17165584	370	17165544	6	4.02633
15	18186023	18186340	318	18186290	7	4.97847
15	18564171	18564547	377	18564399	6	4.50291
15	19383171	19383503	333	19383199	8	3.28682
15	19674565	19674890	326	19674854	6	4.50291
15	19900810	19901272	463	19900909	6	3.79171
15	20477212	20477570	359	20477555	6	3.79171
15	21593162	21593520	359	21593165	6	3.02469
15	21599892	21600204	313	21600065	6	4.50291
15	21960001	21960645	645	21960587	7	3.45771
15	22256640	22257040	401	22256936	9	5.82058
15	22422495	22423026	532	22422716	9	6.33515
15	22713346	22713671	326	22713669	6	3.77863
15	22801524	22802527	1004	22802056	7	4.50662
15	22967336	22967686	351	22967555	7	4.73225
15	24998189	24998495	307	24998224	5	3.58241
15	25130698	25131043	346	25130833	7	4.97847
15	26582487	26582912	426	26582885	7	3.45771
15	27692712	27693038	327	27692918	5	3.58241
15	28804087	28804567	481	28804205	6	3.79171
15	28987934	28988264	331	28988072	9	6.17252
15	29786608	29787259	652	29786738	9	6.33515
15	30463008	30463318	311	30463139	7	5.47978
15	31861737	31862048	312	31861772	5	3.14499
15	33562746	33563051	306	33562850	13	4.22859
15	33765692	33765993	302	33765802	7	3.45771
15	33832293	33832848	556	33832782	14	4.98112
15	34501137	34501449	313	34501334	9	4.98189
15	35089113	35089415	303	35089406	8	3.13864
15	35777683	35777988	306	35777912	6	3.89915
15	39860915	39861249	335	39861100	7	4.50662
15	42213853	42214211	359	42213964	5	3.29435
15	42237148	42237508	361	42237489	7	3.99741
15	44242344	44242663	320	44242452	7	5.30623
15	44790991	44791385	395	44791240	8	5.99565
15	44875567	44875874	308	44875810	6	3.52023
15	45438280	45438607	328	45438490	7	5.30623

15	46498911	46499229	319	46498924	7	3.45771
15	46612862	46613298	437	46613017	6	4.11516
15	47545998	47546300	303	47546236	6	4.50291
15	48892857	48893255	399	48893072	7	4.92742
15	48975706	48976098	393	48975753	6	3.62733
15	49011063	49011394	332	49011087	6	4.07032
15	51231951	51232285	335	51231979	9	4.44134
15	52526905	52527208	304	52527050	8	4.94122
15	54676453	54676811	359	54676773	7	4.92742
15	54750762	54751121	360	54750965	7	3.45771
15	55293431	55293742	312	55293671	8	3.28682
15	56068264	56068593	330	56068421	5	3.1094
15	65280879	65281178	300	65281131	7	4.50662
15	65522061	65522439	379	65522172	6	4.50291
15	66077715	66078026	312	66077958	6	4.16086
15	66841816	66842126	311	66841939	8	4.19779
15	67966780	67967109	330	67966985	7	4.22
15	68637478	68637825	348	68637661	7	5.47978
15	68855857	68856222	366	68855912	6	4.50291
15	69401090	69401425	336	69401314	8	5.56774
15	70342393	70342706	314	70342551	6	4.40359
15	70360072	70360408	337	70360196	5	3.33356
15	70832040	70832380	341	70832283	7	4.87731
15	71105708	71106243	536	71105952	7	3.45771
15	71109939	71110269	331	71109979	6	4.50291
15	71999678	72000018	341	71999715	6	3.79171
15	72088258	72088574	317	72088277	7	5.03049
15	72519574	72519961	388	72519754	7	5.47978
15	72568481	72568794	314	72568599	7	5.42458
15	73084728	73085048	321	73084818	8	6.50668
15	73399105	73399432	328	73399389	9	4.76787
15	82271119	82271419	301	82271307	7	4.65491
15	86248515	86248887	373	86248750	6	4.50291
15	86425363	86425685	323	86425451	6	3.98315
15	87254939	87255275	337	87255007	5	3.58241
15	87429744	87430070	327	87429947	5	3.58241
15	89327192	89327815	624	89327316	6	4.11516
15	91252483	91252959	477	91252818	10	5.41652
15	91733519	91733841	323	91733565	5	3.41441
15	94903137	94903528	392	94903370	10	8.69114
15	97482957	97483288	332	97483000	9	4.98189
15	103911880	103912230	351	103912035	8	6.50668
16	3638288	3638610	323	3638395	6	4.50291
16	3870242	3870602	361	3870352	5	3.49869
16	3887690	3888032	343	3887810	5	3.14499
16	6908130	6908680	551	6908483	5	3.58241
16	8286041	8286438	398	8286410	6	4.50291
16	9064232	9064552	321	9064275	5	3.58241

16	11432049	11432376	328	11432250	8	5.39858
16	12397676	12397976	301	12397814	6	4.50291
16	19666568	19667061	494	19666718	5	3.58241
16	20847221	20847524	304	20847444	7	5.47978
16	23024166	23024506	341	23024426	5	3.1094
16	25557651	25557969	319	25557676	6	4.07032
16	25566630	25567034	405	25566673	7	5.47978
16	25992444	25992775	332	25992578	9	5.91748
16	26258362	26258686	325	26258530	8	6.50668
16	26855676	26856036	361	26855959	7	4.77975
16	27213449	27213776	328	27213476	8	3.28682
16	27603148	27603485	338	27603332	7	4.65491
16	37405233	37405588	356	37405492	5	3.37357
16	38912409	38912738	330	38912682	6	4.50291
16	39442398	39442724	327	39442527	7	5.47978
16	40241166	40241481	316	40241288	5	3.58241
16	40802086	40802398	313	40802101	6	3.66421
16	41069998	41070308	311	41070258	6	4.35303
16	42329144	42329578	435	42329231	6	3.79171
16	45375575	45375935	361	45375831	6	3.79171
16	46219953	46220262	310	46219961	5	3.41441
16	46220841	46221180	340	46220965	6	4.07032
16	47688299	47688646	348	47688450	7	5.47978
16	48495649	48495965	317	48495653	7	3.45771
16	48675917	48676235	319	48676061	7	5.30623
16	48865988	48866343	356	48866126	7	5.47978
16	50344480	50344868	389	50344645	10	7.31225
16	50667874	50668178	305	50667976	6	4.02633
16	51026197	51026510	314	51026297	7	4.68558
16	51315923	51316234	312	51315977	5	3.58241
16	51413857	51414170	314	51414154	6	4.50291
16	51473489	51473826	338	51473570	5	3.58241
16	51604785	51605218	434	51605038	5	3.58241
16	52788138	52788517	380	52788258	8	6.50668
16	53124730	53125073	344	53124921	7	5.47978
16	53484795	53485119	325	53484912	5	3.18126
16	53561161	53561510	350	53561251	5	3.58241
16	53690794	53691129	336	53690976	8	5.56774
16	57590329	57590651	323	57590557	7	4.97847
16	58825003	58825349	347	58825089	8	6.05599
16	58858988	58859321	334	58859117	6	4.45522
16	58982684	58983014	331	58982782	6	4.50291
16	59007550	59007882	333	59007867	6	3.79171
16	59124832	59125210	379	59125041	8	6.18019
16	60595179	60595505	327	60595411	7	4.65491
16	61516609	61516941	333	61516771	6	4.50291
16	61699457	61699778	322	61699499	6	3.79171
16	62705640	62706045	406	62705868	8	4.19779

16	62883669	62884018	350	62883913	5	3.58241
16	63110697	63111041	345	63110773	6	3.79171
16	63407754	63408079	326	63407836	8	5.39858
16	63881333	63881709	377	63881555	7	4.92742
16	64071739	64072140	402	64071765	6	3.79171
16	64474998	64475332	335	64475277	9	6.33515
16	64481816	64482154	339	64482045	6	4.50291
16	65157596	65157905	310	65157628	6	4.45522
16	65442742	65443091	350	65442789	6	4.25501
16	65875309	65875654	346	65875613	5	3.41441
16	66410390	66410740	351	66410568	6	4.50291
16	69349302	69349624	323	69349622	6	3.02469
16	69420163	69420477	315	69420454	6	3.98315
16	69963863	69964178	316	69963881	5	3.58241
16	70231649	70231968	320	70231739	5	3.37357
16	70350958	70351287	330	70350987	7	4.10626
16	72743544	72743873	330	72743569	5	3.58241
16	72830031	72830364	334	72830334	6	4.50291
16	73999934	74000381	448	74000343	5	3.21823
16	79048945	79049257	313	79049041	6	4.50291
16	79422215	79422572	358	79422379	7	4.65491
16	80821005	80821310	306	80821072	7	3.45771
16	81226430	81226811	382	81226519	6	4.50291
16	81867324	81867663	340	81867363	7	4.87731
16	82344644	82345105	462	82344759	6	4.50291
16	82579628	82580047	420	82579969	6	4.50291
16	83221581	83221953	373	83221655	7	5.0835
16	83620510	83621033	524	83620812	8	5.87829
16	84385184	84385551	368	84385277	11	8.55855
16	84670281	84670814	534	84670529	7	5.30623
16	85536818	85537133	316	85536985	5	3.1094
16	85789034	85789452	419	85789075	6	3.66421
16	88166560	88166971	412	88166889	5	3.58241
16	93620366	93620682	317	93620397	5	3.58241
16	94924303	94924717	415	94924630	6	3.79171
17	3788235	3788594	360	3788563	5	3.58241
17	6485772	6486089	318	6485997	6	3.3206
17	6921086	6921446	361	6921292	8	3.28682
17	7103730	7104057	328	7103955	6	4.02633
17	8382018	8382376	359	8382355	6	3.79171
17	8936775	8937141	367	8936863	8	5.56774
17	9030807	9031193	387	9031082	29	29.19179
17	13400283	13400633	351	13400369	6	3.35262
17	13589918	13590577	660	13590476	87	7.96078
17	17991975	17992604	630	17992318	6	4.35303
17	18036340	18036711	372	18036685	6	4.25501
17	18695176	18696105	930	18695846	7	5.47978
17	18825241	18825594	354	18825261	5	3.07446

17	19365241	19365855	615	19365443	7	5.42458
17	20192507	20192833	327	20192755	9	6.74692
17	20445837	20446192	356	20445980	6	4.50291
17	20475811	20476134	324	20476104	9	3.94891
17	20510741	20511159	419	20511035	9	6.52524
17	22634372	22634703	332	22634551	6	3.79171
17	22699710	22700041	332	22699938	8	6.50668
17	22793566	22793894	329	22793851	7	4.50662
17	22856680	22857271	592	22856824	5	3.58241
17	33016996	33017338	343	33017279	6	3.79171
17	36427208	36427757	550	36427663	6	4.50291
17	36438602	36438980	379	36438721	7	3.45771
17	36552734	36553117	384	36552924	7	5.47978
17	37337716	37338042	327	37337856	7	4.50662
17	37463313	37463621	309	37463517	6	4.35303
17	37803292	37803618	327	37803495	6	3.66421
17	37907407	37907819	413	37907564	7	5.24887
17	38210358	38210699	342	38210522	8	5.56774
17	38828816	38829213	398	38829144	9	3.19498
17	39037647	39037989	343	39037936	6	3.79171
17	39740280	39740591	312	39740282	5	3.58241
17	39846657	39848022	1366	39847077	440	168.01476
17	40443691	40444008	318	40443900	7	5.19265
17	40983877	40984196	320	40984046	8	6.50668
17	41329231	41329539	309	41329535	6	4.07032
17	41942774	41943228	455	41942970	7	4.18152
17	42431609	42431944	336	42431627	7	4.5946
17	43407311	43407649	339	43407480	8	4.19779
17	44916616	44916942	327	44916724	8	5.56774
17	49941009	49941470	462	49941350	31	37.9639
17	50076457	50076975	519	50076626	8	5.93643
17	51350087	51350423	337	51350199	7	5.47978
17	52412766	52413129	364	52412826	6	4.20747
17	53099088	53099642	555	53099163	7	4.65491
17	53181957	53182471	515	53182031	5	3.58241
17	55628365	55628666	302	55628451	5	3.58241
17	55753275	55753670	396	55753337	5	3.07446
17	57435616	57435971	356	57435901	7	5.42458
17	57543747	57544050	304	57543870	9	7.28429
17	58364099	58364460	362	58364182	5	3.58241
17	58742967	58743323	357	58743262	5	3.58241
17	59222004	59222354	351	59222022	7	4.50662
17	60087469	60087776	308	60087602	7	5.47978
17	60112953	60113320	368	60113295	8	3.28682
17	60986009	60986523	515	60986061	6	4.40359
17	61285073	61285453	381	61285310	7	4.87731
17	62906864	62907186	323	62907031	6	3.66421
17	62960216	62960556	341	62960327	6	3.41811

17	63217080	63217381	302	63217200	7	4.06945
17	63714116	63714438	323	63714225	8	4.40106
17	65303220	65303829	610	65303759	7	5.36479
17	65530511	65530830	320	65530578	5	3.21823
17	67176500	67177209	710	67177058	6	3.79171
17	68047141	68047458	318	68047251	5	3.21823
17	68491179	68491550	372	68491474	5	3.58241
17	69964318	69964644	327	69964356	7	5.0835
17	69991766	69992086	321	69991836	10	7.07319
17	70112517	70112846	330	70112539	5	3.58241
17	70550442	70550923	482	70550750	7	5.03049
17	72225969	72226348	380	72226125	12	7.56208
17	76761913	76762295	383	76762011	7	5.47978
17	76902559	76903101	543	76902851	8	5.56774
17	77588359	77588757	399	77588693	9	7.57852
17	77922470	77922828	359	77922505	5	3.5422
17	79087339	79088351	1013	79088237	11	8.60648
17	80730359	80730673	315	80730459	6	4.11516
17	81436440	81436973	534	81436446	6	3.3206
17	82106621	82106942	322	82106774	7	4.82809
17	82230184	82230550	367	82230224	6	4.50291
17	82338256	82338621	366	82338345	5	3.58241
17	82363617	82363973	357	82363670	6	4.50291
17	82387171	82387489	319	82387365	5	3.14499
17	82797010	82797317	308	82797012	6	3.79171
17	82935970	82936340	371	82936282	6	4.50291
17	86177283	86177844	562	86177432	6	4.35303
17	89080361	89080719	359	89080425	6	4.25501
17	89334135	89334618	484	89334418	6	4.50291
17	89453535	89453843	309	89453558	7	4.10626
17	89827107	89827580	474	89827420	6	3.79171
17	90329498	90329805	308	90329627	7	4.25906
17	90497337	90497654	318	90497465	5	3.58241
17	90538203	90538597	395	90538266	7	4.97847
17	91127911	91128486	576	91128372	6	4.02633
17	92218006	92218330	325	92218048	7	4.77975
17	93346871	93347254	384	93346914	7	3.72842
17	93662483	93663017	535	93662697	7	3.45771
17	93829925	93830238	314	93830143	5	3.37357
17	93912688	93913022	335	93912856	8	6.50668
18	3132380	3132697	318	3132566	8	6.50668
18	14913685	14914141	457	14913941	29	30.86135
18	17466995	17467308	314	17467025	8	5.39858
18	17542226	17542557	332	17542280	6	3.79171
18	18831280	18831596	317	18831451	11	8.90566
18	24677180	24677484	305	24677334	18	18.73404
18	26521116	26521560	445	26521414	7	5.47978
18	29011152	29011465	314	29011202	6	4.07032

18	29243750	29244246	497	29244066	6	3.98315
18	29405013	29405606	594	29405425	7	5.47978
18	37632455	37632833	379	37632549	6	4.50291
18	40780283	40780592	310	40780407	6	4.50291
18	42558015	42558733	719	42558440	51	60.05621
18	43651258	43651588	331	43651266	5	3.4561
18	44278317	44278622	306	44278602	6	3.66421
18	44343209	44343576	368	44343338	10	3.80233
18	44701649	44701985	337	44701782	7	3.99741
18	47390246	47390596	351	47390308	7	4.65491
18	47450374	47450680	307	47450468	6	4.50291
18	59510337	59510648	312	59510423	9	6.52524
18	62621270	62621656	387	62621464	8	6.50668
18	75887724	75888025	302	75887808	5	3.4561
18	78483613	78483953	341	78483763	8	5.39858
18	83262030	83262359	330	83262069	7	5.47978
18	85872631	85873095	465	85872768	5	3.58241
18	86533102	86533456	355	86533245	6	4.50291
18	88375058	88375371	314	88375271	6	4.50291
18	90246994	90247323	330	90247116	6	4.50291
19	5444470	5444938	469	5444490	5	3.58241
19	7946256	7946557	302	7946390	7	4.65491
19	8056986	8057364	379	8057232	6	3.8181
19	8246733	8247068	336	8246746	8	5.39858
19	11157389	11157724	336	11157405	5	3.49869
19	11250285	11250602	318	11250334	7	5.47978
19	12098134	12098499	366	12098184	7	5.47978
19	12822079	12822385	307	12822222	6	4.50291
19	17598153	17598472	320	17598359	7	5.47978
19	17799242	17799737	496	17799434	6	4.50291
19	18008133	18008509	377	18008167	5	3.58241
19	18510777	18511182	406	18510812	6	4.50291
19	20559164	20559566	403	20559356	7	5.19265
19	22215342	22215648	307	22215391	6	4.40359
19	23470037	23470368	332	23470061	5	3.58241
19	23689928	23690325	398	23690212	6	3.79171
19	27132420	27132742	323	27132582	6	3.79171
19	27824010	27824363	354	27824088	6	4.35303
19	29444748	29445082	335	29445071	7	3.76048
19	31062865	31063189	325	31062879	5	3.58241
19	33722882	33723212	331	33723025	8	5.93643
19	38474749	38475056	308	38474888	6	4.35303
19	39641239	39641561	323	39641396	6	4.50291
19	39838988	39839321	334	39839194	6	4.50291
19	40508441	40508855	415	40508726	107	169.49567
19	43417588	43418100	513	43417957	6	3.89915
19	50231119	50231488	370	50231195	7	5.47978
19	50787004	50787404	401	50787235	6	4.02633

19	53731901	53732235	335	53732198	7	3.76048
19	55139426	55139735	310	55139645	6	4.50291
19	56243553	56243853	301	56243699	6	4.50291
19	58634808	58635138	331	58635012	6	4.07032
19	61293924	61294228	305	61294076	6	4.50291
19	61314492	61314879	388	61314819	6	3.79171
2	4208018	4208317	300	4208176	8	6.50668
2	4748271	4748662	392	4748540	5	3.41441
2	6889044	6889351	308	6889208	6	4.50291
2	8598995	8599464	470	8599317	6	4.50291
2	8655377	8655788	412	8655449	7	5.47978
2	10679395	10679746	352	10679651	5	3.58241
2	13926685	13927052	368	13926845	5	3.58241
2	14939506	14939881	376	14939572	7	5.47978
2	19481157	19481494	338	19481222	7	5.47978
2	22107228	22107546	319	22107241	6	3.79171
2	23145900	23146213	314	23145910	7	3.1897
2	23276349	23276748	400	23276660	6	4.50291
2	31706100	31706479	380	31706296	5	3.41441
2	34516454	34516868	415	34516605	8	6.50668
2	37040925	37041249	325	37040976	5	3.58241
2	39779435	39779816	382	39779581	9	7.57852
2	40551888	40552218	331	40552016	6	4.25501
2	42209962	42210369	408	42210012	7	4.65491
2	42678860	42679231	372	42678887	5	3.58241
2	43360341	43360845	505	43360500	7	5.47978
2	44072643	44073144	502	44072908	7	5.47978
2	44722114	44722424	311	44722156	7	5.19265
2	45796554	45797089	536	45796569	6	4.50291
2	46175295	46175635	341	46175342	6	4.07032
2	46192741	46193070	330	46192807	7	5.47978
2	46570742	46571093	352	46570868	7	5.47978
2	53226482	53226799	318	53226608	8	4.19779
2	53312868	53313245	378	53312911	6	4.50291
2	53664296	53664657	362	53664441	9	4.98189
2	55122690	55123023	334	55122728	6	4.50291
2	56162690	56163037	348	56162806	7	5.24887
2	56771793	56772159	367	56772018	6	4.50291
2	57296837	57297137	301	57296885	6	4.30352
2	62547426	62547949	524	62547898	7	5.19265
2	63734199	63734581	383	63734489	5	3.04015
2	65676896	65677202	307	65677198	7	3.45771
2	65982410	65982753	344	65982555	5	3.58241
2	66010223	66010551	329	66010252	6	3.73984
2	66312787	66313121	335	66312900	8	5.56774
2	66584181	66584485	305	66584270	11	8.25921
2	66808854	66809218	365	66809103	7	5.0835
2	67716757	67717060	304	67716823	5	3.58241

2	68940324	68940712	389	68940497	6	3.85827
2	72699736	72700229	494	72700084	6	3.66421
2	75534886	75535260	375	75534894	6	3.79171
2	77153826	77154194	369	77154119	7	4.97847
2	78521995	78522368	374	78522199	9	6.52524
2	79902583	79902906	324	79902623	7	4.97847
2	79904539	79904935	397	79904705	9	6.68507
2	82388828	82389245	418	82389049	7	5.47978
2	83062805	83063116	312	83062894	8	4.77631
2	83851604	83851973	370	83851870	5	3.29435
2	84043496	84043824	329	84043527	7	5.19265
2	87073208	87073528	321	87073363	5	3.58241
2	87691927	87692263	337	87692200	6	4.50291
2	87818060	87818410	351	87818063	6	3.79171
2	88509948	88510293	346	88510014	5	3.58241
2	88562193	88562496	304	88562218	6	4.45522
2	89561436	89561764	329	89561440	6	4.20747
2	89678306	89678723	418	89678341	5	3.58241
2	89812032	89812646	615	89812158	6	4.50291
2	89983304	89983817	514	89983521	6	4.50291
2	93458231	93458566	336	93458500	7	4.10626
2	95290844	95291176	333	95290889	5	3.58241
2	95379720	95380080	361	95379839	6	4.30352
2	95580791	95581309	519	95580898	7	4.65491
2	96511235	96511670	436	96511547	6	4.50291
2	97216287	97216611	325	97216445	7	4.68558
2	97907192	97907721	530	97907418	6	3.79171
2	97957147	97957803	657	97957584	6	4.50291
2	98459297	98459666	370	98459542	6	3.79171
2	98666699	98667220	522	98666857	27881	802.77637
2	100454011	100454347	337	100454237	6	4.50291
2	100950200	100950506	307	100950279	6	4.50291
2	100957753	100958108	356	100957976	8	5.56774
2	104088727	104089065	339	104088738	8	3.28682
2	104296724	104297165	442	104296851	5	3.58241
2	104424423	104424782	360	104424662	6	4.02633
2	106325748	106326050	303	106325959	6	4.50291
2	107615865	107616179	315	107615969	7	5.47978
2	108560870	108561207	338	108561084	6	3.94077
2	111169851	111170186	336	111169993	6	4.50291
2	111703383	111703735	353	111703394	5	3.58241
2	113234782	113235148	367	113235118	6	4.50291
2	114212204	114212561	358	114212435	7	5.47978
2	122013593	122013922	330	122013726	10	6.58426
2	122919766	122920094	329	122919911	7	4.97847
2	122987775	122988082	308	122987980	7	4.65491
2	125112588	125113029	442	125112765	7	3.45771
2	125706502	125706827	326	125706566	7	5.03049

2	128244795	128245110	316	128245024	6	4.50291
2	130240769	130241435	667	130240784	6	3.66421
2	130759797	130760124	328	130759920	6	4.45522
2	132503645	132504430	786	132504147	6	4.50291
2	133081271	133081669	399	133081341	5	3.58241
2	134023049	134023409	361	134023225	7	5.47978
2	136866697	136867098	402	136867002	9	6.52524
2	137136982	137137340	359	137137165	5	3.58241
2	137943014	137943357	344	137943027	6	3.98315
2	138249245	138249627	383	138249330	8	4.19779
2	138729353	138729731	379	138729627	7	5.47978
2	139272704	139273022	319	139272890	7	5.42458
2	139472569	139472897	329	139472683	6	4.45522
2	140232463	140232792	330	140232659	5	3.33356
2	140290946	140291484	539	140291205	9	5.91748
2	141260850	141261162	313	141261064	8	5.56774
2	142575645	142575953	309	142575828	6	4.50291
2	143769906	143770426	521	143770233	6	3.79171
2	148103970	148104277	308	148104033	6	4.50291
2	150047285	150047677	393	150047608	7	5.47978
2	153236650	153237028	379	153236735	8	6.50668
2	154296002	154296336	335	154296098	6	4.02633
2	155202985	155203333	349	155203211	9	7.57852
2	159786784	159787230	447	159787147	7	4.65491
2	160776013	160776356	344	160776304	7	5.47978
2	163350617	163350949	333	163350810	7	5.47978
2	170606263	170606830	568	170606286	6	3.79171
2	173835342	173835652	311	173835451	6	4.50291
2	178006762	178007097	336	178006765	6	3.3206
2	178395797	178396170	374	178395902	8	4.98393
3	4007593	4007908	316	4007757	6	4.50291
3	4207903	4208368	466	4208111	5	3.07446
3	4222554	4222904	351	4222772	6	3.89915
3	4825568	4825909	342	4825753	5	3.41441
3	6485508	6485841	334	6485811	6	4.40359
3	6972902	6973268	367	6973161	6	4.50291
3	8567164	8567486	323	8567387	6	4.50291
3	9225090	9225449	360	9225256	9	6.52524
3	11620776	11621087	312	11620886	7	5.0835
3	11714847	11715168	322	11714862	5	3.58241
3	12416608	12416922	315	12416683	5	3.58241
3	13918505	13918897	393	13918822	7	4.65491
3	15036287	15036625	339	15036365	6	3.85827
3	16603257	16603564	308	16603395	7	4.97847
3	17976586	17976904	319	17976758	8	6.50668
3	19866643	19867074	432	19866849	38	44.85554
3	23278707	23279066	360	23278724	5	3.58241
3	24772059	24772378	320	24772078	5	3.21823

3	25157351	25157677	327	25157411	8	6.05599
3	29715993	29716620	628	29716151	8	6.50668
3	34123472	34123805	334	34123775	6	4.50291
3	34247789	34248144	356	34248009	5	3.58241
3	35138185	35138485	301	35138396	6	4.50291
3	36025042	36025359	318	36025166	9	6.93917
3	44363058	44363363	306	44363132	7	3.76048
3	44464197	44464553	357	44464453	6	3.85827
3	45171049	45171418	370	45171337	7	4.37991
3	45357644	45358017	374	45357713	6	4.02633
3	45665274	45665673	400	45665392	6	4.30352
3	45694242	45694596	355	45694562	7	5.30623
3	46088784	46089158	375	46088943	8	6.50668
3	46590812	46591122	311	46590954	5	3.4561
3	46684373	46684713	341	46684676	6	3.66421
3	47185666	47186007	342	47185682	7	4.50662
3	49732940	49733287	348	49732994	8	5.56774
3	50653675	50653976	302	50653790	7	3.45771
3	50950317	50950675	359	50950518	6	4.50291
3	52359120	52359424	305	52359318	7	5.47978
3	53100093	53100434	342	53100297	6	4.02633
3	53513898	53514252	355	53514030	6	4.50291
3	56297421	56297764	344	56297564	6	4.50291
3	57084453	57084764	312	57084575	6	4.20747
3	59265852	59266325	474	59266052	6	4.50291
3	59463118	59463432	315	59463145	6	4.50291
3	59483130	59483462	333	59483306	7	3.45771
3	61231105	61231479	375	61231348	7	5.36479
3	62208976	62209313	338	62209151	7	4.92742
3	63661283	63661631	349	63661333	5	3.58241
3	63764275	63764630	356	63764490	7	3.45771
3	67336841	67337181	341	67336894	8	6.44394
3	67431186	67431500	315	67431285	6	4.20747
3	68547932	68548234	303	68548024	8	6.50668
3	71904937	71905268	332	71905023	7	4.73225
3	72010109	72010412	304	72010216	7	5.47978
3	72017834	72018140	307	72018057	8	5.25394
3	72226255	72226564	310	72226461	8	6.30939
3	73257279	73257626	348	73257380	6	3.79171
3	74583961	74584275	315	74584033	6	4.50291
3	75338260	75338605	346	75338505	5	3.58241
3	76037874	76038281	408	76037979	5	3.33356
3	76272670	76273043	374	76272918	7	5.42458
3	76333631	76334004	374	76333754	6	4.07032
3	76488097	76488421	325	76488183	5	3.58241
3	77041779	77042099	321	77042013	6	3.85827
3	77234667	77234966	300	77234912	7	5.42458
3	81485669	81486052	384	81485998	8	6.18019

3	81631312	81631652	341	81631406	6	3.48566
3	89705166	89705471	306	89705286	6	4.50291
3	90220238	90220610	373	90220359	9	7.57852
3	91181704	91182012	309	91181986	6	4.50291
3	91459240	91459561	322	91459297	6	4.20747
3	91984886	91985254	369	91985004	6	4.50291
3	92313318	92313736	419	92313635	8	4.19779
3	92446149	92446448	300	92446321	6	4.02633
3	92785366	92785712	347	92785672	5	3.58241
3	92811685	92812037	353	92811951	7	5.47978
3	93187032	93187537	506	93187103	5	3.58241
3	93315669	93315990	322	93315798	7	4.65491
3	94080916	94081237	322	94081004	6	3.79171
3	94215680	94216425	746	94215914	9	6.33515
3	98275889	98276213	325	98275934	6	4.50291
3	98592947	98593390	444	98593365	6	3.94077
3	99081140	99081515	376	99081374	7	5.47978
3	99906099	99906417	319	99906342	9	6.93917
3	99959399	99959765	367	99959687	5	3.58241
3	106315491	106315807	317	106315666	6	4.45522
3	109141872	109142298	427	109142198	6	4.35303
3	110681289	110681663	375	110681414	9	7.35743
3	111761977	111762304	328	111762148	7	5.47978
3	113351526	113351861	336	113351624	5	3.58241
3	113788520	113788821	302	113788718	7	5.47978
3	116763990	116764317	328	116764242	6	3.79171
3	117651762	117652106	345	117651821	5	3.58241
3	118096450	118096842	393	118096678	8	5.56774
3	118329598	118329909	312	118329695	5	3.58241
3	118719591	118719892	302	118719717	7	5.47978
3	120108186	120108506	321	120108248	8	6.50668
3	120280895	120281256	362	120280970	5	3.58241
3	121812616	121812922	307	121812643	7	3.76048
3	125338814	125339381	568	125338933	7	4.50662
3	125518525	125518999	475	125518584	6	4.50291
3	125614286	125614753	468	125614581	8	4.19779
3	127222239	127222584	346	127222377	5	3.58241
3	132850781	132851080	300	132851006	7	5.47978
3	134068275	134068607	333	134068287	5	3.58241
3	134451194	134451527	334	134451443	6	4.50291
3	134853168	134853555	388	134853502	6	4.50291
3	135193120	135193483	364	135193320	7	5.19265
3	139385287	139385676	390	139385330	7	4.65491
3	139634085	139634547	463	139634085	8	3.28682
3	140539883	140540232	350	140540177	5	3.07446
3	140745324	140745665	342	140745562	5	3.37357
3	141371632	141371958	327	141371910	6	3.22735
3	143195000	143195758	759	143195621	6	4.25501

3	144033739	144034069	331	144033956	9	4.98189
3	146075041	146075407	367	146075192	7	5.13754
3	148315396	148315762	367	148315423	5	3.14499
3	149575040	149575369	330	149575221	6	4.02633
3	150161201	150161538	338	150161474	7	5.13754
3	152101888	152102189	302	152101928	5	3.37357
3	152333987	152334289	303	152333989	5	3.58241
3	155173579	155173968	390	155173809	7	5.47978
3	155857632	155858013	382	155857934	8	6.50668
3	157801010	157801324	315	157801053	5	3.58241
3	158770858	158771182	325	158771067	6	4.50291
4	3130203	3130503	301	3130389	8	5.39858
4	3235468	3235803	336	3235574	10	4.63111
4	5394379	5394694	316	5394509	5	3.58241
4	5646411	5646796	386	5646535	10	8.69114
4	9937705	9938033	329	9937812	5	3.58241
4	10106053	10106436	384	10106254	7	3.45771
4	10393898	10394255	358	10394129	7	5.42458
4	13532048	13532385	338	13532317	5	3.21823
4	14399512	14399818	307	14399727	6	4.50291
4	16312826	16313137	312	16312901	5	3.58241
4	16320960	16321300	341	16321057	8	4.19779
4	17779968	17780317	350	17780249	8	5.56774
4	18714387	18714754	368	18714642	6	4.40359
4	19156467	19156768	302	19156474	7	5.47978
4	20064133	20064488	356	20064152	5	3.37357
4	20192887	20193237	351	20192947	7	5.47978
4	22485130	22485441	312	22485288	6	3.79171
4	24119050	24119358	309	24119262	7	4.97847
4	24196519	24196866	348	24196632	10	8.69114
4	25694510	25694827	318	25694706	6	4.50291
4	26244498	26244816	319	26244599	5	3.58241
4	27912680	27913068	389	27912830	5	3.58241
4	28272699	28273032	334	28272701	6	3.94077
4	29558188	29558542	355	29558388	7	4.82809
4	29746196	29746502	307	29746345	6	4.50291
4	30337422	30337746	325	30337526	9	5.37431
4	30935474	30935810	337	30935642	7	5.19265
4	31311937	31312525	589	31312218	7	4.77975
4	31478384	31478766	383	31478665	6	4.25501
4	32170159	32170503	345	32170202	6	3.79171
4	35841998	35842466	469	35842139	7	3.45771
4	39622831	39623151	321	39622837	5	3.58241
4	45234855	45235197	343	45234973	7	5.24887
4	47751663	47752029	367	47751736	5	3.58241
4	49962963	49963328	366	49963264	6	3.79171
4	50673134	50673474	341	50673281	7	5.47978
4	50977994	50978321	328	50978117	7	5.0835

4	59941879	59942199	321	59942187	6	4.50291
4	60295484	60295819	336	60295691	6	3.66421
4	61273647	61274020	374	61273670	5	3.58241
4	64521254	64521711	458	64521591	6	3.3206
4	66079399	66079700	302	66079527	7	5.47978
4	66191313	66191761	449	66191606	7	5.47978
4	66766559	66766884	326	66766734	5	3.41441
4	67377030	67377398	369	67377173	8	6.50668
4	69029109	69029460	352	69029253	6	4.35303
4	69568418	69568746	329	69568578	8	6.50668
4	70946786	70947192	407	70947124	8	6.50668
4	71023175	71023474	300	71023309	6	4.50291
4	71297080	71297445	366	71297250	8	6.11749
4	72845833	72846315	483	72846126	7	4.97847
4	75095840	75096152	313	75096018	6	4.50291
4	77017571	77017928	358	77017717	9	7.57852
4	77197772	77198073	302	77197858	8	6.24414
4	79384847	79385156	310	79384953	6	4.50291
4	79902162	79902537	376	79902320	8	6.50668
4	81145298	81145613	316	81145346	6	4.50291
4	81605185	81605550	366	81605203	8	5.56774
4	85052726	85053052	327	85052841	7	3.45771
4	85081215	85081531	317	85081384	7	3.45771
4	85689910	85690221	312	85690053	6	4.45522
4	85962657	85962999	343	85962772	7	4.97847
4	89736980	89737316	337	89737036	6	3.59105
4	90199377	90199694	318	90199573	6	4.07032
4	90563778	90564139	362	90563849	5	3.58241
4	91879389	91879729	341	91879556	8	5.56774
4	93777083	93777415	333	93777330	6	4.11516
4	96284862	96285189	328	96285136	5	3.29435
4	96352134	96352526	393	96352206	6	4.50291
4	98822432	98822736	305	98822566	7	5.47978
4	99594620	99594969	350	99594733	7	4.65491
4	103042911	103043501	591	103043473	6	3.89915
4	103325702	103326014	313	103325856	7	5.47978
4	103817812	103818140	329	103817972	7	5.47978
4	104534856	104535274	419	104534987	7	5.47978
4	110613735	110614122	388	110613951	8	6.50668
4	111911948	111912296	349	111912106	5	3.58241
4	115026216	115026590	375	115026278	5	3.58241
4	115573527	115573829	303	115573702	6	4.50291
4	126377306	126377618	313	126377317	7	4.339
4	127109560	127109992	433	127109571	5	3.58241
4	127564094	127564456	363	127564131	5	3.58241
4	136574947	136575322	376	136574980	7	4.10626
4	145493345	145493897	553	145493833	7	4.65491
4	145631337	145631668	332	145631627	6	4.35303

4	145876061	145876408	348	145876257	6	4.20747
4	146088464	146088792	329	146088682	7	3.79296
4	146165662	146166069	408	146165927	12	5.60636
4	146192831	146193192	362	146192977	11	6.62187
4	146443715	146444183	469	146443933	9	4.32007
4	146447278	146447745	468	146447511	9	3.94891
4	146476489	146476840	352	146476657	12	6.06634
4	146599027	146599909	883	146599044	9	3.19498
4	146716757	146717077	321	146716946	15	6.28915
4	147018099	147018417	319	147018307	10	5.80609
4	147158973	147159311	339	147159136	6	3.98315
4	147190612	147191397	786	147191315	11	6.446
4	147273376	147273682	307	147273563	7	3.72842
4	147379377	147379857	481	147379736	10	3.14799
4	147380949	147381372	424	147380990	11	3.71443
4	147478783	147479116	334	147478829	7	3.45771
4	147497751	147498298	548	147498027	10	6.19739
4	147672688	147673081	394	147673067	7	4.5946
5	5244159	5244466	308	5244302	6	3.66421
5	5453839	5454279	441	5453979	8	4.00857
5	6187520	6187909	390	6187560	7	3.45771
5	6525147	6525889	743	6525156	8	3.88945
5	6738934	6739272	339	6739073	10	5.80609
5	6886751	6887130	380	6886809	10	5.80609
5	7050830	7051139	310	7051052	7	4.42147
5	7173416	7173814	399	7173802	6	3.77863
5	7297917	7298239	323	7298078	7	5.47978
5	7519698	7520121	424	7519761	8	4.94122
5	7619775	7620099	325	7619791	6	3.79171
5	7678257	7678652	396	7678545	6	3.79171
5	7699705	7700080	376	7699856	8	6.05599
5	8876928	8877278	351	8877187	9	5.37431
5	9225340	9225718	379	9225697	7	4.06945
5	9484327	9484631	305	9484598	7	3.45771
5	9527839	9528247	409	9528128	5	3.58241
5	9738317	9738752	436	9738426	6	3.94077
5	9757621	9757950	330	9757657	9	4.98189
5	9767908	9768253	346	9768020	9	5.7264
5	9911583	9911941	359	9911865	7	4.10627
5	10323692	10324220	529	10323850	10	5.80609
5	10427595	10428157	563	10427705	6	3.3206
5	10673584	10673970	387	10673648	7	4.03317
5	11304202	11304511	310	11304214	6	3.48566
5	11368338	11368719	382	11368600	7	3.76048
5	11423280	11423625	346	11423497	7	4.97847
5	11454647	11455025	379	11454929	7	4.97847
5	11476769	11477142	374	11476800	7	5.47978
5	11498583	11498916	334	11498766	8	5.56774

5	11720566	11721181	616	11721086	8	3.28682
5	11816946	11817259	314	11817152	8	4.89911
5	12217279	12217586	308	12217481	8	3.28682
5	12254602	12255260	659	12255090	7	4.06945
5	14217219	14217543	325	14217404	8	3.51085
5	14297841	14298222	382	14298139	7	3.45771
5	14311215	14311744	530	14311615	7	3.1897
5	14500564	14500880	317	14500718	8	4.58252
5	14914647	14914991	345	14914941	12	3.12881
5	14933403	14934007	605	14933701	36	6.26395
5	14948754	14949174	421	14948813	27	4.7971
5	14959310	14959746	437	14959428	41	4.36845
5	14990918	14991242	325	14991009	22	4.87838
5	15006501	15006895	395	15006724	40	6.06244
5	15026089	15026481	393	15026229	37	5.93086
5	15042535	15042938	404	15042640	55	15.99195
5	15051677	15052029	353	15051732	39	5.68027
5	15539791	15540288	498	15539933	15	3.98281
5	15598699	15599065	367	15598762	20	5.12562
5	15651202	15651557	356	15651443	25	3.37308
5	15655143	15655452	310	15655306	20	4.17295
5	15663220	15663856	637	15663329	20	6.33344
5	15677908	15678562	655	15678461	28	3.04249
5	15689530	15690015	486	15689777	46	6.67817
5	15703313	15703634	322	15703461	32	5.36398
5	17779138	17779479	342	17779298	8	4.94122
5	18010354	18010654	301	18010371	9	3.78016
5	18226062	18226410	349	18226273	10	5.27216
5	18412924	18413234	311	18413221	7	3.57424
5	18490447	18491124	678	18490941	7	4.77975
5	18850295	18850617	323	18850579	7	3.45771
5	19048656	19049044	389	19049003	7	4.65491
5	19841611	19842166	556	19842112	7	5.30623
5	20356001	20356319	319	20356013	10	4.65057
5	20403701	20404057	357	20403863	7	5.47978
5	20460788	20461529	742	20460978	9	6.87393
5	20701531	20701864	334	20701842	7	3.45771
5	20735369	20735690	322	20735589	6	3.8181
5	21801708	21802055	348	21801967	7	5.47978
5	22343057	22343426	370	22343066	6	3.79171
5	23559827	23560160	334	23559855	7	3.82588
5	26029310	26029679	370	26029501	13	5.45117
5	26041652	26042005	354	26041763	8	5.56774
5	26502047	26502354	308	26502178	6	4.50291
5	27380968	27381339	372	27381257	5	3.49869
5	27438254	27438871	618	27438370	5	3.5422
5	27495555	27495887	333	27495808	5	3.58241
5	28633621	28633940	320	28633788	7	4.50662

5	28777291	28777967	677	28777863	6	3.79171
5	29448417	29448757	341	29448552	6	4.30352
5	35115519	35115825	307	35115538	5	3.29435
5	35542348	35542695	348	35542465	6	3.89915
5	37038435	37038739	305	37038659	7	5.47978
5	37147279	37147581	303	37147465	5	3.58241
5	37869739	37870041	303	37869810	6	3.77863
5	38430447	38430809	363	38430650	8	4.94122
5	38471842	38472152	311	38472060	7	4.65491
5	39227524	39227890	367	39227768	7	5.47978
5	39389560	39389888	329	39389705	7	5.03049
5	39981598	39981984	387	39981689	8	5.71006
5	40101831	40102147	317	40101863	7	5.36479
5	40361914	40362228	315	40362166	6	4.45522
5	40388733	40389147	415	40388979	7	5.0835
5	40419540	40419920	381	40419617	6	4.45522
5	40428100	40428412	313	40428216	8	4.8576
5	40511945	40512313	369	40512104	7	5.42458
5	40642801	40643476	676	40643287	8	5.60272
5	40856652	40857003	352	40856930	8	6.44394
5	40919702	40920075	374	40919983	6	3.19716
5	41083741	41084046	306	41083762	6	3.89915
5	41410212	41410546	335	41410426	7	4.5946
5	41554343	41554746	404	41554671	6	3.66421
5	41602029	41602496	468	41602215	8	6.50668
5	42263741	42264189	449	42264080	6	4.02633
5	43610530	43610917	388	43610590	5	3.25592
5	44089432	44089943	512	44089766	6	4.16086
5	44158731	44159063	333	44158881	7	4.22
5	44426114	44426418	305	44426131	6	3.79171
5	44879912	44880235	324	44880105	6	4.50291
5	45153087	45153647	561	45153155	6	3.89915
5	45336031	45336517	487	45336368	5	3.1094
5	46372753	46373057	305	46372802	6	4.45522
5	46550097	46550711	615	46550183	6	3.94077
5	46643334	46643682	349	46643492	10	6.58426
5	46659286	46659585	300	46659358	5	3.29435
5	47968911	47969215	305	47969037	7	5.24887
5	49058656	49059020	365	49058958	9	6.93917
5	49308713	49309130	418	49308895	8	4.94122
5	49574576	49574898	323	49574598	7	4.10626
5	50321293	50321724	432	50321425	8	3.28682
5	50354556	50354878	323	50354633	8	5.44836
5	50468217	50468542	326	50468331	7	5.13754
5	50805549	50806110	562	50805967	7	3.89308
5	52029477	52029816	340	52029499	6	4.07032
5	53153070	53153422	353	53153168	7	4.82809
5	53871334	53871640	307	53871342	6	3.02469

5	54362236	54362625	390	54362428	7	4.50662
5	54430939	54431263	325	54430942	5	3.4561
5	54506977	54507289	313	54506999	8	4.19779
5	54558606	54559097	492	54558798	6	3.79171
5	54582729	54583056	328	54582863	8	5.3014
5	54686175	54686989	815	54686295	10	5.80609
5	55014250	55014560	311	55014361	6	4.50291
5	55053510	55053834	325	55053677	8	4.19779
5	55356887	55357221	335	55357160	9	6.11997
5	56078126	56078473	348	56078197	7	4.29872
5	56114470	56114792	323	56114637	8	4.98393
5	56163527	56164018	492	56163818	9	3.94891
5	56178690	56179132	443	56178928	10	6.33777
5	56600282	56600607	326	56600523	7	4.77975
5	56713696	56714673	978	56714086	9	4.98189
5	56844263	56844599	337	56844292	7	5.47978
5	57317092	57317408	317	57317256	9	5.37431
5	57451890	57452255	366	57452106	9	6.33515
5	57572647	57573078	432	57572797	9	5.54566
5	58722250	58722586	337	58722291	7	3.76048
5	58753598	58753920	323	58753850	9	4.03728
5	58932198	58932621	424	58932220	6	3.77863
5	59010534	59010930	397	59010646	7	3.45771
5	59012825	59013189	365	59012945	7	4.29872
5	59197325	59197683	359	59197634	6	3.89915
5	59210436	59210737	302	59210668	7	3.1897
5	59446803	59447144	342	59446976	9	6.33515
5	59600290	59600797	508	59600483	9	3.94891
5	59686522	59686837	316	59686785	5	3.4561
5	59825205	59825526	322	59825388	9	6.33515
5	59896927	59897506	580	59897354	9	6.11997
5	59962330	59962784	455	59962478	7	5.47978
5	60518470	60518837	368	60518553	7	4.82809
5	61310710	61311019	310	61310833	7	3.45771
5	61564475	61564926	452	61564800	6	3.35262
5	61911422	61911739	318	61911452	5	3.21823
5	62038753	62039073	321	62038869	6	4.50291
5	62039673	62040036	364	62039806	6	3.79171
5	63182307	63182694	388	63182620	8	4.58252
5	64012592	64012892	301	64012629	5	3.58241
5	65124632	65125004	373	65124688	6	3.66421
5	71225214	71225534	321	71225378	6	3.89915
5	71231785	71232103	319	71231824	6	4.50291
5	71710489	71710831	343	71710685	7	4.87731
5	72237600	72237946	347	72237920	6	4.25501
5	73588185	73588875	691	73588694	6	4.50291
5	73918337	73918676	340	73918427	6	3.77863
5	75004966	75005286	321	75005099	8	6.44394

5	76239123	76239422	300	76239276	6	4.25501
5	78123186	78123572	387	78123316	10	5.80609
5	78321144	78321445	302	78321323	5	3.58241
5	79758302	79758720	419	79758709	6	4.50291
5	80895507	80895855	349	80895586	6	3.79171
5	84894255	84894614	360	84894574	6	3.79171
5	86374987	86375302	316	86375152	5	3.58241
5	88319962	88320491	530	88319992	5	3.29435
5	88584960	88585365	406	88585165	9	6.93917
5	89208034	89208379	346	89208192	7	3.45771
5	89398628	89398928	301	89398897	5	3.37357
5	89678082	89678424	343	89678209	7	5.47978
5	90475515	90475934	420	90475786	7	3.45771
5	94663595	94663895	301	94663707	7	5.47978
5	96057367	96057689	323	96057602	6	4.20747
5	100291828	100292155	328	100291979	7	5.47978
5	102524376	102524724	349	102524440	7	5.47978
5	107326888	107327194	307	107326982	6	4.50291
5	120673442	120673745	304	120673732	7	4.97847
5	125701809	125702124	316	125701851	5	3.58241
5	127727122	127727658	537	127727634	6	4.50291
5	136035291	136035611	321	136035493	7	5.47978
5	140362848	140363177	330	140363005	6	4.50291
5	145452847	145453217	371	145452933	7	5.47978
5	145480059	145480419	361	145480248	6	4.07032
5	146038952	146039274	323	146039084	8	5.93643
5	147670778	147671175	398	147671162	6	4.20747
5	149255149	149255487	339	149255240	5	3.58241
5	150791068	150791641	574	150791484	8	5.82121
6	3123651	3124111	461	3123761	5	3.1094
6	3980219	3980640	422	3980259	7	4.10626
6	6792283	6792737	455	6792450	7	4.82809
6	7383100	7383497	398	7383351	6	4.50291
6	7921933	7922254	322	7922109	7	4.10626
6	8315927	8316248	322	8316150	7	5.36479
6	9573841	9574148	308	9574023	7	4.65491
6	9650241	9650606	366	9650508	5	3.5422
6	9706425	9706741	317	9706617	6	4.50291
6	9777237	9777544	308	9777424	6	4.50291
6	10721995	10722324	330	10722238	6	4.50291
6	12240555	12240871	317	12240646	5	3.58241
6	13234825	13235131	307	13234958	6	3.70171
6	13453641	13453965	325	13453854	6	4.50291
6	13530478	13530798	321	13530752	6	4.07032
6	14990896	14991239	344	14991029	6	4.25501
6	15001614	15002100	487	15001841	7	5.47978
6	15578947	15579248	302	15579234	6	4.50291
6	16810526	16810886	361	16810692	6	3.98315

6	16814427	16814739	313	16814471	6	3.73984
6	17537786	17538226	441	17537928	5	3.33356
6	17708995	17709331	337	17709130	6	3.79171
6	18583410	18583840	431	18583728	6	3.79171
6	20855438	20855757	320	20855494	6	4.30352
6	22050636	22050942	307	22050726	7	4.65491
6	23366892	23367205	314	23367055	8	6.50668
6	23368921	23369267	347	23368930	7	5.47978
6	23585519	23585879	361	23585651	5	3.58241
6	26119834	26120156	323	26120040	7	5.47978
6	26204499	26204866	368	26204586	7	5.47978
6	27368071	27368381	311	27368352	8	3.88945
6	33331489	33331809	321	33331762	6	3.85827
6	34338855	34339468	614	34339120	7	5.13754
6	34544038	34544341	304	34544213	6	3.79171
6	35929634	35929994	361	35929770	8	6.30939
6	36329883	36330690	808	36330625	8	6.50668
6	37725654	37726178	525	37725885	5	3.29435
6	40104690	40105015	326	40104750	7	5.42458
6	43170580	43170979	400	43170700	7	5.36479
6	43228763	43229133	371	43228902	8	6.24414
6	45473691	45474184	494	45473826	6	4.50291
6	45900082	45900524	443	45900165	7	5.47978
6	48882498	48882862	365	48882516	6	3.79171
6	50811217	50811529	313	50811403	6	4.50291
6	55698035	55698366	332	55698182	6	4.50291
6	56631549	56631902	354	56631620	6	3.3206
6	59439461	59439778	318	59439592	6	4.50291
6	60824147	60824447	301	60824295	7	4.65491
6	61027490	61027829	340	61027681	7	5.36479
6	62133488	62133829	342	62133499	5	3.04015
6	62264865	62265225	361	62264976	8	4.94122
6	62317263	62317708	446	62317650	7	4.6397
6	62348751	62349691	941	62348861	8	5.49896
6	62672381	62672786	406	62672674	7	5.47978
6	63036551	63036866	316	63036818	9	5.37431
6	63417636	63417944	309	63417844	8	4.19779
6	63599941	63600264	324	63600146	6	3.77863
6	63784551	63784894	344	63784695	9	6.33515
6	63892689	63893014	326	63892901	6	4.50291
6	64130392	64131014	623	64130884	5	3.18126
6	64453954	64454370	417	64454260	7	5.30623
6	65032552	65033052	501	65032691	5	3.5422
6	65250718	65251134	417	65250983	7	4.97847
6	65303006	65303513	508	65303369	5	3.4561
6	66199206	66199561	356	66199465	7	5.47978
6	66564838	66565220	383	66565019	5	3.21823
6	67461285	67461620	336	67461499	7	4.18152

6	67654172	67654489	318	67654173	7	3.45771
6	68291411	68291729	319	68291627	6	4.50291
6	69518578	69518895	318	69518699	8	6.50668
6	70002500	70002866	367	70002810	5	3.58241
6	70949175	70949501	327	70949196	6	4.50291
6	70995871	70996210	340	70996023	7	5.47978
6	71617808	71618124	317	71617945	7	5.03049
6	71944653	71945225	573	71944863	5	3.58241
6	75943412	75943814	403	75943548	8	6.50668
6	76364610	76364932	323	76364710	7	4.97847
6	78844877	78845233	357	78844917	5	3.58241
6	79280160	79280534	375	79280210	8	6.50668
6	80932210	80932513	304	80932332	8	6.50668
6	81052006	81052437	432	81052025	6	3.79171
6	92424725	92425034	310	92424966	9	3.94891
6	92600955	92601344	390	92601040	6	3.79171
6	93426424	93426802	379	93426530	7	4.65491
6	93468677	93469019	343	93468707	5	3.58241
6	94824537	94824881	345	94824670	8	6.50668
6	96073840	96074184	345	96074117	6	4.50291
6	97721104	97721435	332	97721317	6	4.45522
6	103761400	103761708	309	103761409	5	3.5422
6	103909128	103909477	350	103909314	6	4.50291
6	104812523	104812890	368	104812533	6	3.79171
6	105668034	105668346	313	105668241	6	4.50291
6	109437516	109437837	322	109437787	6	3.79171
6	109763982	109764302	321	109764045	6	4.50291
6	111162432	111162736	305	111162437	5	3.58241
6	114184144	114184497	354	114184438	6	4.50291
6	116819443	116819816	374	116819574	6	4.50291
6	122100680	122101204	525	122100745	6	4.50291
6	122195214	122195680	467	122195396	6	4.50291
6	123184414	123184717	304	123184701	5	3.58241
6	123261639	123262003	365	123261688	6	3.79171
6	128185343	128185653	311	128185462	8	6.50668
6	128682700	128683081	382	128682927	7	5.24887
6	130034498	130034797	300	130034609	5	3.29435
6	130101019	130101332	314	130101270	6	4.50291
6	131544696	131545001	306	131544795	6	4.50291
6	132337411	132337725	315	132337414	7	3.45771
6	132839519	132840090	572	132839725	10	8.69114
6	138940584	138940902	319	138940593	5	3.58241
6	138943586	138944006	421	138943755	8	6.50668
6	139846951	139847276	326	139847201	6	4.50291
6	140388531	140388848	318	140388587	6	4.50291
6	140866210	140866600	391	140866512	6	4.30352
6	141241848	141242258	411	141241955	7	5.47978
6	141444879	141445464	586	141444898	7	3.45771

6	144586892	144587282	391	144586910	6	3.79171
6	145580089	145580416	328	145580146	5	3.58241
6	146920957	146921320	364	146921015	7	5.30623
7	7682993	7683566	574	7683547	6	3.3206
7	11372588	11372894	307	11372704	7	5.47978
7	12010482	12010790	309	12010587	12	4.31384
7	15297424	15297751	328	15297511	5	3.58241
7	15447727	15448026	300	15447882	6	4.30352
7	15463632	15464036	405	15463808	8	6.50668
7	15990297	15990774	478	15990596	6	4.50291
7	18488404	18488734	331	18488581	10	6.74034
7	20813879	20814184	306	20814097	5	3.58241
7	22214937	22215261	325	22215119	6	3.79171
7	22759468	22759956	489	22759711	7	5.47978
7	31470155	31470496	342	31470171	5	3.58241
7	31889722	31890076	355	31889880	5	3.58241
7	38184417	38184783	367	38184587	12	5.60636
7	41964394	41964721	328	41964532	5	3.58241
7	43158918	43159372	455	43159089	6	4.50291
7	43389236	43389575	340	43389547	6	4.50291
7	47619100	47619454	355	47619130	5	3.58241
7	48201880	48202215	336	48202005	5	3.58241
7	51355076	51355391	316	51355217	6	4.50291
7	55225985	55226297	313	55226202	7	3.45771
7	56581053	56581378	326	56581061	7	4.65491
7	59318346	59318707	362	59318578	6	3.52023
7	59768280	59768629	350	59768561	16	4.12922
7	60226568	60226900	333	60226689	6	4.50291
7	61880389	61880774	386	61880460	5	3.58241
7	62535959	62536280	322	62535981	7	3.45771
7	62959639	62960051	413	62959937	5	3.58241
7	63552259	63552572	314	63552278	6	3.79171
7	64488673	64488987	315	64488896	9	4.98189
7	74838260	74838619	360	74838458	7	4.65491
7	75739357	75739682	326	75739524	6	4.50291
7	76358194	76358500	307	76358430	8	5.39858
7	79677265	79677657	393	79677515	5	3.29435
7	80604253	80604584	332	80604324	7	4.65491
7	84063770	84064097	328	84063934	6	4.50291
7	84783518	84783822	305	84783724	7	5.47978
7	85303835	85304161	327	85304109	6	4.50291
7	86061665	86062018	354	86061917	6	4.50291
7	86621999	86622355	357	86622155	6	4.50291
7	87219916	87220216	301	87220108	8	6.18019
7	87563118	87563423	306	87563233	5	3.21823
7	88509163	88509464	302	88509293	6	4.50291
7	89725605	89725918	314	89725730	5	3.5422
7	90165495	90165809	315	90165629	7	5.24887

7	91376525	91376849	325	91376714	8	5.49896
7	92729736	92730116	381	92729886	6	4.07032
7	93926763	93927125	363	93926769	6	3.79171
7	94046332	94046665	334	94046348	5	3.37357
7	94096162	94096767	606	94096670	9	4.32007
7	94847392	94847713	322	94847456	8	4.54529
7	95014405	95014746	342	95014562	6	4.07032
7	97221936	97222265	330	97222161	5	3.4561
7	97910086	97910435	350	97910283	8	6.50668
7	105118256	105118623	368	105118310	7	5.47978
7	105201462	105201821	360	105201718	6	3.79171
7	105422808	105423201	394	105423135	6	3.66421
7	106630352	106630674	323	106630453	5	3.58241
7	107927566	107927870	305	107927815	7	5.47978
7	113428224	113428527	304	113428454	5	3.58241
7	113572063	113572366	304	113572223	7	4.10626
7	115843210	115843541	332	115843383	6	4.25501
7	120485923	120486242	320	120486048	6	4.25501
7	120918675	120918977	303	120918863	6	3.94077
7	130591992	130592375	384	130592069	9	6.52524
7	132303099	132303460	362	132303334	7	5.47978
7	145340963	145341449	487	145341286	30	21.28436
8	3012532	3012862	331	3012673	5	3.1094
8	4685122	4685478	357	4685429	10	6.95899
8	5145405	5145927	523	5145749	6	4.40359
8	5649468	5649823	356	5649739	7	4.68558
8	6076889	6077198	310	6077095	8	5.39858
8	6434390	6434701	312	6434688	8	5.39858
8	6601959	6602378	420	6602183	8	5.56774
8	6992360	6992714	355	6992475	5	3.07446
8	9421011	9421360	350	9421153	8	6.50668
8	9617173	9617506	334	9617466	7	5.47978
8	10025585	10025902	318	10025757	6	3.79171
8	15707330	15707634	305	15707534	5	3.4561
8	16013106	16013416	311	16013286	6	3.79171
8	16338998	16339323	326	16339175	7	5.47978
8	16913079	16913430	352	16913197	7	5.36479
8	17726464	17726812	349	17726631	7	3.99741
8	18162826	18163128	303	18162945	7	5.47978
8	19801663	19802037	375	19801881	14	5.35226
8	19934095	19934607	513	19934394	23	3.25325
8	20041636	20042350	715	20042135	17	4.64765
8	20065553	20065930	378	20065675	19	6.43863
8	20106418	20106768	351	20106489	24	6.38156
8	20116922	20117267	346	20117067	24	7.33297
8	20273579	20274366	788	20274064	27	5.19134
8	20331774	20332183	410	20332050	30	5.28759
8	20515825	20516636	812	20516112	27	6.08395

8	20520461	20520874	414	20520587	43	12.36678
8	20542779	20543134	356	20543025	22	4.23158
8	20577257	20577801	545	20577519	24	3.94537
8	20612655	20612991	337	20612752	26	4.04456
8	20620443	20620795	353	20620505	19	5.09378
8	20626050	20626399	350	20626193	30	7.6031
8	20652427	20652760	334	20652501	17	4.64765
8	20687265	20687813	549	20687301	20	5.12562
8	20867621	20868079	459	20867935	6	4.07032
8	21955597	21955931	335	21955619	8	4.19779
8	28160257	28160723	467	28160532	7	5.47978
8	28187493	28187802	310	28187747	6	4.50291
8	28677277	28677652	376	28677324	5	3.58241
8	29916253	29916578	326	29916309	6	4.16086
8	30905539	30905869	331	30905674	5	3.49869
8	32274601	32274904	304	32274810	6	4.07032
8	36983420	36983752	333	36983596	7	4.65491
8	37566032	37566425	394	37566379	8	4.94122
8	37712144	37712513	370	37712341	8	4.19779
8	41063710	41064177	468	41063839	7	4.82809
8	41888394	41888849	456	41888700	7	3.45771
8	41977280	41977640	361	41977301	7	5.13754
8	43501649	43501954	306	43501943	9	3.19498
8	50055765	50056086	322	50055781	7	4.5946
8	50640763	50641069	307	50640913	6	3.8181
8	50807494	50807854	361	50807841	7	4.92742
8	50983696	50984028	333	50983697	6	3.79171
8	51068179	51068634	456	51068434	7	5.47978
8	52048013	52048367	355	52048145	7	5.47978
8	52311296	52311606	311	52311362	6	4.50291
8	52389875	52390178	304	52390090	6	3.66421
8	52484480	52484847	368	52484743	10	5.80609
8	52541392	52541726	335	52541693	6	3.98315
8	52637330	52637699	370	52637342	5	3.18126
8	52689412	52689730	319	52689646	5	3.4561
8	53053974	53054280	307	53054163	6	3.94077
8	53062175	53062504	330	53062369	7	4.82809
8	53189316	53189641	326	53189537	7	4.82809
8	53807844	53808471	628	53808093	7	4.92742
8	54926648	54927235	588	54927200	7	4.82809
8	55121274	55121592	319	55121458	29	7.64125
8	55869977	55870345	369	55870288	6	4.07032
8	56074291	56074643	353	56074393	6	4.50291
8	56345083	56345390	308	56345117	6	4.50291
8	56879161	56879477	317	56879278	5	3.41441
8	57735671	57736008	338	57735881	8	6.50668
8	57968367	57968872	506	57968698	6	4.25501
8	57976764	57977105	342	57976955	6	4.50291

8	57977668	57977968	301	57977726	8	5.56774
8	58023985	58024334	350	58024207	7	3.45771
8	58476343	58476703	361	58476569	8	5.87829
8	58654183	58654496	314	58654391	9	6.93917
8	58732570	58733065	496	58732638	8	4.19779
8	59224747	59225117	371	59224950	7	4.10626
8	60092743	60093282	540	60093104	8	6.50668
8	60275264	60275615	352	60275295	6	3.3206
8	60686133	60686490	358	60686322	5	3.58241
8	62208344	62208678	335	62208509	8	6.50668
8	62652382	62652748	367	62652542	16	8.12061
8	63488685	63489036	352	63488870	8	5.11589
8	63516042	63516446	405	63516206	5	3.58241
8	63585609	63585980	372	63585687	8	5.76514
8	63695196	63695545	350	63695338	7	4.97847
8	63749651	63749972	322	63749942	6	3.79171
8	63765305	63765796	492	63765581	8	6.30939
8	65407079	65407569	491	65407402	6	4.35303
8	65408718	65409027	310	65408869	9	6.93917
8	65642882	65643235	354	65643148	6	3.8181
8	66238113	66238418	306	66238349	6	3.62733
8	66385581	66385895	315	66385801	6	4.20747
8	66518607	66518951	345	66518622	5	3.58241
8	66654773	66655089	317	66654935	9	6.52524
8	67829308	67829642	335	67829556	7	3.76048
8	69253126	69253447	322	69253402	6	3.79171
8	69311903	69312209	307	69312020	8	4.19779
8	71807150	71807656	507	71807197	10	4.54498
8	73073983	73074369	387	73074025	6	4.50291
8	73547370	73547734	365	73547370	8	3.28682
8	74143205	74143507	303	74143414	7	5.47978
8	74366794	74367120	327	74367107	6	3.66421
8	75663106	75663559	454	75663317	6	3.79171
8	76054971	76055287	317	76055247	6	3.02469
8	76214967	76215279	313	76215019	5	3.00646
8	76906053	76906396	344	76906257	8	6.50668
8	77175229	77175590	362	77175531	6	4.16086
8	77275897	77276232	336	77276211	6	4.50291
8	78033381	78033869	489	78033384	7	3.45771
8	78431213	78431517	305	78431369	6	4.30352
8	79847332	79847689	358	79847539	6	3.79171
8	80083963	80084264	302	80084222	7	4.50662
8	80242915	80243287	373	80243195	6	4.50291
8	80339082	80339723	642	80339135	7	4.339
8	81142313	81142643	331	81142339	8	3.28682
8	81190767	81191074	308	81190988	8	5.60272
8	81460196	81460501	306	81460472	7	3.45771
8	81539775	81540143	369	81539801	5	3.58241

8	82423575	82423925	351	82423764	7	4.82809
8	82534451	82534925	475	82534761	7	5.19265
8	90706605	90706949	345	90706859	8	6.50668
8	91014910	91015240	331	91014964	6	3.73984
8	91473442	91473755	314	91473684	6	4.45522
8	91745193	91745534	342	91745278	6	4.50291
8	93504423	93504795	373	93504537	8	5.93643
8	96205426	96205882	457	96205802	6	4.20747
8	96742441	96742774	334	96742765	8	4.19779
8	96901957	96902283	327	96901981	5	3.58241
8	96942352	96942692	341	96942409	7	5.47978
8	96947626	96947972	347	96947901	6	3.89915
8	97224062	97224450	389	97224184	5	3.58241
8	97784778	97785149	372	97784888	8	6.50668
8	97826115	97826567	453	97826260	5	3.00646
8	98560486	98561011	526	98560957	6	4.50291
8	98835622	98835959	338	98835827	5	3.21823
8	99048814	99049149	336	99049014	5	3.04015
8	99240270	99240632	363	99240280	6	3.79171
8	99615125	99615475	351	99615235	5	3.29435
8	100040232	100040647	416	100040552	7	5.47978
8	100684145	100684479	335	100684397	7	5.47978
8	101224584	101224932	349	101224653	9	6.93917
8	101501101	101501459	359	101501200	9	7.57852
8	101514880	101515364	485	101515006	6	3.98315
8	101670952	101671274	323	101671168	9	6.87393
8	101878976	101879305	330	101879252	8	6.50668
8	103119378	103120074	697	103119513	5	3.58241
8	104224664	104225049	386	104224815	7	5.47978
8	107544892	107545267	376	107545137	7	4.97847
8	107761559	107761891	333	107761586	7	5.47978
8	112513445	112513832	388	112513446	7	3.45771
8	112928421	112928774	354	112928721	7	5.47978
8	122901840	122902144	305	122902033	6	3.70171
8	123289986	123290381	396	123290015	6	4.50291
8	125330985	125331352	368	125331020	5	3.58241
8	126013696	126013995	300	126013880	7	5.30623
8	128076813	128077130	318	128076926	7	4.6397
8	128870081	128870398	318	128870241	8	4.23065
8	128991271	128991587	317	128991401	8	5.49896
9	3000272	3000628	357	3000489	3637	173.2421
9	3001143	3001479	337	3001374	1305	21.6053
9	3002027	3002332	306	3002136	2034	36.96147
9	3003330	3003696	367	3003430	981	56.78593
9	3010802	3011117	316	3010970	323	30.94559
9	3013157	3013514	358	3013266	353	24.30591
9	3014042	3014348	307	3014150	780	26.81595
9	3017812	3018675	864	3018578	1512	141.16684

9	3019916	3021127	1212	3020930	792	14.54441
9	3024125	3024547	423	3024480	1558	43.65961
9	3025477	3025922	446	3025734	1325	145.7924
9	3029458	3029809	352	3029747	954	17.81334
9	3032075	3032722	648	3032429	1489	63.67994
9	3034641	3035222	582	3035082	1409	69.84364
9	3037850	3038231	382	3038022	1097	111.33739
9	3676894	3677217	324	3677031	8	5.56774
9	5673940	5674258	319	5673976	7	5.03049
9	5989268	5990312	1045	5989997	7	4.73225
9	7087363	7087686	324	7087525	6	4.40359
9	8279237	8279543	307	8279397	7	5.47978
9	10546595	10547118	524	10546727	5	3.58241
9	10930917	10931232	316	10930980	6	3.79171
9	11413570	11413949	380	11413618	6	4.50291
9	11606997	11607316	320	11607191	6	4.50291
9	11825018	11825434	417	11825163	7	5.47978
9	12196620	12196966	347	12196699	5	3.41441
9	12435968	12436276	309	12436057	6	4.50291
9	16775457	16775773	317	16775691	6	4.50291
9	17739413	17739724	312	17739708	7	3.76048
9	18441967	18442305	339	18442035	8	6.50668
9	25852819	25853123	305	25853082	8	4.19779
9	26360099	26360432	334	26360176	5	3.58241
9	28836577	28836904	328	28836872	6	3.79171
9	31523806	31524125	320	31524011	6	4.50291
9	32232157	32232481	325	32232178	5	3.14499
9	32342571	32342884	314	32342872	6	3.79171
9	32355166	32355493	328	32355361	23	23.12246
9	32356025	32356374	350	32356226	22	24.33764
9	33317208	33317589	382	33317312	8	6.11749
9	36003847	36004588	742	36003942	6	3.79171
9	37889668	37889981	314	37889895	6	4.50291
9	38017236	38017593	358	38017304	5	3.58241
9	38178721	38179228	508	38178910	5	3.21823
9	38322598	38322915	318	38322633	5	3.58241
9	39372523	39372862	340	39372544	6	3.79171
9	39911591	39911898	308	39911684	6	3.66421
9	49281980	49282302	323	49282194	6	4.50291
9	52365595	52365917	323	52365754	7	5.47978
9	52438488	52438809	322	52438666	7	4.82809
9	53962678	53962986	309	53962898	7	5.47978
9	55074676	55075123	448	55074914	8	6.50668
9	63532906	63533209	304	63533005	5	3.58241
9	68538869	68539197	329	68539154	7	5.47978
9	72238480	72238892	413	72238495	6	3.3206
9	73457254	73457575	322	73457368	7	4.97847
9	75831804	75832327	524	75832028	5	3.41441

9	76353280	76353620	341	76353512	7	4.65491
9	76519387	76519692	306	76519532	5	3.58241
9	77203879	77204222	344	77204017	6	4.50291
9	77463774	77464086	313	77463839	6	4.50291
9	82332894	82333244	351	82333000	7	3.45771
9	82517569	82517885	317	82517649	7	4.77975
9	84558340	84558706	367	84558477	9	6.28009
9	86299205	86299570	366	86299422	6	4.02633
9	87653423	87653736	314	87653616	6	4.50291
9	87893864	87894201	338	87894163	6	4.07032
9	87969950	87970256	307	87970119	6	4.40359
9	88893442	88893793	352	88893728	6	3.94077
9	89204886	89205306	421	89204986	7	4.73225
9	89572593	89572946	354	89572808	6	4.50291
9	90451439	90451746	308	90451567	7	4.65491
9	90547335	90547710	376	90547514	8	5.56774
9	91424351	91424663	313	91424359	5	3.21823
9	92696133	92696453	321	92696366	8	5.56774
9	97036204	97036550	347	97036283	6	4.50291
9	97641474	97641805	332	97641617	6	4.50291
9	98612093	98612422	330	98612274	6	4.50291
9	107216264	107216596	333	107216389	9	7.28429
9	111593008	111593331	324	111593160	7	5.47978
9	114252341	114252877	537	114252468	9	5.68029
9	114338815	114339131	317	114338910	6	4.20747
9	117224449	117224750	302	117224510	5	3.58241
9	121939383	121939705	323	121939659	5	3.58241
9	123461821	123462192	372	123461990	196	112.01627
9	123832262	123832575	314	123832295	5	3.58241
9	124022784	124023107	324	124022973	7	5.47978
CHR_M	46157154	46157522	369	46157386	10	7.52331
CHR_M	15470375	15470735	361	15470501	29	8.25048
CHR_M	15512912	15513259	348	15512948	18	3.69949
CHR_M	15585559	15585895	337	15585655	8	4.65848
CHR_M	15610204	15610570	367	15610446	17	5.94093
GL4562	38985	39348	364	39142	7	4.6397
GL4562	84862	85253	392	85029	8	5.56774
GL4562	86666	86968	303	86771	7	4.97847
GL4562	171451	171862	412	171591	11	4.44564
GL4563	826	1421	596	1291	7	5.47978
GL4563	5626	6154	529	5692	5	3.5422
JH5843	68550	69734	1185	69629	505	38.53638
JH5843	70742	71359	618	71040	412	37.88798
JH5843	73079	73442	364	73175	856	74.33173
JH5843	75590	76063	474	75922	491	40.39774
JH5843	77981	79487	1507	79350	821	32.44351
JH5843	81266	81700	435	81588	411	81.95245
JH5843	83611	84212	602	83939	407	22.63985

JH5843	85491	85882	392	85777	449	22.7147
JH5843	87716	88394	679	88050	366	15.45717
JH5843	89374	91473	2100	90193	231	10.89761
JH5843	92746	93342	597	93096	445	21.20879
JH5843	94596	94994	399	94889	389	27.55641
JH5843	97099	97536	438	97456	159	5.60367
JH5843	100429	101303	875	101167	435	26.88577
JH5843	103222	104721	1500	103537	1007	80.72006
JH5843	107310	107613	304	107482	268	27.77179
JH5843	108902	109424	523	109191	651	47.91598
JH5843	110743	111128	386	111016	429	26.32745
JH5843	112993	113401	409	113286	834	54.48454
X	3724404	3724739	336	3724480	7	5.47978
X	4106015	4106427	413	4106262	8	6.11749
X	4355141	4355476	336	4355262	6	4.20747
X	4555527	4555845	319	4555751	8	6.50668
X	4953772	4954117	346	4953911	7	5.47978
X	5831414	5831828	415	5831655	6	4.50291
X	9745340	9745642	303	9745524	7	5.47978
X	23976681	23977081	401	23976843	9	7.57852
X	27996123	27996477	355	27996267	6	4.50291
X	28001760	28002119	360	28002103	5	3.58241
X	31200155	31200649	495	31200209	11	5.38864
X	31741300	31741820	521	31741678	9	4.63301
X	32178790	32179117	328	32178932	6	3.79171
X	32465153	32465471	319	32465424	8	5.56774
X	33537182	33537629	448	33537493	9	6.33515
X	33886531	33886848	318	33886538	5	3.49869
X	34462646	34462956	311	34462700	7	3.89308
X	35171775	35172145	371	35172002	5	3.58241
X	40225099	40225414	316	40225221	6	4.50291
X	43210314	43210656	343	43210475	7	5.47978
X	45478512	45478872	361	45478703	6	4.50291
X	49297737	49298084	348	49298077	6	3.79171
X	54405824	54406143	320	54406118	5	3.14499
X	54811583	54812108	526	54811887	6	3.79171
X	55003947	55004314	368	55004074	6	4.50291
X	55284140	55284494	355	55284311	9	6.52524
X	57547380	57547758	379	57547706	7	4.65491
X	59654351	59654659	309	59654507	6	4.50291
X	64493789	64494090	302	64493825	9	5.82058
X	66778212	66778562	351	66778271	6	4.50291
X	73244495	73244798	304	73244741	6	4.50291
X	76848835	76849180	346	76849015	7	5.47978
X	77652021	77652324	304	77652033	5	3.58241
X	79622818	79623563	746	79623521	7	4.65491
X	82252880	82253199	320	82252967	6	4.50291
X	92276000	92276324	325	92276138	6	4.50291

X	92402893	92403214	322	92402929	5	3.58241
X	99167067	99167372	306	99167104	6	4.50291
X	104680416	104680775	360	104680622	6	4.50291
X	107259116	107259497	382	107259453	7	4.65491
X	108759037	108759341	305	108759070	6	4.07032
X	113336795	113337213	419	113337106	8	6.50668
X	113912491	113912861	371	113912672	9	6.33515
X	115804999	115805346	348	115805167	6	4.50291
X	116603423	116603928	506	116603592	7	5.47978
X	116822989	116823312	324	116823157	7	5.47978
X	121045624	121045992	369	121045825	6	4.50291
X	121115583	121115892	310	121115719	6	4.50291
X	123446478	123446822	345	123446663	11	6.66709
X	123884338	123884652	315	123884358	11	3.71443
X	124351212	124351529	318	124351266	9	3.32934
X	124464201	124464568	368	124464302	9	7.57852
X	124590889	124591231	343	124590988	6	4.50291
X	124597312	124597655	344	124597475	7	5.30623
X	124672230	124672601	372	124672388	12	6.16038
X	125318347	125318675	329	125318397	6	4.50291
X	125353061	125353396	336	125353191	5	3.58241
X	125682992	125683330	339	125683256	9	4.98189
X	126070282	126070895	614	126070617	22	10.42991
X	126872015	126872339	325	126872151	7	5.47978
X	128062738	128063083	346	128063067	6	3.79171
X	128237071	128237411	341	128237325	12	7.56208
X	131904432	131904736	305	131904530	7	5.47978
X	132195238	132195559	322	132195332	5	3.5422
X	134648490	134648811	322	134648491	6	4.50291
X	135299682	135299995	314	135299775	6	4.11516
X	136162934	136163308	375	136163002	7	5.47978
X	139002894	139003213	320	139003115	6	4.07032
X	139462237	139462596	360	139462406	5	3.58241
X	145115109	145115460	352	145115323	14	7.7958
X	145956188	145956532	345	145956324	7	5.47978
X	146212714	146213156	443	146212896	6	4.50291
X	146842842	146843171	330	146843107	7	4.10626
X	148056811	148057160	350	148057042	5	3.58241
X	148120146	148120471	326	148120351	6	4.50291
X	148545112	148545413	302	148545238	6	4.50291
X	150011082	150011468	387	150011385	7	5.47978
X	157554345	157554839	495	157554470	7	5.47978
X	158213943	158214321	379	158214061	7	4.65491
X	160289533	160289888	356	160289713	6	4.50291
X	166256909	166257233	325	166257082	5	3.58241
Y	4148075	4148485	411	4148291	16	9.54129
Y	4150865	4151316	452	4151016	20	7.06624
Y	4610135	4610459	325	4610231	7	5.47978

Y	4757151	4757521	371	4757284	9	7.57852
Y	4826781	4827116	336	4826860	8	6.50668
Y	4837567	4837868	302	4837794	8	6.50668
Y	5686243	5686594	352	5686435	17	7.7367
Y	5711031	5711345	315	5711250	13	3.63773
Y	5759114	5759974	861	5759844	30	3.95823
Y	5826533	5826904	372	5826870	9	4.98189
Y	5881375	5881713	339	5881469	10	5.27216
Y	5947567	5947915	349	5947810	14	7.7958
Y	5965667	5965988	322	5965745	15	5.44265
Y	5968054	5968398	345	5968109	23	6.75467
Y	6047463	6047922	460	6047727	58	8.93134
Y	6052965	6053288	324	6053226	13	5.7029
Y	6065109	6065758	650	6065447	16	7.00083
Y	6070698	6071060	363	6070794	16	9.54129
Y	6087013	6087778	766	6087703	17	11.4215
Y	6131782	6132261	480	6131838	7	3.45771
Y	6216267	6216941	675	6216775	12	5.03261
Y	6237200	6237817	618	6237516	7	5.47978
Y	6285106	6285670	565	6285306	11	3.71443
Y	6300843	6301181	339	6301028	8	5.56774
Y	6365430	6366105	676	6365674	9	7.57852
Y	6524934	6525456	523	6525349	19	5.09378
Y	6530274	6530686	413	6530646	16	5.69185
Y	6546470	6546848	379	6546517	17	5.94093
Y	6612067	6612423	357	6612304	11	4.44564
Y	6675023	6675345	323	6675090	15	4.14377
Y	6679051	6679378	328	6679349	12	3.54844
Y	6741892	6742587	696	6742485	11	5.39948
Y	6758651	6758975	325	6758712	13	4.714
Y	6773698	6774035	338	6773990	14	4.82217
Y	6787373	6787738	366	6787661	16	4.12922
Y	6788719	6789176	458	6788972	30	8.0666
Y	6819939	6820259	321	6820074	16	3.72604
Y	6839967	6840318	352	6840264	14	4.17493
Y	7548286	7548594	309	7548518	6	4.50291
Y	7604637	7604961	325	7604887	11	3.71443
Y	8543654	8544012	359	8543909	18	5.18851
Y	8749128	8749657	530	8749378	11	9.84109
Y	8784831	8785203	373	8785183	8	5.56774
Y	8821342	8821743	402	8821451	16	5.32792
Y	8853193	8853572	380	8853378	18	8.49552
Y	8862138	8862456	319	8862185	6	4.50291
Y	8881225	8881535	311	8881320	9	4.98189
Y	8913437	8913796	360	8913456	8	5.87829
Y	8921931	8922868	938	8922701	10	8.69114
Y	8975042	8975360	319	8975087	15	4.73882
Y	9085733	9086070	338	9085739	11	3.71443

Y	9112450	9112765	316	9112533	8	5.56774
Y	9199426	9199845	420	9199677	12	6.2811
Y	9330042	9330397	356	9330330	8	3.28682
Y	9530632	9531222	591	9530907	10	4.65057
Y	9567451	9567754	304	9567544	7	4.65491
Y	9568368	9568709	342	9568591	7	4.65491
Y	9569167	9569527	361	9569435	7	4.65491
Y	10015044	10015411	368	10015127	13	7.9764
Y	10094840	10095176	337	10095048	9	4.98189
Y	10319341	10319837	497	10319810	15	5.07533
Y	10336396	10336718	323	10336590	13	6.96345
Y	10510346	10510709	364	10510617	8	6.50668
Y	10655934	10656257	324	10655946	6	4.50291
Y	12434109	12434435	327	12434140	6	4.50291
Y	13429852	13430198	347	13430118	7	5.47978
Y	13511110	13511557	448	13511450	6	3.79171
Y	13519103	13519484	382	13519247	6	4.50291
Y	13552777	13553113	337	13552803	7	4.10626
Y	16464089	16464400	312	16464251	10	4.65057
Y	17060595	17060923	329	17060760	10	5.41652
Y	18096960	18097305	346	18097072	11	8.55855
Y	18258380	18259079	700	18259047	6	3.66421
Y	19596527	19596868	342	19596532	8	3.28682
Y	20198206	20199003	798	20198685	8	4.19779
Y	21541231	21541574	344	21541406	7	4.65491
Y	21689626	21689979	354	21689848	8	6.50668
Y	23116921	23117276	356	23117023	6	4.50291
Y	34728945	34729296	352	34729245	9	3.94891
Y	37740428	37740774	347	37740519	8	6.50668
Y	38134807	38135196	390	38134817	6	4.50291
Y	39079212	39079551	340	39079271	9	4.63301
Y	39098665	39099056	392	39098812	14	6.56676
Y	40160927	40161264	338	40161088	10	4.65057
Y	40644608	40644929	322	40644834	6	4.50291
Y	40833702	40834014	313	40833932	11	6.66709
Y	40951384	40951722	339	40951436	5	3.58241
Y	41098160	41098500	341	41098411	6	4.50291
Y	41340324	41340669	346	41340421	7	4.65491
Y	41450989	41451293	305	41451126	8	5.56774
Y	41492475	41492804	330	41492635	8	5.56774
Y	41684742	41685359	618	41685074	12	4.31384
Y	43374751	43375120	370	43374773	11	3.71443
Y	43390944	43391247	304	43391118	5	3.58241
Y	43403736	43404088	353	43403873	8	6.50668
Y	43491644	43491973	330	43491649	10	3.72945
Y	43992297	43992602	306	43992351	8	6.50668
Y	45004586	45005060	475	45004793	8	5.56774
Y	46509518	46509843	326	46509699	11	4.44564

Y	47895355	47895693	339	47895502	6	4.50291
Y	67964013	67964359	347	67964080	7	3.45771
Y	67994108	67994431	324	67994133	7	5.47978
Y	68423359	68423684	326	68423424	7	5.47978
Y	74998525	74998922	398	74998651	8	6.50668
Y	89410240	89410627	388	89410387	6	4.50291
Y	89849025	89849797	773	89849702	14	6.56676

S4_CHIP-seq and RNA-seq overlap data			
#ID	preferredName	log2FC	regulated
ENSMUSG00000012428	Steap4	-7.517361395	down
ENSMUSG00000022912	Pros1	-10.22949046	down
ENSMUSG00000041695	Kcnj2	-3.105578029	down
ENSMUSG00000022707	Gbe1	-1.692641798	down
ENSMUSG00000064373	Sepp1	-2.765762098	down
ENSMUSG00000052353	Cemip	-1.678089661	down
ENSMUSG00000035158	Mitf	-2.227090976	down
ENSMUSG00000032374	Plod2	-1.332077032	down
ENSMUSG00000024924	Vldlr	-1.375450842	down
ENSMUSG00000060961	Slc4a4	-1.631462097	down
ENSMUSG00000029108	Pcdh7	-1.25513396	down
ENSMUSG00000030022	Adamts9	-1.350785515	down
ENSMUSG00000028015	Ctso	-1.721168394	down
ENSMUSG00000060459	Kng2	-2.417423866	down
ENSMUSG00000052331	Ankrd44	-1.347148941	down
ENSMUSG00000055322	Tns1	-1.119228301	down
ENSMUSG00000074794	Arrdc3	-1.199486687	down
ENSMUSG00000035109	Shc4	-1.06601923	down
ENSMUSG00000021196	Pfkp	-1.019749826	down
ENSMUSG00000075324	Fign	-1.489252747	down
ENSMUSG00000036334	Igsf10	-1.336741434	down
ENSMUSG00000053470	Kdm3a	-1.010491068	down
ENSMUSG00000052477	ENSMUSG00000052477	-1.324429832	down
ENSMUSG00000028926	Cdk14	-0.975897524	down
ENSMUSG00000031647	Mfap3l	-2.255717159	down
ENSMUSG00000038070	Cntln	-1.064321565	down
ENSMUSG00000022123	Scel	-3.256928421	down
ENSMUSG00000019947	Arid5b	-0.950446079	down
ENSMUSG00000002107	Celf2	-0.960239442	down
ENSMUSG00000030029	Lrig1	-0.89702451	down
ENSMUSG00000025986	Slc39a10	-0.93115745	down
ENSMUSG00000029648	Flt1	-0.971498895	down
ENSMUSG00000005534	Insr	-0.909098754	down
ENSMUSG00000024978	Gpam	-0.817891349	down
ENSMUSG00000056536	Pign	-0.778441363	down
ENSMUSG00000019997	Ctgf	1.796831517	up
ENSMUSG00000039316	Rftn1	3.757795113	up
ENSMUSG00000036256	Igfbp7	1.276285422	up
ENSMUSG00000022325	Pop1	1.249819309	up
ENSMUSG00000078497	Gm13145	1.957498468	up
ENSMUSG00000034892	Rps29	1.085496168	up
ENSMUSG00000022321	Cdh10	7.573961813	up
ENSMUSG00000027276	Jag1	1.308013124	up
ENSMUSG00000090854	Gm4340	3.093736382	up
ENSMUSG00000020375	Rufy1	1.063532123	up
ENSMUSG00000071052	Rpl7a-ps5	1.286514581	up
ENSMUSG00000027405	Nop56	0.803395842	up

S5_quality control

Type	Samples	Clean reads	Clean bases	GC Content	%≥Q30
RNA-seq	HT22	23,018,655	6,879,382,152	50.76%	91.18%
RNA-seq	Pura-KO	23,425,924	7,008,656,052	51.01%	91.89%
ChIP-seq	input	15041364	4512409200	41.72	83.57
ChIP-seq	Pura	13231301	3969390300	37.11	87.34
